# Event-Based Surveillance of Poisonings and Potentially Hazardous Exposures over 12 Months of the COVID-19 Pandemic

**DOI:** 10.3390/ijerph182111133

**Published:** 2021-10-22

**Authors:** Meghan A. Cook, Nicholas Brooke

**Affiliations:** 1 Centre for Radiation, Chemical and Environmental Hazards, Public Health England, Didcot OX11 0RQ, UK; nicholas.brooke@phe.gov.uk; 2 Global Health Security, Public Health England, London SE1 8UG, UK

**Keywords:** toxicovigilance, poisoning, COVID-19, misinformation, hand sanitiser, methanol, hydroxychloroquine, ivermectin, chlorine dioxide

## Abstract

The COVID-19 pandemic has seen people and governments utilise an array of chemical and pharmaceutical substances in an attempt to prevent and treat COVID-19 infections. The Centre for Radiation, Chemicals and Environmental Hazards (CRCE) at Public Health England (PHE) routinely undertakes Event-Based Surveillance (EBS) to monitor public health threats and incidents related to chemicals and poisons. From April 2020, EBS functions were expanded to screen international media for potentially hazardous exposures associated with the COVID-19 pandemic. Media sources reported that poisons centres were experiencing increased enquiries associated with the use and misuse of household cleaners and alcohol-based hand sanitiser (HS). There were also media reports of people self-medicating with over-the-counter supplements and traditional or herbal remedies. Public figures who directly or indirectly facilitated misinformation were sometimes reported to be associated with changes in poisoning trends. Border closures were also believed to have been associated with increasingly toxic illicit drug supplies in Canada, and record numbers of opioid-related deaths were reported. In other countries, where the sale of alcohol was banned or limited, home-brewing and methanol-based supplies resulted in a number of fatalities. At least two chemical incidents also occurred at industrial sites in India, after sites were left unattended or were closed and reopened due to lockdown measures. Reports of poisoning identified in the international media were provided to the UK National Poisons Information Service (NPIS) and contributed to the UK COVID-19 public health response.

## 1. Introduction

On the 30th of January 2020, the World Health Organization (WHO) announced the spread of COVID-19 across borders and the threat it posed to the health of populations constituted a Public Health Emergency of International Concern (PHEIC). Healthcare and public health systems had to integrate this novel threat into a host of regular functions, including, but not limited to, diagnostic, treatment and risk communication workstreams [1]. However, alongside the pandemic and its immediate impacts, misinformation around novel treatments and potential mitigation measures resulted in secondary health threats [2].

Public Health England (PHE) was alerted to changes in the patterns of poisoning at an international level through the World Health Organization INTOX Network of Poisons Centres. Early reports included cases of people self-medicating to prevent or treat COVID-19 and increased exposures to cleaning products, methanol and hand sanitisers (HSs). These reports prompted the Centre for Radiation, Chemical and Environmental Hazards (CRCE) at PHE to undertake enhanced domestic toxicovigilance and surveillance of poisoning outcomes, in conjunction with the UK National Poisons Information Service (NPIS).

Surveillance of public health risks broadly fall into two main categories: indicator-based surveillance and event-based surveillance. Indicator-based surveillance is the routine collection and analysis of structured data on known health risks [3,4]. In public health, these data often come from medical practices, laboratories and hospitals to alert health services to risks and outbreaks of endemic or seasonal infectious diseases. Event-based surveillance (EBS) looks to identify emerging or infrequent public health threats that sit outside standard reporting criteria and structures [5,6]. EBS is a functional component of the early warning and response process and encompasses the organised collection, monitoring, assessment and interpretation of mainly unstructured information (from formal and informal sources such as official news websites and social media) regarding incidents or hazards that may represent a risk to human health [3,5,6,7].

Collation of information and assessment of potential health risks to a population can be performed using local, national and international information sources [3]. Mapping health risks occurring abroad may provide valuable insights, particularly if a health event occurring overseas has the potential to cross borders [3]. However, for an EBS system to be effective, collated reports then require timely and sensitive processing [3,5]. Information gathered during EBS activities needs to be analysed for relevance in order to separate verified reports from rumours or duplicated information [3,6]. Early detection of information that could constitute a novel or rare event is key to a rapid response and mitigating the public health impact of said event [3]. As such, relevant information that could constitute a signal then needs to be communicated to decision makers to enable them to be reactive to dynamic health risks [3,5]. When a health event or risk is verified, downstream action plans should look at how it can be monitored through existing indicator-based health surveillance systems [3].

Established in 2014, the CRCE EBS strategy initially sought to capture information around chemical incidents in the European Union (EU). EBS was undertaken to support situational awareness and use of the web-based communication platform RASCHEM: a rapid alerting system for chemicals [8]. The RASCHEM system allowed regional poisons centres and public health authorities to communicate information on chemical incidents and poisonings that had potential cross-border implications. Accordingly, the EBS methodology targeted reports detailing chemical releases and adverse events including occupational exposures, product contamination, explosions and clusters of poisoning in Europe [8]. However, the EBS protocol was subsequently expanded to include reports from outside the EU and used to detected chemical incidents globally. International chemical incidents were recorded for situational awareness and to inform training sessions delivered in international settings [9].

From April 2020, CRCE expanded EBS activities to monitor how the COVID-19 pandemic may have affected the risk of chemical events, human poisoning and potential toxic exposures. This work involved analysis of toxicological issues reportedly associated with the COVID-19 pandemic in the international media. Domestic poisoning enquiry data collected by UK NPIS were also analysed, sometimes informed by patterns observed in media reports. For instance, if particular patterns of exposure were reported in international media and it was judged that similar exposures may occur in the UK, these were reported at toxicovigilance meetings held between CRCE and the UK NPIS.

Results of COVID-related toxicovigilance work were also shared with other government stakeholders, such as PHE colleagues working on the COVID-19 incident response. For instance, data were shared with teams who were monitoring social media for misinformation around potentially hazardous practices such as self-medication with novel treatments for COVID-19. In addition, reports were also shared with the WHO and international partners in order to inform real-time toxicovigilance and health protection activities. This article summarises the chemical health impacts of the COVID-19 pandemic as reported in the international media, through government announcements and in peer-reviewed literature over the study period.

## 2. Materials and Methods

In April 2020, CRCE started conducting pilot media searches to identify any reports of potential toxic exposures and poisonings associated with efforts to prevent or treat COVID-19. Evidence and articles that reported potential use of various chemical products that posed a risk to human health, the first of which dated back to January 2020, were collated on a database. Over April 2020, as the authors became more familiar with different themes that were arising through these manual searches, daily Google Alerts were set up to identify further news items of interest. By the end of April, the following ten Google Alerts were in place, and these stayed operational over the duration of the data collection period, to the 31st of March 2021:

‘clean* covid health’, ‘consumed covid hospital’, ‘drink* covid health’, ‘methanol covid’, ‘plant* toxic* covid’, ‘poison* covid’, ‘protect* covid kill’, ‘remedy covid’, ‘toxic* covid’ & ‘unproven covid’

Media reports targeted through this process demonstrated potential for changes in poisoning or potentially hazardous exposure trends as a direct or indirect result of COVID-19-related behaviours and policies. Other targeted media included government reports or announcements that issued warnings around particular chemical public health concerns (Table 1). As there were a number of international clinical trials undertaken to identify pharmaceutical preparations that were suitable for the treatment or prevention of COVID-19, articles on human pharmaceuticals were only included if there was some evidence of self-medication, black market sales, misinformation, safety concerns and/or continued clinical use despite established safety concerns.

Clickbait articles or fraudulent marketing that directly promoted the use of pharmaceuticals, chemicals or remedies were excluded, as articles needed to demonstrate that rumours around the product in question were salient in a community or posed a potential public health threat. Here, ‘clickbait’ refers to a type of online content designed to be sensationalist, attention-grabbing and/or misleading for the purpose of generating clicks and associated advertisement revenue. Clickbait is not typically published by traditional media outlets, and the content is rarely genuinely informative, especially in comparison to news reports written by professional journalists.

Reports indicating that the frequency and severity of COVID-19 infections appeared higher in areas of known chronic environmental exposure to toxic substances including air pollution and mining by-products are an important public health area for future research, though were outside the scope of this article [10].

Information was extracted from relevant articles. Extracted information included the chemical substance identified in the report, the subject country, geographic region (Supplementary Table S1), whether health risks were amplified by misinformation or endorsements from prominent figures, and whether the article reported actual exposure(s) or increased risk of exposure(s). As there was evidence that people acted on misinformation promoted by prominent public figures, promotion of any supposed preventative or treatment measure with an unreliable evidence base was recorded as misinformation. The scope of the media organisations that published the articles was also captured and categorised based on whether a report was published in ‘local media’, ‘national media’, by a ‘public broadcaster’, a ‘scientific media organisation’, in the ‘international media’ or if the article was ‘peer-reviewed’. However, the nature of international media meant that sometimes news reports were published in countries other than the subject country. For instance, if the British Broadcasting Corporation (BBC) (London, UK) published an article about poisonings in Bolivia, the subject country would be recorded as ‘Bolivia’ and the news organisation would be recorded as ‘public broadcaster’.

Where two articles reported the same information, only one article was entered into the database to avoid duplication of information. However, if two articles reported the same event, though one was published at a later date with unique follow-up information, both articles were recorded in the database. In the event that multiple media sources did report the same information, the highest-quality source was used. For example, when a media article discussed an official press release, government report or peer-reviewed journal article, the original information source was used wherever possible and the media report was excluded. Reports on poisonings and potentially hazardous exposures were corroborated across different media sources; however, it was not possible to verify information within the reports (see limitations).

The active ingredient and/or brand name associated with actual poisoning cases, as well as the severity of poisoning were also recorded where possible. International standards were used to assess poisoning severity score (PSS) as thoroughly as possible, where information was provided [12]. However, as the majority of sources assessed were media reports, the actual severity recorded should be interpreted with some caution. In the case of multiple people being poisoned with different clinical outcomes, the highest severity was recorded. For instance, in an event where multiple people were hospitalised and some died, the severity of the symptoms was reported as ‘fatalities’. In articles reporting that a person or people were reportedly hospitalised but no other information on symptoms was available, the severity of the poisoning case was assumed to be ‘severe’.

The nature of the poisoning event was also recorded where reported, such as whether poisoning resulted from ‘normal use at excess’ of a substance, ‘misuse’, ‘self-harm’, use of a ‘counterfeit, contaminated or unusually toxic preparation’, or whether the exposure was an ‘accident’ (e.g., a child accessing household chemicals). Where regulated chemicals were not used in accordance with manufacturer’s recommendations (such as mixing different cleaning products or taking a supratherapeutic dose of pharmaceuticals), the nature of the poisoning was marked as ‘misuse’. In rare cases identified where a highly toxic substance (including pharmaceuticals without suitable medical supervision) had been administered to a person with a COVID-19 infection and there was a resulting fatality, the case was recorded as a poisoning fatality even if it was unclear whether the person died as a result of COVID-19, the substance, or a combination of the two. Finally, while severe symptoms or fatality following ingestion of ethanol-based HS is possible, it is significantly less toxic than methanol-based HS [13]. Therefore, where fatalities have been reported following the ingesting HS but the form of constituent alcohol is not specified, the product was assumed to contain methanol.

## 3. Results

Over the course of the data collection period, spanning the 1st of April 2020 to the 31st of March 2021, a total of 329 relevant media reports and journal articles were collated. A brief summary of the results is included below (Table 2). Table 2 provides an overview of the chemical and pharmaceutical substances associated with potential toxic exposures and the COVID-19 pandemic, as well as the countries in which these substances were reported to be a health risk. Table 2 includes data from horizon scanning efforts, such as instances where there is evidence people are using a highly popularised herbal remedy as a cure for COVID-19, or where a government has issued warnings not to use a particular product to prevent or cure COVID-19, even if there are no reports to indicate that clinical poisoning has occurred as a result. For more detailed information on the substances and associated articles that inform Table 2, please refer to Supplementary Table S2, which provides a high-level summary of the context, poisonings and reports associated with each substance.

Poisonings and other potentially hazardous exposures reported internationally were influenced by a wide range of effects of the COVID-19 pandemic and associated policy. Table 3 provides a thematic overview of the different circumstances that resulted in actual toxic exposures and poisoning cases for each substance. The ‘misuse’ of a product that resulted in poisoning typically centred around chemicals used for cleaning (at both the household and commercial/industrial level), as well as pharmaceuticals. Poisonings resulting from an ‘accident’ included accidental exposures to household chemicals, HS and pharmaceuticals amongst children, which was sometimes associated with children spending more time at home under lockdown measures in the reports. Other accidental poisonings associated with COVID-19 lockdown measures in reports were snakebites, fungal exposures, accidental spraying of people with industrial chemicals and chemical incidents at industrial facilities closed or reopened due to lockdown measures. Poisonings associated with the ‘use of counterfeit, contaminated or unusually toxic preparations’ were typically centred around use of unusually toxic recreational drugs, bootleg alcohol (methanol), and ingestion of methanol-based HS. Poisoning resulting from herbal remedies, or a substance given to a person or promoted on social media as a COVID-19 remedy, was also considered poisoning as a result of a ‘counterfeit’ product. However, poisoning associated with chlorine dioxide/MMS products was classified as ‘misuse’ of a substance rather than a ‘counterfeit’ substance. This form of classification was used because there was evidence that chlorine dioxide was known to be a chemical intended for use in water treatment, and some people had purchased and consumed chlorine dioxide-based water purification products that advertised they were ‘not marketed for internal use’ [14]. As per Table 3, the most common circumstances involved in a toxic exposure were ‘misuse’ of a substance, followed by ‘accident/ chemical incident’, then ‘use of counterfeit, contaminated or unusually toxic preparation’ resulting in poisoning.

The numbers of articles where potential or confirmed poisoning(s) were reported, in addition to information that could be used to deduce poisoning severity, are displayed in Table 4. The one hundred and one (101) reports where poisoning severity was indicated were classified according to the presence of ‘minor symptoms’, ‘moderate/severe symptoms’ and ‘fatality/fatalities’. Moderate and severe poisonings were grouped together as a single category, as it was often not possible to deduce the precise severity with the amount of information provided. Furthermore, moderate poisoning symptoms can take on increased significance in a clinical setting if a patient experiences them over a prolonged period of time. However, it should be noted that the number of articles that discuss a particular form of poisoning is not an indication of the total number of people affected. For instance, the single report of a styrene gas leak in India indicated there were at least 13 fatalities and 1000 people hospitalised as a result of the chemical incident [15]. An accurate quantification of the number of people affected by each substance for each exposure or incident was not possible, as sometimes this was not provided or reporting dates between congruent sets of data overlapped and an accurate sum could not be feasibly deduced.

The results in Table 4 indicate that the severity of poisoning (where indicated) correlated with the number of relevant reports identified. Reports detailing a fatality or fatalities were the most common (61.39%), followed by reports describing ‘moderate /severe symptoms’ (35.64%) and then those describing ‘asymptomatic/ minor symptoms’ (2.97%). The six most frequently reported categories associated with actual poisoning cases were ‘bootleg or homebrewed alcohol’ (14.85%), ‘opioids’ (10.89%), ‘(hydroxy)chloroquine’ (9.90%), ‘self-harm with unknown substance’ (8.91%), ‘methanol-based hand sanitiser’ (8.91%) and ‘ethanol-based hand sanitiser’ (8.91%).

In order to assess the level of reporting associated with each region of the world and the form of media involved, information on the media source and subject country was captured. An analysis of the types of media that informed the results of this study and the number of reports by region is provided in Table 5. The results demonstrate that different regions have had different levels of representation in the results. The two countries that constitute the region of North America were the subject of 37.99% of reports collated over the research period. The next most reported on region, ‘Western Europe and Nordic Countries’, accounted for 11.55% of reports. These regions were followed by ‘Central and South Asia’ (11.25%), ‘Sub-Saharan Africa’ (9.42%), ‘Oceania’ (7.90%), ‘South America’ (6.08%), ‘East Asia’ (5.17%), ‘Multiple Regions’ (4.56%), ‘North Africa and the Middle East’ (2.13%), ‘Central America and the Caribbean’ (2.13%) and ‘Eastern Europe, South East Europe and South Caucasus’ (1.82%). There were also some notable differences in representation within regional categories, with all twenty-six reports from Oceania concerning Australia and/or New Zealand. Further, twenty-six of the thirty-seven reports from ‘Central and South Asia’ discussed poisonings and potential hazardous risks in India only.

National commercial media (28.57%) and international commercial media (28.27%) were the most significant sources of information included in the results. The categories ‘local media’ (15.20%) and ‘government report or press release’ (10.94%) were the next most significant media sources. All government announcements and national public broadcaster sources included in the results originated from countries where English is an official language: The United States, Canada, Australia, New Zealand, South Africa, the United Kingdom and Ireland. Poisonings and potentially hazardous exposures from countries where English is not an official language are likely underreported in the international media available in English, and it is reasonable to assume that they are underrepresented in this study.

## 4. Discussion

Although the types of poisonings and potentially hazardous exposures identified in this study were largely known public health issues prior to the COVID-19 pandemic, there is evidence that the frequency and nature of some of these exposures were impacted by the COVID-19 pandemic and associated policy. When COVID-19 was declared a PHEIC in January 2020, there was an immediate global effort to increase hygiene and sanitation measures to mitigate transmission. Disinfectant (typically sodium hypochlorite) was sprayed in public places in countries including Iran, China, South Korea, Iraq, Italy, Vietnam, Lebanon, Georgia, France, Spain, Russia and Brazil [16,17,18]. Reports on the decontamination of public areas escalated in April and May of 2020, when drones were used to spray disinfectant in Morocco [19] and the Indian city of Ahmedabad [20]. An official in southern Spain also issued a public apology after a local beach was sprayed with diluted bleach [21]. A video subsequently emerged of migrant workers in Bareilly, India, being sprayed with sodium hypochlorite that a local official claimed was meant to be used to clean buses [22]. As people heeded government messages to be vigilant of hygiene, there was a surge in the purchase and use of HS and household cleaning products (HCP). In Canada, Australia and the United States, distillers and other newly licenced manufacturers tried to increase HS production to meet the increased demand [23]. However, these businesses subsequently experienced their own supply issues: there were not enough typical HS containers available for them to bottle the sanitiser they were producing [24]. This resulted in HS being distributed in packaging including beer cans, gin bottles, wine bottles and baby food pouches [25,26,27,28]. There were a number of cases of atypical packaging being associated with accidental exposure. For instance, two cases of accidental exposure to HS in atypical packaging were detailed in media and government sources: a 13-year-old girl who accidently consumed HS packaged in a liquor bottle in the US, as well as an 18-month-old who was accidently given a food pouch containing HS in Canada [23,29]. In Australia, a recall notice was also issued after nine bottles of HS were accidently sold in gin bottles and labelled as gin [28]. Gaps in the market may have also facilitated a context in which more highly toxic methanol-based HS was produced and distributed [30,31]. In January 2021, the US Food and Drug Administration (US FDA) put a countrywide alert in place for HS originating from Mexico, after testing found a significant number of products being imported from Mexico contained methanol [32]. Ingestion of methanol-based HS resulted in 15 severe poisoning cases and four deaths in New Mexico and Arizona between the 1st of May and the 30th of June 2020 [33]. Deaths due to ingestion of methanol-based HS were also reported in Russia and India [34,35].

From the onset of the pandemic, global networks of poisons centres began to report increased exposures and poisonings due to HS and HCP. Some enquiries resembled exposures typically reported to poisons centres, such as unintentional paediatric poisonings or people experiencing side effects after mixing cleaning products together [36,37,38,39]. Unintentional household exposures to chemicals, pharmaceuticals and consumer products (particularly HS) in children had increased [40,41,42]. However, more severe paediatric exposures to HS in public places (including a school and hospital) were reported in Australia, the United Arab Emirates, the United Kingdom and France [43,44,45,46]. A peer-reviewed study from France found that that the frequency and severity of ocular HS exposures in children were likely, in part, driven by the height of public dispensers relative to the child, the viscosity of some preparations and also delays in finding a water source to flush the sanitiser from the eye [47]. There were also reports of people being injured after using highly concentrated methanol solutions to clean their home or face mask [48,49]. One US study published in June 2020 found that 19% of participants had attempted to disinfect food items with HCP, 18% had used HCP directly on their skin, 10% had misted themselves with HCP, 6% had inhaled HCP fumes and 4% had ingested or gargled HCP or soapy water in order to prevent COVID-19 infection [50]. Of those who had exposed themselves to HCP, one-quarter reported at least one adverse health outcome that they attributed to the exposure. Adverse health outcomes included irritation of the eyes, skin or throat, headaches, difficulty breathing and feelings of dizziness or nausea [50]. There was also evidence of intentional ingestion of HCP and HS, with one woman in Austria developing severe gastrointestinal symptoms after ingesting 10 mL of ethanol-based HS each day for three weeks in order to prevent COVID-19 [51].

The inappropriate use of HCP and HS was a small part of a wider phenomenon in which people self-medicated and treated themselves with substances in the belief it would prevent or cure COVID-19. Internationally, people started purchasing and consuming vitamins, supplements and herbal remedies touted to boost immunity and combat COVID-19 infections [52]. In Sri Lanka, thousands of people queued to purchase an herbal remedy that purportedly would give the consumer lifelong immunity to COVID-19 [53]. The herbal remedy gained some perceived legitimacy after it was reported that some politicians, including the Minister for Health, had consumed the herbal drink [53]. In Madagascar, President Rajoelina was reported to promote a government-made, artemisia-based herbal remedy that was distributed to the population [54]. Known as ‘Covid-Organics’, the remedy was later shipped to countries across Africa [55,56]. A video made by an herbalist in Afghanistan also spread on social media and resulted in hundreds of people queueing to purchase his alleged remedy, which the Afghan Ministry of Public Health found to contain opioids including morphine and codeine [57]. No clinical poisonings associated with the above products were reported in the media, but they were of significant public health concern given their potential toxicity. Three notable poisoning incidents associated with supplements and herbal remedies did emerge from India. These cases included an asymptomatic patient who was found to have a highly toxic serum levels of Vitamin D (348 ng/mL) [58], a patient who developed severe liver disease after ingesting an herbal remedy [59] and an incident in which ten people were hospitalised after preparing a remedy they saw promoted as a cure for COVID-19 on TikTok [60]. Alcoholic beverages were also directly or indirectly promoted as a preventative measure by some prominent public figures or community leaders in Belarus, Kenya and India. [61,62,63]. In Ghana, misinformation suggested a locally produced gin was effective in preventing COVID-19 infection [64].

There were a number of reports of increased intentional ingestion of bleaches and cleaning products to treat or prevent COVID-19 from the start of the data collection period [65,66]. However, media reported that poisons centres from Belgium and around the United States recorded a spike in calls following a press conference on the 23rd of April 2020, in which US President Donald Trump made some comments about the ability of disinfectant to inactivate the virus [67,68,69,70,71,72]. Trump turned to Dr Deborah Birx, the coordinator of the White House COVID-19 response, and appeared to enquire whether disinfectant administered to a person through ingestion or injection could inactivate the virus [67,68,69,70,71,72]. These comments were reported in the media and by poisons centres to be associated with a number of confirmed poisonings [67,68,69,70,71,72]. There were also reports of increased enquires to poisons centres, with people seeking information on the efficacy of disinfectant or bleach in treating COVID-19 [73]. However, exposures to bleach to prevent or treat COVID-19 were not limited to household preparations. There was evidence from a number of countries that people were ingesting a substance called ‘Miracle Mineral Solution’ (MMS): a product marketed as a cure for a number of ailments by the Genesis II Church of Health and Healing in Florida, which first came to the attention of the US FDA in 2010 [74]. MMS is composed of chlorine dioxide, which is typically found (at higher concentrations) in industrial bleaches or used for water treatment (in extremely low concentrations). There is evidence to suggest that when MMS started to be marketed as a cure for COVID-19 by some individuals associated with the church, their revenue increased fourfold [75]. In addition to sales in the United States, MMS was marketed as a cure for COVID-19 in Australia, New Zealand and the United Kingdom [76,77,78]. MMS was also marketed and sold in Latin America by the Genesis II Church and others [79,80]. The Bolivian opposition even passed a bill to ensure supply of MMS in the public healthcare system, which was vetoed by the interim President, Jeanine Áñez [80,81,82,83]. In August 2020, the Pan-American Health Organization (PAHO) released a statement urging populations of the Americas and Caribbean not to consume MMS or chlorine dioxide products for the purpose of preventing or treating COVID-19 [84].

Publicity around pharmaceutical preparations being investigated for clinical efficacy in the fight against COVID-19 led to similar forms of self-medication and treatment. On the 19th of March 2020, President Trump promoted hydroxychloroquine, an antimalarial drug, as a ‘game changer’ in the fight against COVID-19 at a press conference [85]. Media reports of people attempting to purchase, being prescribed and self-medicating with the drug started emerging internationally, including some reports of associated overdoses and fatalities [86,87,88,89,90,91]. Use in some countries continued after clinical trials of the drug were cancelled in France, Brazil and Sweden for safety concerns [92,93]. Similarly, a study from Australia showing ivermectin inactivated COVID-19 in vitro led to widespread misinterpretation [94]. Ivermectin was incorporated into clinical guidelines across Latin America and people began self-medicating with the pharmaceutical [95,96]. Researchers in Peru noted that they were having trouble finding research participants who were not already taking the drug [96]. When human preparations of the drug became expensive and scarce, people turned to veterinary preparations [95]. Similar misinformation around ivermectin then spread in South Africa, where ivermectin was not authorised for human use [97]. However, the government eventually introduced a compassionate use clause in the face of black-market sales and concerns people were purchasing veterinary ivermectin to prevent or cure COVID-19 [97,98,99,100]. From February 2021, the US FDA also warned citizens that ivermectin, particularly veterinary ivermectin, was not suitable for use to treat COVID-19 after they learned of a case where someone had taken a dose of horse ivermectin [101,102]. Widespread administration of both hydroxychloroquine and ivermectin in Brazil, which were promoted by President Bolsonaro, and colloquially referred to as a ‘COVID kit’ (alongside other pharmaceuticals), were reportedly linked to at least three deaths and five liver transplants [103,104].

A separate EBS study undertaken by PHE found that between November 2014 and June 2020, methanol was involved in 86 chemical incidents and was associated with 2090 fatalities internationally [9]. The majority of these incidents occurred due to the substitution of ethanol for methanol in alcoholic beverages. However, there is evidence to indicate that the COVID-19 pandemic may have led to increased exposures through methanol-based alcoholic drinks. For instance, misinformation about alcohol that started circulating in Iran was, in part, influenced by an anecdotal report of an individual in Wuhan who drank hot toddies (a hot whiskey drink) to ease his COVID-19 symptoms. The story was initially broadcast in the UK and US press, and then reportedly circulated in Farsi on Iranian social media, alongside government messages about hygiene [105,106,107]. Misinformation started to spread that suggested that high-proof alcohol could prevent a COVID-19 infection, and this led to Iranians attempting to procure alcohol in a country where consumption is banned and not a cultural norm [107]. Many people were limited to purchasing illicit, methanol-based sources of alcohol, and at least 700 fatalities and 3100 hospitalisations were reported [108]. There was also some evidence from Iraq, Nepal and India (in particular), that lost earnings due to COVID-19, fear of job losses, fear of contracting COVID-19 and one case of COVID-related social ostracism contributed to self-harm and suicides by poison [109,110,111,112,113,114].

Border closures, lockdown prohibition measures and lost earnings associated with the pandemic were also associated with a variety of poisonings resulting from use of unusually toxic drugs and beverages. There is evidence to suggest border closures impacted typical illicit drug supplies in Canada, and highly toxic opioid preparations containing fentanyl started circulating, resulting in a record number of deaths [115]. During the study period, even non-opioid drugs including cocaine and methamphetamine were found to contain fentanyl and were attributed to one death in Peterborough, Canada [116]. In India, South Africa and Mexico, lockdown measures sometimes included bans on the sale and purchase of alcohol. In India, this resulted in at least seven deaths from people consuming aftershave or HS while suffering from alcohol withdrawal between March and May 2020 [117]. In South Africa, where the alcohol ban was introduced to reduce the burden of alcohol-related injuries on the healthcare system, there were at least eleven deaths resulting from people consuming methanol-based, bootleg alcohol products between the 23rd of March and the 31st of May 2020 [118,119,120]. In Mexico, alcohol production was classed as a nonessential activity under lockdown measures, with some states also banning or placing limitations on the purchase of alcohol [121,122]. Reduced alcohol supplies and economic impacts of the pandemic reportedly resulted in people buying cheaper alternatives and bootleg products. By mid-May, at least one hundred people had died from consumption of highly toxic alcohol in Mexico, with another eighteen deaths reported in June 2020 [123]. Reports from Bangladesh, Turkey and Cameroon also suggested that loss of income associated with the COVID-19 pandemic reduced the purchasing power of consumers, who sometimes turned to lower-quality alcohol products, which were more affordable [124,125,126].

Lockdown measures affected a number of other behaviours, business operations and council functions, which increased risk of poisoning or potential hazardous exposures. Media reports and research suggested that the COVID-19 pandemic and associated policy measures were changing regular and problem drinking patterns in Australia, Canada, New Zealand, the UK, USA and Belgium [127,128,129,130,131]. Data from the United Kingdom showed some people were actually drinking less due to lockdown measures, but that people with a history of abuse, under high levels of stress and who were already heavy drinkers were more likely to have increased their drinking or relapsed into problem drinking [132,133]. Substance abuse and liver experts in the UK and US warned that previously recovered high-risk drinkers were being admitted to hospital with health issues [134,135,136]. Alcohol-related deaths in the UK were reported to be 16% higher in January to September of 2020 than they were in the same period of 2019 [137]. Under social distancing measures, states across the Southern US also reported an increase in snake bite cases, which experts attributed to more time being spent in home gardens and in the outdoors [138,139,140,141]. Increased interest in mushroom-foraging activities during lockdown was also reported in Australia, Canada, the US and France, with warnings that it may result in or had resulted in additional poison centre calls [142,143,144,145]. Scottish councils and local organisations also put out warnings that their ability to clear giant hogweed, a plant that can cause severe burns and blindness, had been hampered by lockdown measures [146]. In the US, COVID-19-related funding cuts to local environmental groups were also cited as a reason they could not undertake previous efforts to display signs and warn locals of toxic agal blooms at recreational water sites [147]. As industrial sites were unattended or attempted to reopen after a prolonged closure under lockdown measures, there were at least two resulting chemical incidents in India, including a styrene gas leak that killed 13 people and resulted in another 1000 being hospitalised [15,148].

Results collected over the course of this study indicate that the relative representation of different countries and poisonings is associated with the official languages of the country and poisoning severity. Most articles that detailed the severity of actual poisonings were incidents in which a fatality or multiple fatalities occurred (61.39%). It is reasonable to assume that COVID-19-related toxic exposures and poisoning cases that only resulted in mild or moderate symptoms were not reported in the media and are underrepresented in this study. In addition, articles from countries where English is an official language were overrepresented in the data. For instance, opioid exposures in Canada were one of the top six most frequently reported issues captured by this study. In addition to English being an official language in Canada, the country also has a highly developed healthcare system where toxicovigilance efforts have been strengthened over recent years [149]. COVID-19-related poisoning risks in resource-poor settings may have been less likely to have been reported if poisonings were not easily diagnosed by physicians without specialist training in clinical toxicology, or if associated healthcare systems did not have the necessary laboratory capacity to confirm the cause of poisoning. Hence, it is possible that the true burden of COVID-19-related toxicological issues in low- and middle-income countries may not have been reflected in the government or media sources collated through this study. For instance, despite a number of articles detailing poisonings in the USA resulting from methanol-based HS imported from Mexico, this study did not find a single article in English that indicated comparable exposures in Mexico.

Toxicovigilance data and potential health risks captured over the study period were routinely summarised into reports and briefings shared within PHE as part of the national incident response, as well as with the UK NPIS. The NPIS shared anecdotal observations regarding perceived changes to the nature and frequency of poisoning calls they received, which PHE would compare to the international literature and media reports. If international poisoning patterns were seen as possible in the UK, the NPIS would also take reports gathered by PHE and analyse call data to see if similar trends were occurring in the United Kingdom. For instance, when PHE forwarded reports regarding ocular injuries in children associated with HS in France, the NPIS queried their own data. The NPIS did not find evidence of equivalent exposures in their records, but given the evidence provided, went on to update advice published on the clinical toxicology database, TOXBASE. In the UK, TOXBASE is utilised by healthcare professionals including doctors, telehealth services (NHS 111) and the emergency services. Information gathered over the study period was also shared with partners across the UK government to teams undertaking related work, such as those that monitor misinformation on social media. Reports were also shared with the WHO and with public health colleagues in Ethiopia and Myanmar so they were aware of risks that could occur in their respective countries. It is the authors’ hope that over time, the themes and issues presented in this study and by the media are examined by poisons centres and health officials in other countries in order to produce robust data and studies in the peer-reviewed literature.

## 5. Conclusions

This article provides a thematic overview of 329 international media reports, government announcements and peer-reviewed articles that detailed poisoning cases or potential hazardous exposures associated with impacts of the COVID-19 pandemic. Poisoning risks identified over the course of the data collection period, spanning April 2020 to March 2021, included increased exposure to household cleaning products and hand sanitiser, self-medication with pharmaceuticals, use of herbal remedies and traditional medicine, changes to alcohol drinking patterns under lockdown measures and use of contaminated or unusually toxic products. Loss of income may have been a factor in some of these issues, driving consumers to lower-quality alcohol products and resulting in self-harm. Misinformation was also a major driver of poisoning risks and exposures, resulting in self-medication with a variety of substances, including pharmaceuticals and water treatment chemicals clearly marked as unsuitable for internal use. The pandemic and associated public policy also resulted in a number of indirect effects, increasing exposure risks in the natural environment and preventing routine treatment for other poisoning risks. The content of this research informed toxicovigilance functions in the United Kingdom as part of the national COVID-19 public health response and contributed to evidence to support public health interventions. Further research needs to be undertaken internationally to examine poisoning risks reflected in formal healthcare data and to capture the full health impacts of the pandemic. It is of concern that the spread of misinformation during the COVID-19 pandemic led to a number of toxicological issues, especially through the use of ‘novel’ treatments for the prophylaxis/treatment of COVID-19. It is important that public health agencies work closely with poisons centres to ensure that authoritative advice is communicated to the public to prevent or limit the impact of toxicological issues.

### Limitations

Effort has gone into ensuring that the information provided in this article is of a high quality, with cross-verification of some reports undertaken where possible. However, no formal fact checking was undertaken to independently verify the information contained within the media reports that inform this article. In addition, the health risks discussed in this article and their geographic distribution should be interpreted with some caution, as some poisonings or potentially hazardous exposures may have been underreported, particularly in countries where English is not an official language. Accordingly, the results of this study should not be considered an exhaustive list of all toxic exposures and poisonings associated with the COVID-19 pandemic.

## Figures and Tables

**Table 1:** Inclusion and exclusion criteria.

Criteria	Included	Excluded
Data Sources	Peer-reviewed literature, grey literature, government reports or press releases, health service reports or press releases, and news reports. Reports must detail a chemical incident, potential health risks to the public, the promotion of unverified treatments by prominent public figures, or widespread promotion of an unverified treatment on social media, which could result in toxic exposures. The WHO defines chemical incidents as the ‘uncontrolled release of a toxic substance, potentially resulting in harm to public health and the environment’ [11]. The public health threat and poisonings must be linked in the media to impacts of the COVID-19 pandemic, related behaviours and/or associated public policy.	Clickbait news articles or fraudulent marking that promoted the use of particular substances to prevent or treat COVID-19. Articles that detailed a chemical event that could not be verified by a second source.
Health Risks	Reported chemical events, poisonings and toxic exposures linked to the COVID-19 pandemic, as well as reported trends or misinformation that could result in chemical events, human exposures and poisoning. Human exposure to an unknown substance used to disinfect public spaces, or to test, treat or prevent COVID-19. Pharmaceuticals with established safety concerns when used to treat COVID-19, particularly if there is evidence of self-medication.	Radiological and biological health impacts or risks resulting from the COVID-19 pandemic. Environmental risks resulting from the COVID-19 pandemic that were not chemical in origin and were unlikely to impact human health. Chemical impacts and risks resulting from the COVID-19 pandemic that were not associated with potential human health impacts.
Scope	International, national and local reports published in the English language from the 28th of January 2020 to the 31st of March 2021.	Reports published outside these dates and/or in a language other than English.on

**Table 2:** Substances identified in poisoning cases or as a poisoning risk associated with COVID-19, as well as countries where this risk has been identified (more detailed results and associated references can be found in Supplementary Table S2, which provides a high-level summary of the context, poisonings and reports associated with each substance).

Group	Substances	Countries
Agrochemicals	Pesticides general	United States
	Silvadur 930	United States
	Glyphosate	United Kingdom
Biocides	Rat poison	Thailand
Consumer Products	After shave	India
	Hand sanitiser	Austria, Australia, Canada, France, India, United Arab Emirates, United Kingdom, United States
	Methanol-based hand sanitiser	India, Ireland, Russia, United Kingdom, United States
	Surge production of hand sanitiser/hand sanitiser in atypical packaging	Australia, Canada, United States
	Insect repellent (citriodiol)	United Kingdom
Household Chemicals	Cleaning supplies (including household disinfectant and bleach)	Belgium, Canada, United States
	Fish tank cleaner (chloroquine phosphate)	United States
	Pool/hot tub chemicals	Canada
	Kerosene/kerosene combustion by-products	Kenya
Industrial/Commercial Chemicals	Disinfectant used in public spaces (typically sodium hypochlorite, chlorine-based cleaners or bleach)	Brazil, Canada, China, France, Georgia, India, Iran, Iraq, Italy, Lebanon, Morocco, Nigeria, The Philippines, Russia, South Korea, Spain, United Kingdom, Vietnam
	Disinfectant tunnels (typically sodium hypochlorite)	Bosnia and Herzegovina, Chile, China, Mexico, Singapore, Sri Lanka, Pakistan, Vietnam
	Chlorine dioxide (Magic Mineral Solution (MMS))	Argentina, Australia, Bolivia, Costa Rica, France, Mexico, New Zealand, United States
	Sodium chlorite (often sold alongside hydrochloric acid to produce chlorine dioxide/ MMS)	Costa Rica, New Zealand, Spain, United Kingdom
	Styrene gas	India
	HDQ Neutral (ammonia-based disinfectant)	United States
	Mixture of nitric oxide, sodium nitrate and sodium hydrate	India
	Gasoline	The Philippines
	Ethylene Oxide	United States
	Graphene	Canada
	Silver and titanium oxide nanoparticles	Belgium
	Unspecified toxic gas	India
Recreational drugs/Drugs of Misuse	General	Belgium
	Cocaine	France
	Opioids	Afghanistan, Canada, United States
	Cannabis	Canada
Dietary Supplements and over-the-counter medicines	Colloidal silver	United States
	Vitamins	India, United States
	General	United States
Herbal, Homeopathic or Traditional medicine	Traditional Chinese medicine	Italy, Iran, China
	Madagascan remedy	Central African Republic, Democratic Republic of Congo, Equatorial Guinea, Guinea-Bissau, Liberia], Madagascar, New Zealand, Tanzania
	Other herbal remedies	Afghanistan, Cameroon, India, Nigeria, Sri Lanka, Tanzania, Venezuela, Zimbabwe
	Oleander	United States
	Essential oils	Australia, United States
	Ephedra	Australia
Animal, plants and fungi	Snake bite	United States
	Mushroom foraging	Australia, Canada, France, United States
	Giant hogweed	United Kingdom
	Datura stramonium seeds	India
	Quina plants	Brazil
	Algal blooms	United States
	Liquorice root	Turkmenistan
	Plants general	United States
Heavy Metals	Lead	United States
Human pharmaceuticals	(Hydroxy)chloroquine	Australia, Brazil, Cameroon, Democratic Republic of Congo, India, Indonesia, France, Niger, Nigeria, Pakistan, Sweden, United States
	Ivermectin	Australia, Bolivia, Brazil, France, Guatemala, Ireland, Jamaica, New Zealand, Peru, United States, South Africa, South Korea
	Counterfeit pharmaceuticals	Cameroon, Democratic Republic of Congo, Niger
	Prescription opioids	United States
	Famotidine/antacid	United States
	Avigan/ favipiravir	Japan
Veterinary pharmaceuticals	Ivermectin	Australia, Peru, South Africa, United States
	Unspecified cattle deworming medication	South Africa
Fraudulent tests and treatments	‘Virus Blocker’ or ‘Air Doctor’ (chlorine dioxide)	Australia, Bolivia, Russia, South Sudan, United States
	Eucalyptus-based virus blocker lanyard	Indonesia
	Fake tests and treatments (general)	Myanmar, United States, United Kingdom
Alcohols	Methanol	North Korea, South Korea, Spain
	Ethanol-based drinks	Australia, Belarus, Belgium, Canada, Ghana, India, Kenya, New Zealand, United Kingdom, United States
	Bootleg, low-quality or home-brewed alcohol (often containing methanol)	Cameroon, Iran, Mexico, South Africa, Turkey
Unspecified	Self-harm with unknown substance	India, Iraq, Nepal
	Alleged criminal activity and murder with unknown substance	India, Kenya

**Table 3:** Mode of exposure or poisoning.

Substance	Normal Use (Sometimes at Excess) Resulting in Poisoning	Misuse Resulting in Poisoning	Misuse as a Result of Self-Harm Resulting in Poisoning	Use of Counterfeit, Contaminated or Unusually Toxic Preparation Resulting in Poisoning	Accident/ Chemical Incident	Poisoning Treatment Delayed by Disruption to Normal Services	Unclear
Rat poison			x				
After shave		x					
Ethanol-based hand Sanitiser					x		
Methanol-based hand sanitiser		x		x			
Household cleaning supplies (including household disinfectant and bleach)	x	x			x		x
Fish tank cleaner		x					
Pool/hot tub chemicals					x		
Disinfectant used in public spaces (often sodium hypochlorite, chlorine-based cleaners or bleach)		x					
Chlorine dioxide (MMS)		x					
Sodium chlorite (often sold alongside hydrochloric acid to produce chlorine dioxide/MMS)		x					
Styrene gas					x		
HDQ Neutral (ammonia-based disinfectant)		x					
Mixture of nitric oxide, sodium nitrate and sodium hydrate		x					
Unspecified toxic gas					x		
Opioid drugs				x			
Cannabis					x		
Vitamins	x						
Herbal remedies				x			
Snake bite					x		
Mushroom foraging					x		
Datura stramonium seeds				x			
Plants general					x		
Lead						x	
(Hydroxy)chloroquine		x					
Human ivermectin		x					
Prescription opioids			x		x		
Veterinary ivermectin		x					
Unspecified cattle deworming medication		x					
Methanol		x					
Ethanol-based drinks	x						
Bootleg or home-brewed alcohol (often containing methanol)				x			
Self-harm with unknown substance			x			x	
Alleged poisoning of others or other criminal activity with unknown substance							x

**Table 4:** Severity of actual poisonings in reports where case symptoms and/or severity are indicated (*n* = 101).

Substance	Asymptomatic/Minor Symptoms	Moderate/Severe Symptoms	Fatality/Fatalities	Total
Rat poison		1		1
After shave			1	1
Ethanol-based hand sanitiser	1	8		9
Methanol-based hand sanitiser			9	9
Household cleaning supplies (including household disinfectant and bleach)	1	4		5
Fish tank cleaner			1	1
Pool/hot tub chemicals		1		1
Disinfectant used in public spaces (often sodium hypochlorite, chlorine-based cleaners or bleach)			1	1
Chlorine dioxide (MMS)			3	3
Sodium chlorite (often sold alongside hydrochloric acid to produce chlorine dioxide/ MMS)		1		1
Styrene gas			1	1
HDQ Neutral (ammonia-based disinfectant)		1		1
Mixture of nitric oxide, sodium nitrate and sodium hydrate			1	1
Unspecified toxic gas		1		1
Opioids			11	11
Vitamins	1			1
Other herbal remedies		1		1
Snake bite		1		1
Mushroom foraging		1		1
Datura stramonium seeds		1		1
(Hydroxy)chloroquine		3	7	10
Hydroxychloroquine or ivermectin (does not say which specifically)			1	1
Prescription opioids		1		1
Veterinary ivermectin		2		2
Unspecified cattle deworming medication		1		1
Methanol		2		2
Ethanol-based drinks		4	1	5
Bootleg, low-quality or home-brewed alcohol (often containing methanol)			15	15
Self-harm with unknown substance			9	9
Alleged poisoning of others or other criminal activity with unknown substance		2	1	3
Total	3	36	62	101

**Table 5:** News sources and regions (*n* = 329).

Source	Sub-Saharan Africa	North Africa and Middle East	Central and South Asia	Eastern Europe, South East Europe and South Caucasus	Western Europe and Nordic Countries	East Asia	Oceania	South America	Central America and the Caribbean	North America	Multiple Regions	Total
Journal Article		1			4	1	1	1		5		13
Academic or Scientific News Publication *	1		1		2		1	2		2	3	12
Government Report or Press Release	1				5		3			25	2	36
International Commercial Media	14	3	10	3	7	12	4	12	3	20	5	93
Public Broadcaster	2		1		5		8	2		7	2	27
National Commercial Media	13	2	21	2	11	3	6	2	3	30	1	94
Local Media		1	4	1	4		3	1	1	34	1	50
Other/Unknown						1				2	1	4
Total	31	7	37	6	38	17	26	20	7	125	15	329

* Category includes news and opinion pieces published by outlets such as National Geographic or The Conversation.

## Data Availability

Not applicable.

## Supplementary Materials

The following are available online at https://www.mdpi.com/article/10.3390/ijerph182111133/s1, Table S1: Regional Classifications Used during Data Processing, Table S2: High-level Summary of Potential COVID-related Poisoning Risks Identified Over the Course of the Study Period. References [14,15,16,17,18,19,20,21,22,23,25,26,27,28,29,30,31,32,33,34,35,36,37,38,39,40,41,42,43,44,45,46,47,48,49,50,51,52,53,54,55,56,57,58,59,60,61,62,63,64,65,66,67,68,69,70,71,72,73,74,75,76,77,78,79,80,81,82,83,84,85,86,87,88,89,90,91,92,93,94,95,96,97,98,99,100,101,102,103,104,105,106,107,108,109,110,111,112,113,114,115,116,117,118,119,120,121,122,123,124,125,126,127,128,129,130,131,132,133,134,135,136,137,138,139,140,141,142,143,144,145,146,147,148,150,151,152,153,154,155,156,157,158,159,160,161,162,163,164,165,166,167,168,169,170,171,172,173,174,175,176,177,178,179,180,181,182,183,184,185,186,187,188,189,190,191,192,193,194,195,196,197,198,199,200,201,202,203,204,205,206,207,208,209,210,211,212,213,214,215,216,217,218,219,220,221,222,223,224,225,226,227,228,229,230,231,232,233,234,235,236,237,238,239,240,241,242,243,244,245,246,247,248,249,250,251,252,253,254,255,256,257,258,259,260,261,262,263,264,265,266,267,268,269,270,271,272,273,274,275,276,277,278,279,280,281,282,283,284,285,286,287,288,289,290,291,292,293,294,295,296,297,298,299,300,301,302,303,304,305,306,307,308,309,310,311,312,313,314,315,316,317,318,319,320,321,322,323,324,325,326,327,328,329,330,331,332,333,334,335,336,337,338,339,340,341,342,343,344,345,346] are cited in the supplementary materials.
