# Supplementary material for: Event-Based Surveillance of Poisonings and Potentially Hazardous Exposures over 12 Months of the COVID-19 Pandemic
Source: Int J Environ Res Public Health. 2021 Oct 22;18(21):11133. doi: 10.3390/ijerph182111133 (PMC8583514; doi:10.3390/ijerph182111133)
Supplement: Supplementary file 1 [file ijerph-18-11133-s001.zip › ijerph-1410377-Supplementary File 1.pdf]

International Journal of
Environmental Research and Public Health

MDPI

Table S1. Regional Classifications Used During Data Processing.

Region	Countries
Sub-Saharan Africa	Angola, Benin, Botswana, Burkina Faso, Burundi, Cameroon, Cape Verde, Central African Republic, Chad, Comoros, Congo-Brazzaville, Congo-Kinshasa, Djibouti, Equatorial Guinea, Eritrea, Eswatini, Ethiopia, Gabon, Gambia, Ghana, Guinea, Guinea-Bissau, Kenya, Lesotho, Liberia, Madagascar, Malawi, Mali, Mauritania, Mauritius, Mozambique, Namibia, Niger, Nigeria, Côte d'Ivoire, Rwanda, Sao Tome and Principe, Senegal, Seychelles, Sierra Leone, Somalia, South Africa, South Sudan, Sudan, Tanzania, Togo, Uganda, Zambia, Zimbabwe
North Africa and the Middle East	Algeria, Bahrain, Egypt, Iran, Iraq, Israel, Jordan, Kuwait, Lebanon, Libya, Morocco, Oman, Qatar, Saudi Arabia, Syria, Tunisia, United Arab Emirates, West Bank and Gaza, Yemen
Central and South Asia	Afghanistan, Bangladesh, Bhutan, India, Kazakhstan, Kyrgyzstan, Nepal, Maldives, Pakistan, Sri Lanka, Tajikistan, Turkmenistan, Uzbekistan
East Asia	Brunei, Cambodia, China, Indonesia, Japan, Laos, Malaysia, Mongolia, Myanmar, North Korea, The Philippines, Singapore, South Korea, Thailand, Timor-Leste, Vietnam, Hong Kong, Taiwan
Oceania	Australia, Cook Islands, Fiji, Kiribati, Marshal Islands, Micronesia, Nauru, New Caledonia, New Zealand, Palau, Papua New Guinea, Samoa, Solomon Islands, Tonga, Tuvalu, Vanuatu, West Papua
Eastern Europe, South East Europe and South Caucasus	Albania, Armenia, Azerbaijan, Belarus, Bosnia and Herzegovina, Bulgaria, Croatia, Cyprus, Czechia, Estonia, Georgia, Greece, Kosovo, Latvia, Lithuania, Malta, Moldova, Montenegro, North Macedonia, Romania, Russia, Serbia, Slovakia, Slovenia, Turkey, Ukraine
Western Europe and Nordic Countries	Austria, Belgium, Denmark, Finland, France, Germany, Hungary, Iceland, Ireland, Italy, Liechtenstein, Luxembourg, Monaco, The Netherlands, Norway, Portugal, San Marino, Spain, Sweden, Switzerland, United Kingdom
North America	Canada, United States of America
Central America and the Caribbean	Antigua and Barbuda, Bahamas, Barbados, Belize, Costa Rica, Cuba, Dominica, Dominican Republic, El Salvador, Grenada, Guatemala, Haiti, Honduras, Jamaica, Mexico, Nicaragua, Panama, Puerto Rico, Saint Kitts and Nevis, Saint Lucia, Saint Vincent and the Grenadines, Trinidad and Tobago
South America	Argentina, Bolivia, Brazil, Chile, Colombia, Ecuador, French Guiana, Guyana, Paraguay, Peru, Suriname, Uruguay, Venezuela
