# Supplementary material for: Event-Based Surveillance of Poisonings and Potentially Hazardous Exposures over 12 Months of the COVID-19 Pandemic
Source: Int J Environ Res Public Health. 2021 Oct 22;18(21):11133. doi: 10.3390/ijerph182111133 (PMC8583514; doi:10.3390/ijerph182111133)
Supplement: Supplementary file 1 [file ijerph-18-11133-s001.zip › ijerph-1410377-Supplementary File 2.pdf]

International Journal of
Environmental Research and Public Health

MDPI

Summary of Reports by Substance

A high-level summary of the context, poisonings and reports associated with each substance identified as a COVID-related poisoning risk is detailed in Table 1 below. In addition to the articles discussing potential risk for or actual poisonings (n = 329), this supplementary file contains some additional references (N = 2) in order to provide context around events such as press conferences and clinical trials.

Table S2. High-level Summary of Potential COVID-related Poisoning Risks Identified Over the Course of the Study Period

Group	Substance	Summary
Agrochemicals	Pesticides general	In April 2020, the Arizona Poisons & Drug Information Centre reported that as a result of social distancing measures there was an increase in exposures to chemicals around the home, including pesticides [1]. The same month, a researcher based in Geneva published concerns around the global availability of personal protective equipment (PPE) as a result of the COVID-19 pandemic and the risk this might pose to agricultural laborers [2].
	Silvadur 930	In May 2020, the state government of Tennessee distributed hundreds of thousands of masks to state residents. However, distribution was halted when it was reported that the masks had been treated with Silvadur 930, a registered pesticide [3]
	Glyphosate	A glyphosate-based pesticide, known by the commercial name 'Roundup', has been banned in a number of European countries including Scotland. In 2020, some Scottish council officials lobbied elected representatives to lift the ban on the herbicide as a number of complaints had been received about overgrown weeds posing a hazard to members of the public. The Scottish council of Midlothian lifted the restrictions on glyphosate use during the COVID-19 pandemic, but one news report suggested inadvertent spraying of private gardens and areas of wildflower growth may have also occurred [4].
Biocides	Rat Poison	In April 2020, a Thai woman ingested rat poison outside Thailand's Finance Ministry, citing the slow rollout of COVID-19 financial aid as her reason for the act [5].
Consumer Products	After shave	In May 2020, an article published in India reported that there were approximately 338 reported suicides recorded between the 19th of March and 2nd of May associated with national lockdown measures, which included a ban on producing and selling drinking alcohol. The report indicated that at least seven people had died as a result of consuming hand sanitiser or aftershave while suffering from substance withdrawal [6].
	Hand Sanitiser	From March 2020, poisons centres around the world started reporting increased exposures and poisonings resulting from hand sanitisers. In the United States, state health authorities and poisons centres in Pennsylvania [7], Washington State [8], West Texas [9] and Michigan [10] reported an increase in hand sanitiser-related exposures

Consumer Products cont.	Hand Sanitiser cont.	between March and May of 2020. One report published by the U.S. Centers for Disease Control in April found that the number of calls to poisons centres regarding hand sanitiser in March of 2020 were 36.7% higher than in 2019 [11]. Another report published by the American Association of Poisons Centers published in May 2020 concluded that hand sanitiser exposures nation-wide were 42% higher in March 2020 and 35% higher in April 2020 than for the same months in 2019 [12]. This report also found that nearly two thirds of hand sanitiser exposures for the reporting period (January 1 to May 10, 2020) were in children under 5 years of age [12]. Reports suggested that children in the US were either accidentally ingesting the hand sanitiser or getting hand sanitiser in their eyes [7,11].<br><br>Health Canada reported a 103% increase in calls to poisons centres regarding hand sanitisers, disinfectants and bleach in March 2020 comparted to the same period in 2019 [13]. Health Canada indicated that accidental exposures experienced by children were a particular concern [13]. In Australia, New South Wales (NSW) Health also reported that the state was experiencing an increase in calls regarding hand sanitiser exposure in the home in April, particularly among children and infants [14]. However, a case report from Austria showed adults were also at risk. A 41-year-old woman had presented at hospital suffering from vomiting and abdominal pain for two days, as well as hematemesis for several hours [15]. Doctors ascertained that the woman had been ingesting 10ml of ethanol-based hand sanitiser each day for at least three weeks in order to prevent COVID-19 infection. An endoscopy procedure revealed 'superficial mucosal damage in the oesophagus, slightly increased mucosal redness in the stomach and severely injured small bowel mucosa with extensive fibrinous exudates'[15].<br><br>Between June and August of 2020, a number of other cases involving individual children from various countries were reported in the media or in peer-reviewed articles. These reports included two Australian girls aged 5 and 6 years old being hospitalised after ingesting hand sanitiser in the home [16] and at school [17]. In the case of the 6 year old who ingested hand sanitiser in the home, her blood ethanol reading was found to be 41.1 mmol/L or 0.19%, which was nearly four times the NSW legal limits for driving under the influence of alcohol [16]. In the Unites Arab Emirates (UAE), a 6-year-old boy was blinded in one eye after getting hand sanitiser from a public dispenser in a hospital [18]. A similar incident was also reported in the United Kingdom, where a 3-year-old boy may have been partially blinded after using a public dispenser at a local attraction [19]. In August 2020, French Poison Control Centres reported that similar ocular exposures were occurring among children France. Cases involved children accidently introducing hand sanitiser to their eyes after using public dispensers, with some serious cases reportedly requiring surgery [20]. In January 2021, French researchers published a paper showing that ocular exposures of alcohol-based hand sanitiser in France between April and August of 2020 were seven times

Consumer Products cont.	Hand Sanitiser cont.	higher than over the same period in 2019 [21]. One paediatric ophthalmology centre included in the study found 13% of patients had severe outcomes that required surgery. The study also suggested that the height of public sanitiser dispensers, viscosity of some sanitisers and delays in finding a suitable water source to flush sanitiser from the eye likely contributed to the frequency and severity of outcomes [21].<br><br>Between September 2020 and March 2021, a number of other organisations and poisons centres also reported increased cases and calls regarding ethanol-based hand sanitiser, a significant number of which were regarding exposures in children and adolescents. These organisations included the South Texas Poisons Center [22], the Upstate New York Poison Center [23], the Canadian Association of Poison Control Centres [24], Alberta Poison and Drug Information Service [25] and the Banner Poison Center in Arizona [26].
	Hand Sanitiser containing methanol	In May 2020, an article published in India indicated that there were approximately 338 reported suicides recorded between the 19th of March and 2nd of May associated with national lockdown measures, which included a ban on producing and selling drinking alcohol. The report indicated that at least seven people had died as a result of consuming hand sanitiser or aftershave while suffering from substance withdrawal [6]. In June, the Central Bureau of Investigation in India had alerted domestic law enforcement agencies that increased demand for hand sanitiser may result in counterfeit production of products containing methanol [27]. In July, nine people were reported to have died in Kurichedu after drinking hand sanitiser for its alcohol content [28]. The death toll eventually rose to 15, with another 20 mildly symptomatic people identified through community surveys [29].<br><br>Cases of poisoning attributed to methanol-based hand sanitiser started emerging in USA in June 2020. In New Mexico, three people died, three were left in a serious condition and one person was blinded after ingesting methanol-based hand sanitiser [30]. The Arizona Poison and Drug Information Center also reported an increase in methanol poisonings across the state [31], with 37 methanol-based hand sanitiser exposures (including 5 fatalities) recorded between May 1 to October 14, 2020 [32]. Over the same reporting period in 2019, there were no methanol-based hand sanitiser exposures recorded by the Arizona Poison and Drug Information Center [32]. Research published by the US CDC concluded that between May 1 and June 30, there were a total of 15 serious methanol poisoning cases in Arizona and New Mexico, resulting in four fatalities and three cases of visual impairment [33]. Health officials from both New Mexico and Arizona suggested the methanol poisonings were likely associated with COVID-related hand sanitiser shortages, which had spurred new suppliers to manufacture hand sanitiser [31,34]. The US FDA urged consumers not to use a list of nine hand sanitisers manufactured by Eskbiochem SA de CV in Mexico because they were found to contain methanol. The US FDA also recalled batches of hand sanitiser produced by another two manufacturers, also made in Mexico, over concerns they

Consumer Products cont.	Hand Sanitiser containing methanol cont.	contained methanol [35]. From July 2020, the US FDA started releasing an up-to-date list of hand sanitisers that consumers should avoid after a number of adverse health events were recorded in adults and children [36]. The US FDA also reported that testing of hand sanitiser products produced in Mexico, labelled as ethanol-based sanitiser, revealed that a number actually contained methanol [36]. Another three hospitalisations and one fatality were reported in South Dakota in July [37]. By the end of July, the list of hand sanitiser brands to avoid that was published by the FDA had been expanded to over 100 products [37,38].<br><br>In October, the Republic of Ireland deregistered a particular brand of hand sanitiser for sale in the country and issued an alerts after testing showed it contained methanol [39]. In January 2021, the United Kingdom issued a similar alert for a different brand of hand sanitiser sold online, which was found to contain 37.4% methanol by weight [40]. In January 2021, the US FDA implemented a country-wide import alert on alcohol-based hand sanitisers imported from Mexico. The alert was put in place so that these products would be subject to increased FDA scrutiny after 84% of Mexican-origin products tested by the agency between April and December 2020 were found to be in breach of regulatory guidelines [41]. More than half of the products tested were also found to contain dangerous levels of toxic ingredients that were often not listed on the label, including methanol.<br><br>In November 2020 and March 2021, another two reports emerged of fatalities resulting from ingestion of methanol-based hand sanitiser in Russia and India. In November 2020, nine people from Sakha, Russia, were reported to have consumed methanol-based hand sanitiser at a party. Of the nine, seven were reported to have died and the other two were in a serious condition as a result of the exposure [42]. The five-litre tub of hand sanitiser had apparently been purchased form a local store, and authorities issued a recall notice to other pharmacies and stores in Sakha [42]. In March 2021, three brothers in the Indian city of Bhopal were reported to have died after ingesting methanol-based hand sanitiser. Although another national ban on alcohol does not appear to have been in place, the brothers were alleged to have consumed the sanitiser 'due to the non-availability of alcohol on March 21 due to Covid-19 restrictions' [32].
	Surge production of HS/ HS in drinks packaging	As high demand for hand sanitiser led to shortages in the early months of the pandemic, other businesses were urged to consider manufacturing hand sanitiser products if they had the capacity to do so. By April 2020, 1500 new manufacturers had registered to start producing hand sanitiser in the United States [43]. The US FDA released a statement thanking industry partners, but also urging them to follow FDA guidelines around denaturing alcohol even though this might incur them extra expense. The statement indicated that the FDA was made aware of an incident involving a 13-year-old who had consumed hand sanitiser packaged in a liquor bottle from a distiller which had not been denatured and therefore tasted like normal alcohol [43]. In August, the US FDA

	Surge production of HS/ HS in drinks packaging cont.	released a warning to consumers, urging them to be cautious of hand sanitiser in baby food and beer packaging [44].<br><br>In April 2020, the NSW Poisons Information Centre received nearly three times as many calls regarding hand sanitiser as it did in April 2019, attributed in part to the number of sanitisers being produced in the home and put in drinks packaging (often without childproof lids) [45]. In May, the British Colombia (B.C.) Poisons centre in Canada also reported increased hand sanitiser exposures in adults and children, also partly as a result of hand sanitiser being packaged in drinks bottles [46]. In June, Health Canada warned customers that many companies, fast-tracked to be able to produce hand sanitiser amid shortages, were having difficulty sourcing typical plastic packaging [47,48]. As a result, some companies were forced to use drinks packaging with similar labels to their regular products. Distillers in Canada used a variety of packaging usually reserved for liquor, including beer cans, wine bottles and liquor bottles [47,48]. Similar hand sanitiser supply issues were faced in Australia, with local companies also resulting to using packaging that is atypical for hand sanitisers. In June, one Australian distiller issued a recall notice after nine bottles of hand sanitiser were sold in gin bottles and labelled as gin [49]. In August, one woman from Manitoba, Canada, took to Facebook to warn other parents about hand sanitiser in atypical containers, after her mother accidently gave hand sanitiser to her 18-month-old thinking it was food [50]. The hand sanitiser had been packaged in a squeezable pouch (usually reserved for baby food or single-serve yoghurt) and had a children's cartoon character on the front. The toddler was hospitalised for observation as a precaution, but did not suffer any serious symptoms [50].
	Insect repellent (citriodiol)	In April 2020, the United Kingdom Ministry of Defence (MoD) announced that it would commence tests to see if citriodiol, the active ingredient in common insect repellent, could neutralise COVID-19 virus particles [51]. In May of that year, it was reported that the UK Secretary of State approved the use of citriodiol-based spray as a precautionary measure and the product has been distributed to at least ten Joint Military Commands [52]. Following the publication of some research by the MoD in August of 2020 (which found that Mosi-guard Natural Spray could inactivate some viable SARS-CoV-2 particles in liquid suspension, as well as following its application to latex 'synthetic skin' [53]) the manufacturer issued a warning to customers that their product should not be used to prevent COVID-19. The manufacturer, Pyramid Products, reported a 1,000% increase in online searches for their product in the 24 hours following the release of the MoD research [54].
Household Chemicals	Non-descript cleaning supplies	Media started reporting that poisons centres across the United States were observing increased cases of household toxic exposures and poisonings as a result of the COVID-19 pandemic in March and April of 2020 [11]. A report published by the American Association of Centres in May found that calls regarding disinfectant rose sharply in March and were approximately 120% higher in April 2020 than in April 2019 [55]. The report found

Household Chemicals cont.	Non-descript cleaning supplies cont.	that the highest incidence of toxic exposures was accidents affecting children under 5 years of age [55]. State poisons centres reported that the health risk attributed to household cleaning supplies affecting adults was multifaceted: people were at increased risk of exposure due to increased cleaning behaviours [1,9], were mixing different cleaning products together which produced toxic gas [9,11,56,57], some people were putting cleaning products on their skin or in baths to prevent COVID-19 infection [56,58], some people were cleaning food products with household cleaners [10,56], and others were ingesting household cleaners to prevent or treat COVID-19 [58,59]. Reports of increased calls and/or COVID-related exposures during this two-month period came from US states including Florida [59], Virginia [58], Illinois [56,60], New York City [61], Maryland [62], California [57], Arizona [1], Georgia [63], Kansas [63], Texas [9,64,65], Minnesota [66] and Michigan [10]. In April 2020, the British Columbia Poisons Control Centre also reported that they had witnessed a 60% increase in disinfectant exposure in people over 20 years of age [67]. In May, Health Canada reported that nationwide poison centre calls regarding hand sanitisers and household cleaning chemicals were 103% higher in March of 2020 than in March 2019 [13].<br><br>One factor that may have contributed to these figures was misinformation shared by high-profile figures. In April 2020, Cristina Cuomo, wife of CNN anchor Chris Cuomo and sister in-law to New York Governor Anthony Cuomo, wrote on her blog that she had treated her case of COVID-19 (in part) by adding half a cup of Clorox (household bleach) to her bathwater [68]. A press conference was also held by US President Trump on the 23rd of April 2020, where he made some comments about the ability of disinfectant to inactivate the virus. Trump turned to Dr Deborah Birx, the co-ordinator of the White House COVID-19 response, and seemed to suggest that it would be interesting if researchers investigated introducing disinfectant to the body, possibly by injection. The New York City Poisons Centre reported that the number of calls they received in the 18 hours following that press conference were double the number they fielded in the same reporting period for 2019 [61]. Maryland Poison Centres reportedly received hundreds of calls for advice on whether to ingest cleaning products [62]. An increase in calls following the press conference was also reported by Michigan Poison Centre [10]. Illinois Poisons centre reported increased calls in the 48 hours following the conference [61], including at least two men who exposed themselves to household disinfectants [60]. In Florida, between the 23rd and 27th of April 2020, the local poisons centre reported it had received 17 phone calls (as well as various emails and messages through social media) requesting advice on how to safely ingest cleaning products to prevent or treat COVID-19 [69]. One such request for information received by the Florida Poisons Centre came from a woman asking whether administering cleaning products to a child should be achieved through ingestion or injection [69]. Two men in Georgia and one

Household Chemicals cont.	Non-descript cleaning supplies cont.	man in Kansas also reportedly ingested liquid household cleaning products, supposedly influenced by the press conference [63].<br><br>A representative from the Belgian Poison Control Centre reported similar cases involving people being exposed to mixed cleaning products and putting household cleaning products on their skin or in their bath [70]. A representative from the Belgian Poison Control Centre, which recorded twelve times the number of bleach-related incidents in April 2020 as they had in April 2019, also reported a discernible spike in calls after President Trump's press conference [70].<br><br>In June 2020, research on toxic exposures resulting from efforts to prevent COVID-19 infections was published in the United States. A total of 502 adults were recruited and undertook a survey, which found 19% of participants had attempted to disinfect food items with household cleaners, 18% had used household cleaners directly on their skin, 10% had misted themselves with cleaning products, 6% had inhaled cleaning product fumes, and 4% had ingested or gargled household cleaners or soapy water [71]. Of those who had exposed themselves to household cleaning products, one quarter reported at least one adverse health outcome that they attributed to the exposure. Adverse health outcomes included irritation of the eyes, skin or throat, headaches, difficulty breathing and feelings of dizziness or nausea [71].<br><br>Between July 2020 and March 2021, other poisons centres in North America also issued or reiterated reports that call and poisoning data since the start of the pandemic was higher than in 2019. These poisons centres included the South Texas Poisons Center [22,72], North Texas Poisons Center [65], Upstate New York Poisons Center [23] and the Alberta Poison and Drug Information Service [25]. The Upstate New York Poisons Center noted that their highest call volume was in March 2020, when typically they would experience their highest volume of calls in summer months for the Northern Hemisphere [23]. Health Canada also issued new warnings not to mix household cleaning products in February 2021, cautioning that social media posts which claimed combining household cleaners made them more effective were misleading [73].
	Fish Tank Cleaner	In March 2020, it was reported that a couple in Arizona, USA, ingested a solution used to clean fish tanks because the active ingredient was chloroquine phosphate [74]. The man died as a result of the exposure and his wife was left in a critical condition. The incident occurred within five days of US President Donald Trump announcing at a press conference that an anti-malaria drug, (hydroxy)chloroquine, was a 'game changer' in combating COVID-19 [75].

Household Chemicals cont.	Pool/ hot tub chemicals	The IWK Regional Poison Centre in Canada reported that it had received significantly more calls regarding exposure to toxic pool chemicals in June 2020 compared to June 2019. A spokesperson from the poisons centre quoted in the media report suggested that the exposures may be a result of people spending more time at home during the COVID-19 pandemic, leading to increased purchases of pools and hot tubs [76].
	Kerosene/ PM2.5 by-products	Researchers conducting a study in Kenya published a news article in March 2021 indicating that COVID-related income loss and market changes may have pushed some consumers to revert to more polluting household energy sources. Results from household surveys suggested that access to cleaner forms of energy such as liquid petroleum gas or electricity had become more expensive, while price points for kerosene had fallen. While the results of the survey were quite nuanced and warrant further examination, the authors concluded that changes in market prices alongside more time spent at home under social distancing measures may drive increased exposure to hazardous levels of indoor air pollutants [77].
Industrial/ commercial chemicals	Disinfectant used in public spaces (often sodium hypochlorite, chlorine-based cleaners or bleach)	In March 2020, government authorities around the world started implementing mass sanitation measures in public places in order to prevent the spread of COVID-19. Public places including parks, mosques and markets in Tehran, Iran, were sprayed with disinfectant [78,79]. Officials in cities across China, South Korea, Iraq, Italy, Vietnam, Lebanon, Georgia, France, Spain, Russia and Brazil also organised mass sanitation efforts involving spraying disinfectant in public places [79-81]. In April and May of 2020, these efforts escalated, with efforts to disinfect public places by drone organised in Nigeria [82], Morocco [83] and India [84]. Supposed 'drone experts' in the UK also called for the government to amend regulations to allow for the same efforts to be undertaken [85]. An official in southern Spain was also forced to apologise after environmentalists and local community members expressed outrage that the local beach had been sprayed with bleach [86].<br><br>Efforts to sanitise public places resulted in some exposures and adverse reactions to cleaning products being reported in the media. In March 2020, India announced a national lockdown that resulted in a mass migration of daily wage earners out of cities to return to their rural homes. Video emerged of public workers, who had been sent to disinfect buses in the state of Uttar Pradesh, turning their hoses onto people and spraying down migrant workers [87]. In May 2020, three babies developed blisters, rashes and swelling after coming into contact with shopping carts from a Co-op store in Dauphin, Canada. The mothers of the infants, one of which experienced an oral reaction after kissing her baby's hand, suspected it was a result of chemicals used to wipe down the shopping trolleys the infants were placed in [88]. Then, in June, an investigation was launched into the death of a police doctor in the Philippines after a team conducting decontamination measures accidently sprayed him with concentrated sodium hypochlorite. Another two staff members on the medical team were also affected and were hospitalised [89].

	Disinfectant tunnels (often sodium hypochlorite)	In February 2020, China started deploying disinfection tunnels to prevent transmission of COVID-19. The tunnels allowed people to walk through aerosolised cleaners with the aim of inactivating any virus particles on the surface of their skin and clothes [90]. Between March and May 2020, disinfectant tunnels were also publicly trialled or installed in countries including Bosnia and Herzegovina [91], Sri Lanka [92], Pakistan [93], Chile [94], Mexico [95], Singapore [96] and Vietnam [97].
Industrial/ commercial chemicals cont.	Chlorine dioxide (MMS)	In manual media searches conducted in March 2020, reports from as early as January 2020 were found which suggested that online conspiracy movements such as QAnon were promoting chlorine dioxide to prevent or cure COVID-19 [98]. Also known as Miracle Mineral Solution (MMS), chlorine dioxide solutions had been promoted by a Florida-based church called Genesis II Church of Health and Healing and its founder, Jim Humble, for a number of years [99]. Chlorine dioxide is a highly toxic gas that is very soluble in water and primarily used for industrial water treatment in extremely low concentrations. The Genesis II Church advocates that customers mix chlorine dioxide solution in small amounts of water and ingest it for supposed health benefits. MMS first came to the attention of the US FDA in 2010 after it received reports of people having severe and life-threatening responses to ingesting the product [100]. MMS has historically been advertised as a treatment for diseases and disorders including but not limited to arthritis, urinary tract infections, high blood pressure, diabetes, depression, Parkinson's disease, autism, cancer, hepatitis, Lyme disease and HIV/AIDS [99,100].<br><br>Reports suggested MMS was being promoted online as a cure for COVID-19 in France as early as March 2020 [101]. In April 2020, the US FDA and Federal Trade Commission issued a joint warning to the Genesis II Church for advertising MMS as a means to prevent COVID-19 infections in both adults and children [102]. A federal court then issued a temporary injunction to prevent any further sales [102]. In May, a complaint against the church was also lodged to the Australian Therapeutic Goods Association (TGA), who issued an AU$151 200 fine for marketing of a fraudulent COVID-19 treatment [103,104]. A report from May 2020 also demonstrated that MMS was being marketed as a cure for COVID-19 in New Zealand [105]. The man reported as responsible for marketing MMS in New Zealand was also advocating breathing in chlorine dioxide gas produced by combining sodium chlorite with hydrochloric acid [106].<br><br>In July 2020, it was reported that a leader of the church and associate of its founder, named Mark Grenon, had ignored the US court order to cease operations. Documents obtained by the US FDA Office of Criminal Investigations demonstrated that Grenon and his sons, who were also involved in the business, had quadrupled their revenue in March 2020 when they started advertising MMS as a cure for COVID-19 [107]. The family had allegedly

Industrial/ commercial chemicals cont.	Chlorine dioxide (MMS) cont.	sold tens of thousands of bottles of MMS across the United States and were expected to receive criminal charges [108].<br><br>From July, evidence started emerging that misinformation around MMS was starting to disseminate in Central and South America. One report from Mexico indicates that advertisement of MMS was becoming prominent on social media [109]. The Director of Costa Rica's National Poison Control Center told media sources the poison centre was receiving 10 calls a day regarding MMS, prompting the Costa Rican Health Ministry to issue a warning [110]. Local reports suggested that at least thirteen people around Bolivia were poisoned after exposure to MMS [111,112]. A number of advocates for alternative medicine who were based in South America and had a history of promoting chlorine dioxide treatments, including a German national called Andreas Kalcker, also consulted the Bolivian opposition [111]. The Bolivian opposition went on to pass a bill in its Senate intended to guarantee the supply and use of chlorine dioxide in the health care system for the prevention and treatment of COVID-19 [112,113]. The bill passed in the opposition-controlled Chamber of Deputies in August, but was blocked from coming into law by the interim President, Jeanine Áñez [111,114]. On August 12th, Colombian authorities arrested Mark Grenon and one of his sons in Santa Marta, who were to be extradited to the United States [115,116].<br><br>The Pan American Health Organization (PAHO) issued a statement in August warning people to avoid chlorine dioxide products purported to prevent and treat COVID infections [117]. The Georgia State Department of Public Health also issued an alert after it received reports that people were using MMS to treat COVID-19 [118]. In September, an article from the United States suggested that chlorine dioxide-based water treatment kits being sold on Amazon were likely being ingested by customers. Despite these water treatment kits being marketed as unsuitable for human consumption, customers were leaving comments under products to share information about how they used the kits to prepare MMS treatments [119].<br><br>In January 2021, the stepson of a 92-year-old Argentinian man hospitalised with COVID-19 won a court battle against his doctors, with the judge ordering the doctors to administer MMS to the man in line with the stepson's wishes. The case drew widespread criticism from the medical community and MMS was administered to Oscar Jorge García Rúa after the clinic lost their bid to appeal the verdict. He died a few days later, on the 11th of January 2021 [120]. In March, Argentinian prosecutors raided laboratories suspected of producing MMS after a five-year-old boy and 50-year-old man died following ingestion of the product, which was being promoted on

Industrial/ commercial chemicals cont.		Facebook [121]. The raids were reportedly linked to an investigation into Andreas Kalcker, who had also helped popularise MMS use in Bolivia [121].
	Sodium chlorite (often sold alongside hydrochloric acid to produce chlorine dioxide/ MMS)<br><br>Sodium chlorite (often sold alongside hydrochloric acid to produce chlorine dioxide/ MMS) cont.	In June 2020, a report from New Zealand suggested the man reported as responsible for marketing MMS in the country was also advocating breathing in chlorine dioxide gas produced by combining sodium chlorite with hydrochloric acid [106]. The warnings put out by PAHO and the Costa Rican Health Ministry suggest this method of exposure to chlorine dioxide was also a cause for concern in the Americas and Caribbean [110,117].<br><br>The Spanish Civil Guard arrested a woman from Piedratajada in June after she allegedly sold her neighbour a sodium chlorite solution as a cure for COVID-19. The solution left the 67-year-old woman with sever poisoning symptoms. When the Spanish Civil Guard searched the home of the woman arrested they found more chemical products and €32,000 in cash [122].<br><br>In September 2020, an investigative piece published by the BBC identified two people in United Kingdom who sold undercover reporters sodium chlorite and hydrochloric acid in order to make MMS [123]. One of the people identified, Ann Gylman, suggested that her sales of the products had increased after Trump's press conference on the 23rd of April, where he proposed researching bleach and introducing it to the body to prevent COVID-19 [123].
	Styrene gas	In May 2020, a styrene gas leak occurred at a LG Polymers facility in Andhra Pradesh, India. Thirteen people died as a result of the chemical incident and another 1000 people were hospitalised. The leak occurred after two tanks storing the gas were left unattended for over 40 days under COVID-19 lockdown measures [124].
	HDQ Neutral (ammonia-based disinfectant)	In August 2020, it was reported that immigrants held in US Immigration and Customs Enforcement (ICE) detention facilities in the United States were being exposed to HDQ Neutral, which was being utilised to prevent the spread of COVID-19. HDQ Neutral is an industrial, corrosive disinfectant that should not come into contact with the eyes or skin and is harmful if inhaled. It was reported that guards were spraying the disinfectant indoors every 15 to 30 minutes, sometimes in the vicinity or in the direction of inmates. An advocate for the inmates indicated that some had shown symptoms as a result of exposures, including haemoptysis, nausea, migraines, and irritation of the eyes and skin [125].

Industrial/ commercial chemicals cont.	Mixture of nitric oxide[1], sodium nitrate & sodium hydrate	In May 2020, a pharmacist from India died and his boss was hospitalised after they ingested a chemical solution the pharmacist had developed to prevent or treat COVID-19. Media reports said the two men worked for an herbal medicine firm and that the remedy was a mixture of nitric oxide and sodium nitrate obtained from local markets. The pair also reportedly ingested sodium hydrate during experiment [126].
	Gasoline	In July 2020 the President of the Philippines, Rodrigo Duterte, suggested that people should use gasoline to sterilise or disinfect face masks. After a spokesperson for the President indicated that the suggestion was a joke, Duterte appeared on television and repeated the suggestion, stressing that he was not joking [127].
	Ethylene Oxide	In June 2020, reports emerged that ethylene oxide-emitting industrial facilities in the US State of Georgia, which had either closed or were under pressure to close due to associated long-term health risks, had reopened or increased their production in order to meet increased demand for sterilised personal protective equipment [128].
	Graphene	In March 2021, the provincial government of Quebec halted the distribution of face masks that contained graphene to schools over concerns they may pose a health risk [129].
	Silver and titanium oxide nanoparticles	In February 2021, the Belgian government halted the distribution of facemasks found to contain nanoparticles of silver and titanium oxide. Over 5 million of the masks had been distributed for free through pharmacies in Belgium since June 2020 [130].
	Unspecified toxic gas	In May 2020, seven workers fell ill at an Indian paper mill in the village of Tetla while cleaning a recycling chamber in preparation for the mill to reopen following COVID-19 lockdown measures. Three of the seven workers affected experienced serious symptoms as a result of exposure to the unspecified toxic gas [131].
	General	The Belgium Poisons Control Centre reported experiencing a spike in drug poisoning cases in April of 2020 compared to April 2019 [70].
	Cocaine	In March of 2020, the French Ministry of Social Affairs and Health released a tweet warning people that online rumours suggesting cocaine use prevented COVID-19 infections were false [101].
	Opioids	In April 2020, reports started emerging that COVID-19 was compounding opioid-related public health crises in North America. Public health officials and police in Toronto reported a spike in overdose deaths, including two where naloxone had been administered [132]. One official suggested the risk of overdose had increased, in part, because of social distancing measures and the increased risk of doing opioids in isolation [132]. However, Ontario's Chief Medical Officer also indicated that the composition of illicit drugs in Canada was also in a state of flux, with the levels of fentanyl present in drugs constantly changing [133]. The presence of increasingly toxic

[1] All media sources on this case report the gentlemen drank the solution and most report that the chemicals were brought at a local market. As nitric oxide is a gas at room temperature, it may be that the chemical has been misidentified or misreported.

Recreational drugs/Drugs of Misuse		preparations in Canada was thought to be connected to COVID-related border closures, as lower levels of supply may be driving people to cut available drugs with other substances [134]. Overdose deaths in Vancouver and Peterborough, Canada, were also reported to have spiked following the onset of the COVID-19 pandemic and social distancing measures [133,135,136]. In Peterborough, officials warned that any drug could be unusually toxic and pose an overdose risk after crystal methamphetamine and cocaine were suspected to have been involved in a fentanyl-related death [136]. In July, officials in Cook County, Chicago, also reported a spike in opioid-related deaths predominantly affecting ethnic minority communities. However, Cook County hospitals had not recorded an associated increase in opioid-related visits, prompting concerns that COVID-19 and lockdown measures may have been discouraging people from seeking medical attention [137]. In July, a 14-year-old Indigenous girl also died of an overdose on Vancouver Island [138].<br><br>Official figures and analysis into the frequency and distribution of deaths in Canada started emerging in the latter half of 2020. The Chief Coroner of Ontario indicated that between March and May of 2020 drug-related deaths were 25% higher than the same period in 2019 [139]. An Ontario Drug and Research Policy network report also found that the age distribution of opioid-related deaths had not changed, but that the proportion of opioid-related deaths in men, low socioeconomic areas and communities with high ethno-cultural diversity had increased [139]. The province of Alberta released official figures that showed record numbers of opioid-related deaths in 2020, with concerns that Indigenous populations and men were disproportionately affected [140]. In December 2020, Toronto Paramedic Service recorded the highest number of opioid-related deaths in a single month (34) since Toronto Public Health started monitoring that outcome in 2017 [141]. Suspected opioid overdoses in Toronto were also 90% higher in 2020 than in 2019, with testing revealing that drug supplies contained highly toxic and atypical ingredients (some of which would not respond to naloxone treatment) [141]. In January 2021, Toronto Paramedic Service also received a record number of calls for suspected opioid overdoses in a 24-hour period (40). Between the 1st of January and the 26th of January 26, 2021, Toronto Paramedic Service attended 30 opioid overdoses that resulted in fatality [142].
Recreational drugs/Drugs of Misuse cont.		In July 2020, a report from Afghanistan suggested that COVID-19 impacts were compounding opioid use and addiction. The article indicated approximately six million Afghans had lost their job as a result of the pandemic, with mounting financial pressures driving opioid use. The article also quoted health workers who suspected that people were avoiding hospitals and drug treatment services (which had been increased during the pandemic) for fear of contracting COVID-19 [143].

	Cannabis	The Hospital for Sick Children, Toronto, reported an overall decrease in emergency admissions in 2020, but a 17% increase in accidental ingestions and poisonings. An increase in cannabis edible ingestions underscored part of this overall increase. The author of a media article describing these trends (a physician at the hospital) attributed the increase in poisoning accidents in children, in part, to pressures faced by parents to supervise their children under lockdown measures [144].
Dietary Supplements	Colloidal Silver	In March 2020, an American Evangelist started promoting a nano-silver solution as a supposed cure of a COVID-19 infection on a television segment [145]. In April, the US FDA announced that they had written to several companies, warning them to cease advertisement and sale of colloidal silver as a means to prevent or treat COVID-19 infections [146,147]. In April and May, the US Justice Department and Federal judges also issued some court orders prohibiting specific individuals and companies in Utah and Oklahoma from selling colloidal silver products for COVID-19 infections [148,149].
	Vitamins	In April 2020, there was an indication that consumers in the United States were increasing their purchase of vitamins, supplements and cold and flu remedies in an effort to prevent COVID-19 infections [147]. Cristina Cuomo, wife of CNN anchor Chris Cuomo and sister in-law to New York Governor Anthony Cuomo, wrote on her blog that she overcame her case of COVID-19, in part, with the use of vitamin drips [68]. In February 2021, the Upstate New York Poison Center reported that it saw an increase in a variety of poisonings in 2020 that it attributed to people trying to prevent and treat COVID-19, including an increase in calls regarding vitamins [23].
	Vitamins cont.	In September 2020, a consulting endocrinologist in Mumbai reported that one of her patients was suffering asymptomatic serum vitamin D toxicity (348 ng/ml) after exceeding recommended doses of vitamin D supplements. Dr Tejal Lathia said the patient indicated they had seen messages on social media about how vitamin D could boost immunity and prevent COVID-19 infection, and that many of her patients were trying to boost their immunity using supplements and homeopathic remedies [150].
Dietary Supplements cont.	General	In July 2020, the South Texas Poisons Center reported that accidental ingestion of over-the-counter supplements had increased in 2020 due to more time spent in (sometimes multigenerational) homes. The Center reported a 47% increase in questions about melatonin specifically, and attributed some accidental ingestions to gummy supplements that appeal to children [151].
	Traditional Chinese medicine	In April 2020, the Chinese government advised doctors to combine Western pharmaceuticals with traditional Chinese medicine when treating COVID-19 patients [152]. Government promotion of traditional Chinese medicine was also directed overseas, with reports suggesting the Chinese government shipped traditional medicines as aid to countries that were severely affected by COVID-19 outbreaks, including Italy and Iran [153].

Herbal, Homeopathic or Traditional medicine	Madagascar remedy (artemisia)	In April 2020, the President of Madagascar, Andry Rajoelina, announced that the country would commence tests to analyse whether a local plant-based remedy would be effective in combating COVID-19 infections [154]. By May, President Rajoelina announced that the remedy, made of artemisia and other native herbs, had already successfully treated two people [155]. Shipments of the remedy (called Covid-Organics) had also begun arriving in countries across Africa, including Equatorial Guinea, Guinea-Bissau, Tanzania, Central African Republic, the Democratic Republic of Congo and Liberia [156,157]. President Rajoelina suggested that researchers were looking into a potential injectable preparation of the remedy, though this was later dismissed by another government official [158]. In May 2020, the New Zealand Medicines and Medical Devices Safety Authority announced that it had blocked the importation and sale of the remedy in New Zealand [159]. In March 2021, President Rajoelina made televised comments that he was not personally vaccinated and was in no rush to vaccinate the people of Madagascar, as he preferred the use of the herbal remedy over vaccinations [160].
	Other herbal remedies	In March 2020, a photo of a prescription for an Ayurvedic remedy was shared thousands of times on social media in Sri Lanka alongside claims that it was effective against COVID-19 [161]. In April, the Prime Minister of India, Narendra Modi, made comments suggesting boosting immunity to combat COVID-19 was important. Prime Minister Modi suggested the Ministry of Ayurveda, Yoga, Naturopathy, Unani, Siddha, Sowa-Rigpa and Homoeopathy (AYUSH) was looking to promote a ready-made preparation [162]. The Archbishop of the Douala, Cameroon, and a Catholic priest in Nigeria also suggested they had developed herbal remedies that could treat and cure COVID-19 [163,164]. Cameroonians subsequently went to purchase the remedy promoted by the Archbishop, who went on to claim he had treated dozens of people including health workers [165]. The President of Cameroon, Dion Ngute, held a meeting with Archbishop Samuel Kleda in May, who went on to claim that over 3000 people had been successfully treated with his remedy by June [166]. In May, health officials in Afghanistan issued public warnings not to take unproven remedies after video clips started being shared on social media regarding a supposed remedy from a healer in Kabul [167]. Hundreds queued outside the home of the healer in order to purchase the remedy, which the Afghan Ministry of Public Health found to contain opioids including morphine and codeine [168].
	Other herbal remedies cont.	In June, a cardiologist from Kerala, India, wrote on Twitter that she had attended a patient who had developed severe liver disease after ingesting a herbal remedy supposed to prevent COVID-19 [169]. In July, a media report suggested that many Zimbabweans, who had been priced out of the biomedical healthcare sector, were turning to traditional medicine to treat COVID-19 [170]. In September, a Nigerian biotechnologist had also started claiming that he had made a herbal remedy for COVID-19, suggesting that it was more potent that the Madagascan remedy [171]. In October, Venezuelan President, Nicolas Maduro, notified PAHO that a molecule which

Herbal, Homeopathic or Traditional medicine cont.		interfered with the replication of the novel coronavirus had been identified by Venezuelan scientists. Following the notification to PAHO, he didn't appear to promote this molecule again, but in January 2021 promoted a liquid remedy called carvativir which was administered under the tongue [172].<br><br>In December 2020, thousands of people queued outside the home of a healer in Sri Lanka who had claimed to have a remedy that gave consumers lifelong immunity against COVID-19. Several high-ranking politicians including the Minister of Health and the Minister for the Development of Women and Children ingested the remedy though it was not formally endorsed by the government [173,174]. In February 2021, Tanzania's Health Ministry encouraged citizens to use local traditional medicine to treat COVID-19 amongst other infectious diseases and respiratory conditions such as asthma [175].
	Oleander	In August of 2020, it was reported that the CEO of a company called MyPillow had met with US President Donald Trump and Housing and Urban Development Secretary, Ben Carson, in July to discuss an oleander extract produced by the company. In the weeks following, Donald Trump had had reportedly urged the US FDA to approve the drug for use alongside other COVID-19 treatments [176]. The American College of Medical Toxicology (ACMT), the American Academy of Clinical Toxicology (AACT), and the American Association of Poison Control Centers (AAPCC) subsequently released a joint statement to express concerns about the proposed use of oleander on COVID-19 patients, particularly in light of the fact that it could be toxic to humans [177]. In November, Ben Carson tested positive for COVID-19 and made public comments that he had taken oleander extract to treat his infection on the advice of MyPillow CEO, Mike Lindell [178]. A local media outlet in Florida reported that sales of Olendar extract had surged following Ben Carson's comments [179].
Herbal, Homeopathic or Traditional medicine cont.	Essential Oils	In March 2020, there was some evidence that people in the United States were buying and using essential oils as a means to prevent COVID-19 infections [145]. By April, the US FDA had already sent warnings to several companies about marketing essential oils as a preventative measure or treatment for coronavirus [147]. An Australian Celebrity chef, Pete Evans, also started advertising essential oils as a way to combat COVID-19 infections. In a separate Instagram post, he had promoted an upcoming collaboration with essential oils company, doTerra, and shared a picture of a fish dish he indicated contained essential oils [180]. In May, a representative from the Belgian Poisons Control Centre indicated that there had been recent cases of people applying essential oils directly to their skin in an attempt to wash themselves and prevent COVID-19 infections [70].
	Ephedra	In August 2020, Australian authorities issued a warning urging Australians not to import medicines rumoured to prevent or treat COVID-19. The Australian Border Force had intercepted an increasing amount of an herbal medicine called ephedra. Officials reported that between January and March 2020 they had detected two kilograms of

		the substance, but between April and May they had detected 66 kilograms, which led them to believe it was associated with the COVID-19 pandemic [181].
Animal, plants and fungi	Snakebite	Between June and August 2020, poisons centres and hospitals in US states including Arizona, California, Nevada, Texas, North Carolina, South Carolina and Mississippi reported increased rates of snakebite cases compared to 2019 [182-185]. Physicians and Poisons Centre specialists quoted in reports indicated that COVID-19 lockdown measures were likely a contributing factor, as people were spending more time outdoors and in home gardens [182,183,185].
	Mushroom foraging	In May 2020, reports emerged that the number of calls regarding potentially toxic fungi exposures received by poisons centres in the Australian state of Victoria [186] and the US state of Florida had increased [187]. The report from Australia, and another report published in September from Ontario, Canada, indicated that interest in mushroom foraging had been growing amongst local communities in recent years [186,188]. However, in Australia, coronavirus restrictions and excellent growing conditions were thought to contribute to some of the interest in mushroom foraging in 2020 [186]. An expert from the University of Florida also attributed increased fungi exposure calls to people spending more time at home due to lockdown measures, indicating that the increase in calls had occurred in Spring, when calls for information on fungi were typically less frequent [187]. In Ontario, walking tours and workshops held to help educate people on foraging safety had been cancelled due to COVID-19 restrictions, though poisoning calls were still comparable to previous years [188].<br><br>In November, the start of the mushroom picking season in France also resulted in a spike in calls to poisons centres. Between the 1st of July and the 23rd of October, there were reportedly 732 cases of mushroom poisoning including five that were severe. The French Agency for Food, Environmental and Occupational Health & Safety (ANSES) urged people to take extra care, as the health system was already under strain from the ongoing COVID-19 pandemic [189]. In March 2021, warnings about mushroom toxicity in the Australian state of Victoria were renewed, with Victorian Department of Health data indicating that the number of calls regarding potential mushroom poisoning in 2020 (426) were double the number of calls in the prior two years. The record number of calls was again attributed to more time being spent outdoors due to COVID-19 restrictions and an optimal growing season [190].
	Giant Hogweed	In May 2020, local organisations in Scotland warned that normal activities to clear giant hogweed, a plant that can cause serious burns and blindness, had been hampered by COVID-19 restrictions [191]. By June, reports indicated that hogweed was growing and proliferating in areas that had previously not been affected by the plant [192]. In August, signs were placed in areas of South Glasgow warning that hogweed was extremely toxic and normal efforts to control it had been 'impossible in 2020 due to COVID-19' [193].

Animal, plants and fungi cont.	*Datura stramonium*	In April 2020, ten people were admitted to hospital in India after ingesting juice from *Datura stramonium* seeds. Reports suggest that the remedy had been promoted on Tik Tok as a means to prevent or cure COVID-19 [194].
	Quina plants	In June 2020, a tea made from Quina plants was being promoted as being able to prevent or treat COVID-19 on social media in Brazil. Plants of the same genus from Peru were sources of quinine, which inspired the drugs chloroquine and hydroxychloroquine. However, Quina trees native to Brazil do not contain quinine and some are known to be toxic to humans [195].
	Algal blooms	In July 2020, the Utah Department of Environmental Quality indicated that COVID-related budget cuts meant it did not have the capacity to undertake usual efforts to warn people about algal blooms in public water bodies. In 2019, 38 algal blooms across the state had resulted in public health warnings and there were 40 calls to poisons centres associated with the algal blooms [196]. Projects to warn citizens, monitor and research algal blooms in Florida were also adversely affected by COVID-19 [197].
	Liquorice root	In December 2020, Turkmenistan's President, Gurbanguly Berdymukhamedov, suggested that liquorice root was an effective cure for COVID-19, resulting in an increase in the market price [198].
	Plants General	In February 2021, the Upstate New York Poisons Center reported that in 2020 there was an increase in some forms of poisoning as people tried to combat COVID-19, spent more time outdoors and more time at home under lockdown measures. The Poisons Center reported that calls about exposures to plants was one such area in which calls for 2020 had increased on 2019 numbers [23].
Heavy Metals	Lead	In July 2020, health authorities in Newhaven, Connecticut, started monitoring 27 children for elevated lead levels. Twenty of the cases had been identified in a five-week period as normal healthcare functions resumed, following COVID-19 restrictions that had hindered routine services and delayed the delivery of care [199].
Human pharmaceuticals	(Hydroxy)chloroquine	On the 19th of March 2020, Trump announced at a press conference that the US FDA were looking at the therapeutic effect of (hydroxy)chloroquine on COVID-19, suggesting 'it could be a game changer' [75]. Trump highlighted that a benefit of using hydroxychloroquine in this context was that it was an existing drug that had been used for some time, therefore its use was considered safe and there were unlikely to be any serious adverse reactions or associated deaths. By the 23rd of March, Nigerian Health Authorities had issued warnings after the price of hydroxychloroquine spiked and three people who had overdosed with the drug were hospitalised [200]. By the 31st of March, Brazilian President Jair Bolsonaro had posted a video to Facebook suggesting that hydroxychloroquine was a totally effective treatment against COVID-19, which was deleted by the social media service [201]. Australian pharmacies reported that there had been a rush on pharmacies to secure the drug following Trump's press conference [202]. In Assam, India, colleagues of a doctor who had self-medicated with the drug said he had subsequently died of a cardiac arrest [203].

Human pharmaceuticals	(Hydroxy)chloroquine cont.	In April 2020, the raw products used to make hydroxychloroquine were reported to be ten times their usual price [204]. In the Democratic Republic of Congo, hydroxychloroquine was six times its usual price and consumers in Zambia were stockpiling the antimalarial (as well as other pharmaceuticals including antibiotics) [204]. In the USA, self-medication with hydroxychloroquine led to shortages that affected people who were prescribed it to treat autoimmune disorders [205]. In India, the government banned exports of hydroxychloroquine while some doctors and contacts of lab-confirmed cases were given it experimentally [206].<br><br>In April 2020, a trial of hydroxychloroquine amongst COVID-19 patients in Sweden was halted after some participants presented with side effects including migraines, muscle cramps and peripheral vision loss [207,208]. Experimental administration of the drug at a hospital in France was also ceased after it was assessed to be a 'major risk' to a patient's cardiac health [209]. A Brazilian medical trial also ended after just 13 days when the number of deaths recorded amongst the high-dose group (16/41 patients) were significantly higher than deaths amongst the low-dose group (5/40 patients) [210,211]. The American Association of Poison Centers reported that calls regarding hydroxychloroquine over March and April were double that of previous years [212]. A COVID-positive, 65-year-old woman from New York who was prescribed hydroxychloroquine and azithromycin by her doctor also died of cardiac arrest after taking three doses of the medication [213].<br><br>In May, the US Justice Department started investigating a doctor who was suspected to be promoting the drug [214]. The Australian Border Force also issued warnings about illegally importing and self-medicating with the drug after customs intercepted dozens of shipments containing over 6000 tablets in total [215]. A report from the United States suggested that nursing homes in the state of Pennsylvania had started administering hydroxychloroquine to residents, sometimes without the knowledge or consent of their next of kin [216]. The US Department of Veterans Affairs also faced criticism for the use of hydroxychloroquine in its government-run hospitals, particularly after researchers found that COVID-positive veterans who were given hydroxychloroquine were dying at higher rates (28%) than COVID-positive veterans who were just given routine care (11%) [217]. On the 19th of May, President Trump told reporters that he had been taking hydroxychloroquine for over a week and that some frontline health staff were also on courses of the drug [218,219]. The Brazilian Health Ministry incorporated hydroxychloroquine into national treatment guidelines, with President Bolsonaro suggesting that he kept a box of the drug aside for his elderly mother as a precaution [220].<br><br>As the WHO acknowledged that hydroxychloroquine had prompted some safety concerns amongst researchers, the French government indicated clinicians should not prescribe hydroxychloroquine for the purposes of treating

Human pharmaceuticals cont.	(Hydroxy)chloroquine cont.	COVID-19 [221]. Despite these safety concerns, the Indonesian government was reportedly planning to administer hydroxychloroquine to COVID-19 patients [222]. Meanwhile, the US Veterans association provided data to Congress which showed that weekly prescriptions of hydroxychloroquine rose from 2 per week to a peak of 404 after Trump promoted the drug, with 1,370 veterans prescribed it in total before prescriptions subdued in late April [223].<br><br>In June 2020, the WHO announced it would resume clinical trials involving hydroxychloroquine, after they were briefly paused for safety concerns [224]. However, in July, the Solidarity Trial's International Steering Committee recommendation to discontinue trials involving hydroxychloroquine were accepted by the WHO. Interim results published in October 2020 showed that none of the four treatments tested (remdesivir, hydroxychloroquine, lopinavir/ritonavir and interferon) significantly improved outcomes including mortality or length of hospital stay in COVID-19 patients [225].<br><br>In June 2020, emergency authorisation for use of hydroxychloroquine on COVID-19 patients was revoked in the United States. The US FDA suggested that the risks of administrating the drug were too high given its efficacy for the treatment of COVID-19 had still not been established, a decision that Trump criticised [226]. In Brazil, however, clinical guidelines for administration of hydroxychloroquine were expanded to cover pregnant women and children [227]. In July, when President Bolsonaro tested COVID-positive, he indicated that he was personally taking hydroxychloroquine as a treatment [228]. Back in the United States, a 17-year-old, COVID-positive girl with pre-existing health issues died after being administered hydroxychloroquine and treated at home by her parents for six days before seeking formal medical attention [229].<br><br>In August, there were concerns as an experimental drug treatment that contained hydroxychloroquine was being prepared to be rolled out across Indonesia without full clinical trials [230]. Warnings around importing the drug were also reissued in Australia after 16,000 antimalarial medications including hydroxychloroquine (which was only detected in minimal amounts pre-pandemic) were intercepted between May 8 and June 21, 2020 [181]. An Australian government minister, Craig Kelly, made public comments questioning whether the Premier for the state of Victoria, a member of the opposition party attempting to contain one of the largest outbreaks in Australia, was criminally liable for blocking the use of hydroxychloroquine [231]. Minister Craig Kelly had been sharing misinformation about COVID-19 measures and hydroxychloroquine on Facebook throughout the pandemic and by February 2021 the Shadow Health minister in Australia indicated that his posts were getting four times the engagement of the Department of Health website [232].

Human pharmaceuticals cont.	(Hydroxy)chloroquine cont.	In December 2020, a doctor from San Diego, USA, faced charged after he allegedly tried to sell fraudulent COVID-19 treatment kits containing illegally-acquired hydroxychloroquine at inflated costs [233]. However in Brazil, a cocktail of drugs became so widely adopted as an early treatment for COVID-19 (ivermectin, azithromycin, and hydroxychloroquine) that Brazilians started referring to it colloquially as a 'COVID kit' and officials in the city of Barra do Garças distributed packages of the drugs for free [234]. In March 2021, the Estadao, a Sao Paulo newspaper, reported that 'COVID kits' were linked to at least three deaths and five liver transplants [235].
	Ivermectin	In April 2020, an Australian study that showed high doses of ivermectin could inhibit COVID-19 from replicating in cells *in vitro* was reported in the media [236]. Australian health authorities immediately warned people against self-medicating with the drug [236]. These warnings against self-medication were echoed by the New Zealand Ministry of Health [237]. However, soon thereafter, gastroenterologist Professor Thomas Borody appeared on an Australian media network (which is also broadcast in New Zealand) promoting misinformation around ivermectin and a rush on Australian pharmacies for the drug was reported [238,239].<br><br>In June, it was reported that *in vitro* studies on ivermectin and a pre-print from the US company Surgisphere Corporation (which was subsequently withdrawn over methodological concerns) saw ivermectin become a prominent treatment for COVID-19 in Latin America [240-242]. In May, Northern Bolivian health workers had handed out 350,000 free doses of the drug [241]. Ivermectin soon became incorporated into clinical guidelines and endorsed by governments in Peru, Bolivia and Brazil [240]. As people began requesting the drug and self-medicating, supplies intended for human use ran low and people started turning to veterinary preparations of the drug (see 'ivermectin' under the 'veterinary pharmaceuticals' section) [240,241]. Use of ivermectin continued to surge across Latin America and researchers in Peru soon warned that they were having difficulty recruiting study participants who were not already taking the drug [241].<br><br>By January 2021, Facebook posts endorsing ivermectin as a treatment for COVID-19 had been observed in Brazil, France, South Africa and South Korea [242]. Leading experts in South Africa warned that social media hype around the drug was irresponsible given that there was currently no evidence to suggest it effectively treated COVID-19 and as it had associated safety concerns [243]. At the time, ivermectin was not approved for any human use in South Africa and regulatory authorities soon reported a surge in illegal importations of the drug [244]. With no human preparations available, South Africans also started taking veterinary preparations of the drug (see 'ivermectin' under the 'veterinary pharmaceuticals' section).

Human pharmaceuticals cont.	Ivermectin cont.	In February 2021, Australia's Shadow Health Minister wrote to the Managing Director of Facebook in Australia after a Government Minister, Craig Kelly, was able to promote misinformation about hydroxychloroquine and ivermectin on its platform throughout the pandemic [232]. The President of the American Association of Poison Control Centers also indicated that there had been a recent surge in calls regarding ivermectin, including one case where someone had taken a preparation of the drug made for horses [245]. Ivermectin was also considered part of 'COVID kits' in Brazil, a cocktail of drugs promoted and widely used as an early treatment [234]. These 'COVID kits' (which also included azithromycin and hydroxychloroquine) were linked to at least three deaths and five liver transplants, as reported by a Sao Paulo newspaper, the Estadao [235].<br><br>When the research period for this study concluded in March 2021, there was some evidence that misinformation around ivermectin was spreading further around Europe. Misinformation around ivermectin was reportedly spreading on Facebook in Ireland, where at least one video promoted it as an alternative to COVID-19 vaccines [246]. The European Medicines Agency also indicated that online rumours around ivermectin were misleading, that ivermectin was not effective in treating COVID-19 and that it was not authorised for such use in the European Union [247]. As black market sales and prices of ivermectin continued to climb in South Africa, authorities approved the drug for human use only on compassionate grounds that were subject to regulatory oversight [248].
	Prescription/ Medical grade Opioids	In March 2021, the Washington Poison Center reported a 3.6% rise in prescription opioid exposures. The increase in exposures was considered to be, in part, driven by the effects of the COVID-19 pandemic on the mental health of teens and young adults, as well as on increased accidental exposures amongst children in the home [249].
	Avigan/favipiravir	In May 2020, Japanese Prime Minister, Shinzo Abe, advocated for use of the drug Avigan for COVID-19 treatment and allocated approximately $130 million to increase existing stockpiles. There was no evidence that Avigan improved outcomes in COVID-19 patients and it had previously been linked to birth deformities in children [250].
	Counterfeit pharma	In April 2020, people started stockpiling hydroxychloroquine internationally, leading to a surge in counterfeit medicines. The BBC found fake hydroxychloroquine was being sold in the Democratic Republic of Congo, Niger and Cameroon [204].
	Famotidine/antacid	In April 2020, rumours that famotidine was being tested on COVID-19 patients in New York City circulated online in the Unites States [251]. Consumers soon started stockpiling famotidine-based heartburn medication, leading to shortages [252].
		In April 2020, an Australian study showed high doses of ivermectin could inhibit COVID-19 from replicating in cells *in vitro* was publicised in the Australian media [236]. The US FDA subsequently published a letter to

Veterinary pharmaceuticals	Ivermectin	stakeholders, as the Center for Veterinary Medicine noted the increased public profile of the drug and became concerned some people may self-medicate with veterinary preparations [253].<br><br>From May 2020, as interest in ivermectin in Latin America started to surge and the drug was incorporated into clinical guidelines, lower supply levels saw some people turn to veterinary preparations [240,241]. One physician quoted in the media said he had seen patients who had developed dermal blisters where a veterinary form of the drug had been injected, alongside other side effects including gastrointestinal upset, tremors and panic attacks [240].<br><br>In December 2020, the US FDA issued public guidance on ivermectin, indicating that human and veterinary preparations had not been approved for, nor were suitable for, treatment of COVID-19 infections [254].<br><br>In January 2021, South Africans seeking to use ivermectin as a COVID-19 treatment were believed to have turned to oral, injectable and pour-on veterinary preparations. A leading toxicologist from the Griffon Poison Information Centre, Dr Gerhard Verdoorn, was quoted in the media over concerns that people may have been exceeding recommended doses used for treating parasites in livestock [255]. It was reported that in two weeks of January 2021 that veterinary ivermectin manufacturers sold four months' worth of stock [256].<br><br>In February 2021, a former Senator from Australia, David Leyonhjelm, was threatened with an AU$1.11 million fine for sharing and initially refusing to delete misinformation around ivermectin that contravened laws around the advertisement of medical products. Mr Leyonhjelm had tweeted a photograph of horse ivermectin alongside the following: "In several countries, ivermectin is being used to treat COVID. For those who want some, this horse product is probably the most economical source. Enough to treat the street too." [257]. The President of the American Association of Poison Control Centers also indicated that there had been a recent surge in calls regarding ivermectin, including one case where someone had taken a preparation of the drug intended for horses [245].
Veterinary pharmaceuticals cont.	Ivermectin cont.	On a webpage titled 'Why You Should Not Use Ivermectin to Treat or Prevent COVID-19', the US FDA also indicated that they had been notified of multiple cases where people required medical attention after using horse preparations of ivermectin [258].
	Unspecified cattle deworming medication	In June 2020, six people from Estcourt, South Africa, were hospitalised after consuming what was believed to be cattle deworming medication. Reports suggest that the people were told the drug would help cure COVID-19. [259].

Fraudulent tests and treatments	Virus Shut out or Air Doctor	In April 2020, evidence emerged that lanyards which had a card attachment treated with chloride dioxide were being promoted as a means to prevent the wearer from contracting COVID-19. The US Environmental Protection Agency (EPA) threatened to pursue legal action against a number of fraudulent COVID-19 products, including these lanyards [260]. A Kremlin spokesperson was also seen with such a card pinned to his jacket in a televised conference, and a media report suggested that online markets in Russia were soon sold out of similar stock [261]. Senior officials in South Sudan, including the President, were also seen wearing these cards in photos posted to Facebook by the President's communications team [262]. In July 2020, Jeanine Añez, Bolivia's interim President, was also seen to be wearing one of the 'virus blocker' lanyards [263].<br><br>In June 2020, the US EPA urged two major online retailers, Amazon and EBay, to stop selling the cards amongst other unproven disinfectants [264]. In Sydney, Australia, at least three children were reportedly sent to pre-schools wearing chlorine-dioxide lanyards after schools reopened following lockdown measures [265].
	Eucalyptus-based virus blocker	In July 2020, authorities in Indonesia started promoting a eucalyptus-containing lanyard or necklace that seemed to be reminiscent of the chlorine dioxide-based products being purchased or worn in other countries. The Minister for Agriculture suggested that the eucalyptus-based products, which extended to roll-ons and inhalers, were for aromatherapy to alleviate respiratory symptoms of COVID-19 [266]. However, he also suggested to reporters that the necklace was 80% effective in inactivating COVID-19 viral particles within half an hour [266]. His claims were disputed by health experts and the laboratory that had developed the product [267]. Celebrities were being called on to help promote Indonesian health campaigns and one such celebrity, Iis Dahlia, shared with her 12 million Instagram followers that she proudly wore the eucalyptus-based lanyard [267].
	Fraudulent tests and treatments general	In April 2020, a British man was accused of smuggling fake COVID-19 treatment kits into the United States. The kits were thought to contain potassium thiocyanate and hydrogen peroxide, with consumers advised to rinse their mouths with the chemicals [268]. The medical devices regulator in the United Kingdom announced that they were investigating 14 cases of fraudulent and unlicensed products marketed as COVID-19 treatments [269].<br><br>In June, US ICE announced they had seized over 14,000 unproven COVID-19 treatments capsules as part of Operation Stolen Promise, a task force set up to investigate the sale of counterfeit or substandard products including personal protective equipment and pharmaceuticals [270]. In December 2020, Europol also announced that predictions they had made in April around the potential harm of COVID-related scams and fraudulent treatments had been proven correct. Europol had found ongoing evidence of criminal activity related to the distribution of counterfeit pharmaceutical products and fraudulent treatments [271]. In January 2021, the Myanmar government

Fraudulent tests and treatments cont.	Fraudulent tests and treatments general cont.	also announced that it was mounting a response to the sale of fraudulent vaccines and pharmaceuticals being sold on social media [272].<br><br>In January 2021, police in London announced they were looking for a man who had injected a 92-year-old woman with a fraudulent vaccine under the false pretence that he worked for the National Health Service [273]. Fortunately, the woman did not suffer from any adverse health consequences. In the United States, a man from Washington state was also arrested on a federal warrant in January for the sale and administration of fraudulent COVID-19 vaccines. Johnny T. Stine told undercover investigators that he had travelled across the United States to administer his vaccine, ignoring a cease and desist letter sent to him by the Washington State Attorney General in April 2020 [274]. In February 2021, ICE announced that Operation Stolen Promise would be extended to capture emerging COVID-related fraud around vaccines and unlicensed treatments [275].
Alcohols	Methanol	In March 2020, reports from South Korea indicated a woman from Gyeonggi Province had started cleaning her home with a highly concentrated methanol solution in order to prevent COVID-19 infection. The woman and her two children were later hospitalised with symptoms of methanol intoxication [276]. Military hospitals in North Korea were also reported to be disinfecting quarantined areas by spraying methanol solution, though North Korea had not officially registered a single case COVID-19 over the course of the study period [277].<br><br>In February 2021, researchers in Spain published a report detailing a case involving a woman who had developed severe, chronic methanol poisoning after using a highly concentrated methanol solution to routinely clean her face mask for two months. The 56-year-old patient presented with a four-week history of insomnia, cognitive decline, and parkinsonism. Magnetic resonance imaging (MRI) demonstrated she had acquired severe central nervous system damage. At a four month follow up the patient had shown signs of improvement, but still suffered from cognitive impairment, parkinsonism and impaired motor function [278].
	Ethanol-based drinks	A number of high-profile celebrities and leaders around the world directly or indirectly endorsed misinformation that drinking alcohol could prevent or treat COVID-19. In March 2020, President Lukashenko of Belarus suggested that people should drink vodka and take saunas to ward off COVID-19 [279]. In April, a governor from Kenya who suggested alcohol could prevent COVID-19 infections was criticised for distributing bottles of cognac in coronavirus care packages [280]. Professional golfer, John Daly, joked that he was warding off coronavirus with vodka, Diet Coke and cigarettes in a video that was distributed to members of Trump golf clubs around the world [281]. In May, health authorities in Canada reported they were working to counter misinformation that alcohol 'kills' COVID-19 [282]. In June, officials in Ghana also reported they were increasing efforts to fight rumours that locally-produced gin (Akpeteshie) could protect or cure consumers from COVID-19 [283]. Videos of a

Alcohols cont.	Ethanol-based drinks cont.	councillor on the Ullal City Municipal Council in India also circulated on social media in July 2020, where the councillor suggested a particular brand of rum was 'medicine' for coronavirus [284].<br><br>Over the course of the research period, media articles and studies suggested that the COVID-19 pandemic and associated policy measures were changing regular and problem drinking patterns. In 2020, reports from Canada [282,285], Belgium [70,286], Australia [287], New Zealand [288], the United Kingdom [289-293] and the United States [23,294-297] indicated that problem drinking had increased due to lockdown measures and associated feelings including boredom and stress. Experts from England and Scotland warned those with a history of problem drinking and associated medical conditions may suffer serious health consequences. These experts were concerned that, on top of pre-existing cuts to baseline levels of addiction services, there was less access to social and professional support for addiction under social distancing measures and that those who need help may be avoiding hospitals for fear of contracting the virus [298,299].<br><br>In February 2021, data published by the Office of National Statistics in the United Kingdom indicated that alcohol-related deaths between January and September of the previous year were 16% higher than in 2019 [291]. Data showed that alcohol intake had actually remained stable across the population, with many people decreasing their intake under lockdown measures [292,293]. However, alcohol purchase had spiked immediately prior to the first and second national lockdown [292]. There was also an increase in the proportion of 'increasing and higher risk' drinkers amongst the population [292]. This trend was reflected in reports from the UK and USA which indicated a surge in alcohol-related referrals, relapses and hospitalisations for alcoholic liver disease [293,295,300,301].
	Bootleg, low quality or home-brewed alcohol (often methanol-based)	In March of 2020, reports emerged suggesting people were consuming bootleg or homebrewed alcohol in order to ward off coronavirus, which resulted in poisoning. In Istanbul, 30 people died and another 20 were hospitalised after allegedly drinking pure ethanol to protect themselves from COVID-19 infections [302]. An article from *The Associated Press* published by several outlets also reported a link between a Welsh teacher who contracted COVID-19 in Wuhan and hundreds of methanol poisonings in Iran [303]. Twenty-five-year-old Connor Reed had told tabloids in the United Kingdom that upon contracting COVID-19 he had refused antibiotics and treated his symptoms with hot toddies: a hot drink typically made with whiskey, honey, lemon and cinnamon. His comments were picked up by press in the US and details of his story started circulating in Farsi on Iranian social media [304,305]. The *Associated Press* article suggests these posts, alongside government messages about using alcohol-based hand sanitiser, developed into widespread misinformation that consuming high-proof alcohol could prevent or treat COVID-19 [303]. With alcohol consumption outlawed in Iran, people turned to illicit sources and

Alcohols cont.	Bootleg, low quality or home-brewed alcohol (often methanol-based) cont.	thousands of methanol poisonings started occurring across the country [306]. In a submission to the *Archives of Toxicology,* Iranian toxicologists indicated that by the 20th of April, 2020, there had been approximately 700 deaths and 3100 hospitalisations in Iran resulting from COVID-related methanol poisoning [307].<br><br>In May 2020, reports from Mexico started emerging around deaths associated with cheap, low-quality alcoholic beverages [308]. Beer production had been classed as a as a non-essential activity and halted for over a month under lockdown measures [309,310]. In some states such as Yucatán, the sale of alcohol was also banned or could only be purchased at particular times [310]. These measures brought about a surge in bootleg alcohol sales which were linked to the deaths of at least 100 people by mid-May [310]. The economic impact of lockdown measures was also thought to have been a factor, as decreased purchasing power meant people bought cheaper, lower quality alcohol [309]. In June, another 18 deaths occurred in the state of Guerrero after people consumed a little-known, tequila-type drink called 'Rancho Escondido' [308].<br><br>On the 23rd of March 2020, alcohol purchase was banned in South Africa under lockdown measures in order to decrease the burden of alcohol-related injuries and violence on the healthcare sector. These lockdown measures were extended and in May two South African couples died from drinking homebrewed alcohol in separate incidents [311,312]. Then, on May 31st, the day before the measures were set to ease, 20 people were hospitalised with alcohol poisoning in Masiphumelele after attending a party. Of the 20 people hospitalised, 7 people had died as a result of the exposure [313]. On the 1st of June the alcohol ban was lifted and there were large queues to purchase alcohol outside of retailers [314]. The alcohol ban would be brought back into effect in July and slowly lifted again from August. The alcohol ban was then put back in place in December as South Africa battled a second wave of COVID-19 infections. Following the ingestion of homemade alcohol on New Year's Eve, two young women died and another six were hospitalised in Free State [315].<br><br>In October 2020, a spate of 63 bootleg alcohol-related deaths occurred in Turkey across 10 provinces. An unknown number of others were left with other injuries including blindness [316]. A leading expert in methanol alcohol poisoning, Dr. Knut Erik Hovda of Oslo University Hospital, was quoted in the media. Dr Hovda suggested that COVID-19 had compounded the problem of illicit alcohol in 2020. He suggested many people were drinking recreationally at home while under lockdown, but that associated economic impacts had also pushed people towards cheaper alternatives or to attempt making their own alcohol [316]. Dr Hovda said that existing illicit alcohol networks had also been impacted by the pandemic through disrupted supply routes [316].

		In February 2021, consumption of bootleg and low-quality alcohol was linked to 35 deaths in Bangladesh [317]. Venue owners quoted in the media said it became more difficult to import high-quality liquor and many venues had to source locally-produced alternatives [317]. In March 20201, officials in Cameroon also warned that the economic impacts of COVID-19 were contributing to an increase in excessive drinking, bootleg alcohol deaths and liver injuries, particularly as a result of fake whiskies [318].
Unspecified	Self-harm with unknown substance	Between June and August of 2020, a number of articles from India reported suicides that were attributed to COVID-related stressors. Most of these stressors were financial in origin, including job loss, unemployment and family conflict that resulted from unemployment [319-322]. An increase in suicides amongst daily workers, the unemployed and those suffering from domestic violence was also reported in Tanahun, Nepal, including three by poison [323]. Between January and July 2020, suicides in Iraq were also reported to have increased with ongoing mental health, social and economic issues compounded by the COVID-19 pandemic and associated lockdown measures [324].<br><br>However, some cases of self-harm using toxic substances in India were adversely impacted by or stemmed from COVID-related concerns in other ways. In June, one woman who had ingested a toxic substance was turned away from a hospital in Kupwara as it had been designated as a COVID-19 hospital. Her family attempted to take her to an alternate hospital only to have to return to the first when she was also refused admission there. The family indicated that shifting between hospitals delayed treatment for more than an hour, and the woman died as a result of her exposure [325]. In another two instances, the people who had self-harmed had done so out of fear of contracting COVID-19, or out of fear that they already had and may transmit it to their grandchildren [326,327]. In August, another report suggested that a 65-year-old man who had recently accompanied a relative to an urban hospital had returned to his village to find other inhabitants were concerned that on the trip he may have contracted COVID-19 [328]. The man began isolating himself of the outskirts of the village and received and COVID-19 test, which was negative. However, the test results allegedly did not ease his concerns and he consumed poison. His son rushed him to hospital, though after testing positive for COVID-19 while admitted he was eventually transferred to a COVID hospital in Chikkabballapura. Unfortunately, the man was still presumably in distress; he exited the hospital and was later found having hung himself from a tree in a nearby town [328].
	Alleged poisoning of others or other criminal activity with unknown substance	In May 2020, a man in Delhi hired two women to go to the home of a man he suspected was having an affair with his wife and pretend to be health workers. The women were granted entry to the home and administered what they claimed to be COVID-19 medicine to the man and three other family members, who were later hospitalised [329].

Unspecified cont.		On the 24th of May 2020, at least twenty people in Gikambura, Kenya, were hospitalised after consuming food aid suspected to have been laced with a toxic substance [330]. The food had been distributed in bags branded with the names of Deputy President, William Ruto, and Kikuyu Member for Parliament, Kimani Ichung'wah, who had visited the area to distribute COVID-19 food aid a few days prior. However, the Deputy President indicated that he was not associated with the second delivery of food aid and a spokesperson later suggested that it may have been a politically motivated attack [330].<br><br>In July 2020, a man from Karnataka, India, who was scared he may lose his job in COVID-related lay-offs was alleged to have undertaken a murder/suicide when he poisoned himself, his wife and daughter [331].

References

1. Cicala, P. Household poisoning cases up in Tucson during COVID-19 pandemic, more calls to AZ Poison & Drug Info Center. *News4 Tuscon* 2020 Available online: https://kvoa.com/news/top-stories/2020/04/28/household-poisoning-up-in-tucson-with-covid-19-more-calls-to-az-poison-drug-info-center/ (accessed on 23 March 2021).
2. Bateman, M. A global mask shortage may leave farmers and farm workers exposed to toxic pesticides. *The Conversation* 2020 Available online: https://theconversation.com/a-global-mask-shortage-may-leave-farmers-and-farm-workers-exposed-to-toxic-pesticides-134976 (accessed on 23 March 2021).
3. Williams, P. Tennessee governor's free 'sock masks' treated with registered pesticide. *News Channel 5 Nashville* 2020 Available online: www.newschannel5.com/news/newschannel-5-investigates/tennessee-governors-free-sock-masks-treated-with-registered-pesticide (accessed on 6 April 2021).
4. Sharp, M. Midlothian council workers went on a 'killing mission' with banned poison, claims member. *Edinburgh Evening News* 2021 Available online: www.edinburghnews.scotsman.com/news/environment/midlothian-council-workers-went-on-a-killing-mission-with-banned-poison-claims-member-3177483 (accessed on 23 April 2021).
5. Tanakasempipat, P.; Sriring, O. Public suicide bid as millions of Thais wait for virus aid. *Reuters* 2020 Available online: www.reuters.com/article/uk-health-coronavirus-thailand-aid-idUKKCN22A2K3 (accessed on 28 April 2021).
6. Suicide leading cause for over 300 lockdown deaths in India, says study. *The Economic Times* 2020 Available online: https://economictimes.indiatimes.com/news/politics-and-nation/suicide-leading-cause-for-over-300-lockdown-deaths-in-india-says-study/articleshow/75519279.cms (accessed on 24 March 2021).
7. Boden, S. Hand-Sanitizer Exposures Up At Pittsburgh, Philly Poison Control Centers. *Pittburg NPR News Station* 2020 Available online: www.wesa.fm/post/hand-sanitizer-exposures-pittsburgh-philly-poison-control-centers#stream/0 (accessed on 14 March 2021).
8. Hart, K. Poison Control sees spike in calls related to hand sanitizer exposure. *Axios* 2020 Available online: www.axios.com/hand-sanitizer-coronavirus-poison-control-ffb34b62-f89f-4a63-be8c-849a13cf0961.html (accessed on 14 March 2021).
9. Bethencourt, A. El Paso poison control calls increase by 60% amid COVID-19 outbreak. *CBS 4 Local* 2020 Available online: https://cbs4local.com/news/local/el-paso-poison-control-calls-increase-by-60-amid-covid-19-outbreak (accessed on 23 March 2021).
10. Beg, K. Michigan Poison Center: Calls about disinfectant, bleach rise dramatically amid pandemic. *Lansing State Journal* 2020 Available online: https://eu.lansingstatejournal.com/story/news/2020/04/28/michigan-poison-bleach-disinfectant-sanitizer-cleaning-trump/3034686001/ (accessed on 23 March 2021).
11. Chang, A.; Schnall, A.H.; Law, R.; Bronstein, A.C.; Marraffa, J.M.; Spiller, H.A.; Hays, H.L.; Funk, A.R.; Mercurio-Zappala, M.; Calello, D.P.; et al. *Cleaning and Disinfectant Chemical Exposures and Temporal Associations with COVID-19 — National Poison Data System, United States, January 1, 2020–March 31, 2020*; U.S. Department of Health and Human Services: 24 April 2020 2020; pp. 496–498.
12. National Poison Data System (NPDS) Bulletin COVID-19 (Hand Sanitizer) (Jan - May). *American Association of Poison Control Centres* 2020 Available online: https://piper.filecamp.com/uniq/jETWSFZpqHKVSuC8.pdf (accessed on 5 April 2021).
13. Forani, J.; MacLeod, M. Many more Canadians are accidentally poisoning themselves with cleaning products, Health Canada reports. *CTV News* 2020 Available online: www.ctvnews.ca/health/many-more-canadians-are-accidentally-poisoning-themselves-with-cleaning-products-health-canada-reports-1.4926652 (accessed on 24 March 2021).
14. Hand sanitiser safety and children. *New South Wales Health* Available online: www.health.nsw.gov.au/news/Pages/20200412_01.aspx (accessed on 23 March 2021).
15. Binder, L.; Högenauer, C.; Langner, C. Gastrointestinal effects of an attempt to avoid contracting COVID-19 by 'disinfection'. *Histopathology* **2020**, *77*, 327-328, doi:10.1111/his.14137.
16. Hanna, S.; Zwi, K.; Tzioumi, D. Morbidity in the COVID-19 era: Ethanol intoxication secondary to hand sanitiser ingestion. *Journal of Paediatrics and Child Health* **2020**, doi:https://doi.org/10.1111/jpc.15017.

17. Pilat, L. Child hospitalised after sanitising, then licking hands at WA school. *WA Today* 2020 Available online: www.watoday.com.au/national/western-australia/five-year-old-hospitalised-after-using-sanitiser-at-wa-school-then-licking-hands-20200531-p54y41.html (accessed on 6 April 2021).
18. Badam, R.T. Boy, 6, blind in one eye after hand sanitiser accident in Ajman. *The National News* 2020 Available online: www.thenationalnews.com/uae/boy-6-blind-in-one-eye-after-hand-sanitiser-accident-in-ajman-1.477533 (accessed on 8 April 2021).
19. Vesty, S. Mum fears son, 3, is partially blind after he's squirted in eye with hand sanitiser. *Mirror UK* 2020 Available online: www.mirror.co.uk/news/uk-news/mum-fears-son-3-partially-22438878 (accessed on 9 April 2021).
20. Hand sanitiser: take care to protect young children from accidentally splashing their eyes. *Anses* 2020 Available online: www.anses.fr/en/content/hand-sanitiser-take-care-protect-young-children-accidentally-splashing-their-eyes-0 (accessed on 12 April 2021).
21. Martin, G.C.; Le Roux, G.; Guindolet, D.; Boulanger, E.; Hasle, D.; Morin, E.; Vodovar, D.; Vignal, C.; Gabison, E.; Descatha, A.; et al. Pediatric Eye Injuries by Hydroalcoholic Gel in the Context of the Coronavirus Disease 2019 Pandemic. *JAMA Ophthalmology* **2021**, *139*, 348-351, doi:10.1001/jamaophthalmol.2020.6346 %J JAMA Ophthalmology.
22. Baucum, E. Poison control center fielding calls about drinking bleach, kids eating hand sanitizer. *News4 San Antonio* 2020 Available online: https://news4sanantonio.com/news/local/poison-control-center-fielding-calls-about-drinking-bleach-kids-eating-hand-sanitizer (accessed on 12 April 2021).
23. Upstate New York Poison Center sees increase in hand sanitizer, bleach calls due to COVID-19. *Upstate New York Poisons Center* 2021 Available online: www.upstate.edu/poison/news/top-poisonings-2020.php (accessed on 22 April 2021).
24. Desjardins, L. Pandemic posing increased poison risks for children. *Radio Canada International* 2021 Available online: www.rcinet.ca/en/2021/03/20/pandemic-posing-increased-poison-risks-for-children/ (accessed on 26 April 2021).
25. Ramsay, C. COVID-19: unintended poisonings from hand sanitizer, cleaning products increase 73% in Alberta. *Global News* 2021 Available online: https://globalnews.ca/news/7711132/alberta-covid-19-hand-sanitizer-cleaning-product-poisonings/ (accessed on 26 April 2021).
26. Carter, C. Banner Poison Center sees 140% increase in hand sanitizer incidents amid pandemic. *ABC 15 Arizona* 2021 Available online: www.abc15.com/news/coronavirus/banner-poison-center-sees-140-increase-in-hand-sanitizer-incidents-in-2020-during-covid-19-pandemic (accessed on 26 April 2021).
27. Pandey, M.C. Highly toxic, dangerous: CBI warns against fake methanol-based hand sanitisers flooding the market. *India Today* 2020 Available online: www.indiatoday.in/india/story/highly-toxic-dangerous-cbi-warns-against-fake-methanol-based-hand-sanitisers-flooding-the-markey-1689312-2020-06-15 (accessed on 7 April 2021).
28. India: 9 die drinking sanitizer amid coronavirus alcohol ban. *Deutsche Welle* 2020 Available online: www.dw.com/en/india-9-die-drinking-sanitizer-amid-coronavirus-alcohol-ban/a-54395551 (accessed on 10 April 2021).
29. Andhra Pradesh: Four more die, 20 hospitalised after drinking sanitiser in Kurichedu. *The New Indian Express* 2020 Available online: www.newindianexpress.com/states/andhra-pradesh/2020/aug/02/andhra-pradesh-four-more-die-20-hospitalised-after-drinking-sanitiser-in-kurichedu-2178024.html (accessed on 10 April 2021).
30. Mauch, A. 3 Dead in New Mexico After Drinking Hand Sanitizer Containing Methanol. *People* 2020 Available online: https://people.com/health/three-dead-in-new-mexico-after-drinking-hand-sanitizer-containing-methanol/ (accessed on 8 April 2021).
31. Encinas, C. Poison center sees spike in alcohol poisoning related to hand sanitizer. *KGUN 9 Tucson* 2020 Available online: www.kgun9.com/news/coronavirus/poison-center-sees-spike-in-alcohol-poisoning-related-to-hand-sanitizer (accessed on 8 April 2021).
32. Bhopal: Three brothers drink sanitiser in absence of alcohol, die. *The Free Press Journal* 2021 Available online: www.freepressjournal.in/bhopal/bhopal-three-brothers-drink-sanitiser-in-absence-of-alcohol-die (accessed on 23 April 2021).

33. Yip, L.; Bixler, D.; Brooks, D.E.; Clarke, K.R.; Datta, S.D.; Jr., S.D.; Komatsu, K.K.; Lind, J.N.; Mayette, A.; Melgar, M.; et al. *Serious Adverse Health Events, Including Death, Associated with Ingesting Alcohol-Based Hand Sanitizers Containing Methanol — Arizona and New Mexico, May–June 2020*; 14 August 2020 2020; pp. 1070–1073.
34. Fazio, M. 3 Die in New Mexico After Drinking Hand Sanitizer, Officials Say. *The New York Times* 2020 Available online: www.nytimes.com/2020/06/26/us/3-dead-drinking-hand-sanitizer.html (accessed on 8 April 2021).
35. FDA advises consumers not to use hand sanitizer products manufactured by Eskbiochem. *US Food & Drug Administration* 2020 Available online: www.fda.gov/drugs/drug-safety-and-availability/fda-advises-consumers-not-use-hand-sanitizer-products-manufactured-eskbiochem#:~:text=%5B6%2F19%2F2020%5D,through%20the%20skin%20or%20ingested. (accessed on 19 April 2021).
36. Coronavirus (COVID-19) Update: FDA Takes Action to Warn, Protect Consumers from Dangerous Alcohol-Based Hand Sanitizers Containing Methanol. *US Food & Drug Administration* 2020 Available online: www.fda.gov/news-events/press-announcements/coronavirus-covid-19-update-fda-takes-action-warn-protect-consumers-dangerous-alcohol-based-hand (accessed on 8 April 2020).
37. Fugleberg, J. 1 dead in South Dakota from drinking hand sanitizer containing methanol. *West Central Tribune* 2020 Available online: www.wctrib.com/newsmd/health-news/6598093-1-dead-in-South-Dakota-from-drinking-hand-sanitizer-containing-methanol (accessed on 19 April 2021).
38. FDA Lists More Than 100 Potentially Toxic Hand Sanitizers After Latest Recall. *CBS San Francisco Bay Area* 2020 Available online: https://sanfrancisco.cbslocal.com/2020/08/03/fda-lists-more-than-100-potentially-toxic-hand-sanitizers-after-latest-recall/ (accessed on 10 April 2021).
39. Withdrawal of hand sanitiser Virapro from the market. *Irish Department of Agriculture, Food and the Marine* 2020 Available online: www.gov.ie/en/press-release/712d8-withdrawal-of-hand-sanitiser-virapro-from-the-market/ (accessed on 21 April 2021).
40. Trading Standards Institute Advice, Recall: Ibcccndc - Hand Sanitiser. *Bromley Borough* 2021 Available online: www.bromley.gov.uk/leaflet/329707/22/680/d (accessed on 22 April 2021).
41. Coronavirus (COVID-19) Update: FDA Takes Action to Place All Alcohol-Based Hand Sanitizers from Mexico on Import Alert to Help Prevent Entry of Violative and Potentially Dangerous Products into U.S., Protect U.S. Consumers. *US Food & Drug Administration* 2021 Available online: www.fda.gov/news-events/press-announcements/coronavirus-covid-19-update-fda-takes-action-place-all-alcohol-based-hand-sanitizers-mexico-import (accessed on 22 April 2021).
42. 7 Die From Drinking Hand Sanitizer at Party in Russia. *The Moscow Times* 2020 Available online: www.themoscowtimes.com/2020/11/23/7-die-from-drinking-hand-sanitizer-at-dinner-party-in-russia-a72119 (accessed on 21 April 2021).
43. Coronavirus (COVID-19) Update: FDA Continues to Ensure Availability of Alcohol-Based Hand Sanitizer During the COVID-19 Pandemic, Addresses Safety Concerns. *US Food & Drug Administration* 2020 Available online: www.fda.gov/news-events/press-announcements/coronavirus-covid-19-update-fda-continues-ensure-availability-alcohol-based-hand-sanitizer-during (accessed on
44. Powell, T. FDA warns of hand sanitizer packaged in food and beverages containers. *ABC 11 Eyewitness News* 2020 Available online: https://abc11.com/health/fda-warns-of-hand-sanitizer-packaged-in-food-beverage-containers/6392591/ (accessed on 12 April 2021).
45. Parsons, L. Secret killer of the pandemic panic: Children are being POISONED after accidentally drinking hand sanitiser. *The Daily Mail* 2020 Available online: www.dailymail.co.uk/news/article-8320015/Children-POISONED-accidentally-drinking-hand-sanitiser.html (accessed on 5 April 2021).
46. Hall, M. Poison control sees spike in calls of children, adults ingesting hand sanitizer. *City News 1130* 2020 Available online: www.citynews1130.com/2020/05/25/hand-sanitizer-calls-spike/ (accessed on 5 April 2021).
47. Levasseur, J. Hand sanitizers packaged in beverage containers create alcohol poisoning risk: experts. *Canadian Broadcasting Corporation (CBC)* 2020 Available online: www.cbc.ca/news/canada/manitoba/hand-sanitizer-alcohol-poisoning-risk-1.5590641 (accessed on 6 April 2021).

48. Health Canada. Advisory - Risk of poisoning from hand sanitizers sold in beverage containers. *Cision* Available online: www.newswire.ca/news-releases/advisory-risk-of-poisoning-from-hand-sanitizers-sold-in-beverage-containers-819473509.html (accessed on 7 April 2021).
49. Urgent recall after hand sanitiser sold as gin. *The New Daily* 2020 Available online: https://thenewdaily.com.au/finance/consumer/2020/06/09/apollo-bay-distillery-gin-sanitiser/ (accessed on 7 April 2021).
50. Kindelan, K. Mom warns other parents after son eats hand sanitizer packaged in Trolls pouch. *Good Morning America* 2020 Available online: www.goodmorningamerica.com/wellness/story/mom-warns-parents-son-eats-hand-sanitizer-packaged-72978670 (accessed on 21 May 2021).
51. Haynes, D. Coronavirus: Insect repellent to be tested by MoD lab to see if it kills COVID-19. *Sky News* 2020 Available online: https://news.sky.com/story/coronavirus-mod-lab-to-test-whether-insect-repellent-ingredient-kills-covid-19-11980303 (accessed on 23 March 2021).
52. Williams, T.-A. Army personnel given insect repellant 'to help protect them from coronavirus'. *The Sun* 2020 Available online: www.thesun.co.uk/news/health-news/11613072/insect-repellent-protect-coronavirus-mod-thinks-maybe/ (accessed on 5 April 2021).
53. Dstl Porton Down. Experimental survival of SARS-CoV-2 on an insect-repellent-treated surface. *UK Ministry of Defence* 2020 Available online: www.gov.uk/government/publications/experimental-survival-of-sars-cov-2-on-an-insect-repellent-treated-surface--3 (accessed on 16 April 2021).
54. Sparks, H. Insect repellent company warns: Don't use our products to kill COVID-19. *The New York Post* 2020 Available online: https://nypost.com/2020/08/28/insect-repellent-boss-dont-use-our-products-to-kill-covid-19/ (accessed on 12 April 2021).
55. National Poison Data System (NPDS) Bulletin COVID-19 (Disinfectant) (Jan - May). *American Association of Poison Control Centers* 2020 Available online: https://piper.filecamp.com/uniq/ezhi8DGXB4Kl25dJ.pdf (accessed on 5 April 2021).
56. COVID-19 pandemic causes surge in cleaning product exposures. *American Broadcasting Company (ABC)* 2020 Available online: https://newschannel20.com/news/local/covid-19-pandemic-causes-surge-in-cleaning-product-exposures (accessed on 15 March 2021).
57. Torres, M. CA Poison Control reports increase in calls for exposure to disinfectants and cleaners. *KSBY News* 2020 Available online: www.ksby.com/news/coronavirus/ca-poison-control-reports-increase-in-calls-for-exposure-to-disinfectants-and-cleaners (accessed on 22 March 2021).
58. Tyree, E. Don't drink bleach to prevent coronavirus, poison control center says. *American Broadcasting Company (ABC)* 2020 Available online: https://wset.com/news/coronavirus/dont-drink-bleach-to-prevent-coronavirus-poison-control-center-says (accessed on 14 March 2021).
59. Mohrmann, J. By-the-numbers: Calls to Florida Poison Control increase as the coronavirus spreads. *News4Jax* 2020 Available online: www.news4jax.com/news/local/2020/03/18/by-the-numbers-calls-to-florida-poison-control-increase-as-the-coronavirus-spreads/ (accessed on 14 March 2021).
60. McKinney, D. Illinois' COVID-19 Testing Sets A New Daily 'Milestone. *WBEZ Chicago* 2020 Available online: www.wbez.org/stories/illinois-covid-19-testing-sets-new-daily-milestone/b8c20556-53e3-42a9-af0f-f4ede8c14025 (accessed on 22 March 2021).
61. Glatter, R. Calls To Poison Centers Spike After The President's Comments About Using Disinfectants To Treat Coronavirus. *Forbes* 2020 Available online: www.forbes.com/sites/robertglatter/2020/04/25/calls-to-poison-centers-spike--after-the-presidents-comments-about-using-disinfectants-to-treat-coronavirus/#7cf5ce911157 (accessed on 22 March 2021).
62. Elliot, J.K. Poison control calls spike after Trump talks of disinfectant to fight coronavirus. *Global News Canada* 2020 Available online: https://globalnews.ca/news/6871768/coronavirus-donald-trump-disinfectant-poison/ (accessed on 22 March 2021).
63. Sheets, M. Two men in Georgia and one man in Kansas drank liquid cleaning products 'to ward off COVID-19' after Trump suggested injecting disinfectant into the lungs to combat the virus. *The Daily Mail* 2020 Available online: www.dailymail.co.uk/news/article-8265665/Kansas-man-drank-cleaning-product-Trump-suggested-injecting-disinfectant-fight-coronavirus.html (accessed on 23 March 2021).

64. Collins, L. North Texas Poison Center Sees Increased Calls. *NBC Dallas-Fort Worth* 2020 Available online: www.nbcdfw.com/news/coronavirus/north-texas-poison-center-sees-increased-calls/2360775/ (accessed on 23 March 2021).
65. Beausoleil, S. North Texas Poison Center Reminds People Again Not to Ingest Cleaning Products. *NBC Dallas-Fort Worth* 2020 Available online: www.insider.com/texans-drink-bleach-poison-control-warns-wont-cure-coronavirus-2020-8 (accessed on 12 April 2021).
66. Raddatz, K. Coronavirus In Minnesota: Poison Control Officials Report An Uptick In Calls Amid COVID-19 Outbreak. *CBS Minnesota* 2020 Available online: https://minnesota.cbslocal.com/2020/04/29/coronavirus-in-minnesota-poison-control-officials-report-an-uptick-in-calls-amid-covid-19-outbreak/ (accessed on 23 March 2021).
67. Wadhwani, A. B.C. poison control officials see spike in disinfectant exposure calls amid COVID-19. *Victoria News* 2020 Available online: www.vicnews.com/news/b-c-poison-control-officials-see-spike-in-disinfectant-exposure-calls-amid-covid-19/ (accessed on 23 March 2021).
68. Henderson, C. Cristina Cuomo says she treated her coronavirus with Clorox baths, vitamin drips: Experts react. *USA Today* 2020 Available online: https://eu.usatoday.com/story/entertainment/celebrities/2020/04/24/coronavirus-experts-warn-against-cristina-cuomos-bleach-baths-methods/3017294001/ (accessed on 22 March 2021).
69. Cardona, A.C. After Trump's Remarks, Floridians Ask Poison Control If Drinking Disinfectant Is Safe. *Miami New Times* 2020 Available online: www.miaminewtimes.com/news/florida-poison-control-calls-increase-after-trump-comments-11629946 (accessed on 22 March 2021).
70. Brzozowski, A. Number of bleach-related incidents up in Belgium due to COVID-19 fears. *Euractiv* 2020 Available online: www.euractiv.com/section/health-consumers/news/number-of-bleach-related-incidents-up-in-belgium-due-to-covid-19-fears (accessed on 5 April 2021).
71. Gharpure, R.; Hunter, C.M.; Schnall, A.H.; Barrett, C.E.; Kirby, A.E.; Kunz, J.; Berling, K.; Mercante, J.W.; Murphy, J.L.; Garcia-Williams, A.G. Knowledge and Practices Regarding Safe Household Cleaning and Disinfection for COVID-19 Prevention — United States, May. *MMWR Morb Mortal Wkly Rep* **2020**, *69*, 705-709, doi:10.15585/mmwr.mm6923e2.
72. Pari, U. Calls to South Texas Poison Control Center spike amid coronavirus pandemic. *KSAT* 2020 Available online: www.ksat.com/health/2020/07/22/calls-to-south-texas-poison-control-center-spike-amid-coronavirus-pandemic/ (accessed on 8 April 2021).
73. Advisory - Reduce your risk from toxic gases: Never mix bleach with other cleaning products. *Health Canada* 2021 Available online: www.newswire.ca/news-releases/advisory-reduce-your-risk-from-toxic-gases-never-mix-bleach-with-other-cleaning-products-802093096.html (accessed on 22 April 2021).
74. Vigdor, N. Man Fatally Poisons Himself While Self-Medicating for Coronavirus, Doctor Says. *The New York Times* 2020 Available online: www.nytimes.com/2020/03/24/us/chloroquine-poisoning-coronavirus.html (accessed on 14 March 2021).
75. Flaherty, A.; Phelps, J. Fauci throws cold water on Trump's declaration that malaria drug chloroquine is a 'game changer'. *American Broadcasting Company (ABC)* 2020 Available online: https://abcnews.go.com/Politics/fauci-throws-cold-water-trumps-declaration-malaria-drug/story?id=69716324 (accessed on 16 April 2021).
76. MacLean, A. Dangerous exposure to pool chemicals leads to jump in IWK Poison Centre calls. *Global News Canada* 2020 Available online: https://globalnews.ca/news/7113163/pool-chemicals-iwk-poison-calls/ (accessed on 7 April 2021).
77. Bartington, S.; Pinchoff, J.; Avis, W.R. COVID-19: a new challenge for clean cooking progress in Kenya. *The Conversation* 2021 Available online: https://theconversation.com/covid-19-a-new-challenge-for-clean-cooking-progress-in-kenya-155900 (accessed on 23 April 2021).
78. Hafezi, P. Fear, distrust and disinfectant in the air amid Iran's coronavirus outbreak. *Reuters* 2020 Available online: www.reuters.com/article/us-china-health-iran-mood/fear-distrust-and-disinfectant-in-the-air-amid-irans-coronavirus-outbreak-idUSKBN20O1X1 (accessed on 14 March 2021).
79. Secon, H. Photos show how people around the world are disinfecting schools, mosques, and streets to stop the coronavirus from spreading. *Business Insider* 2020 Available online:

www.businessinsider.com/coronavirus-sanitation-disinfection-around-the-world-in-photos-2020-3?r=US&IR=T (accessed on 14 March 2021).
80. Bricknell, L.; Trott, D. Sanitising the city: does spraying the streets work against coronavirus? *The Conversation* 2020 Available online: https://theconversation.com/sanitising-the-city-does-spraying-the-streets-work-against-coronavirus-136966 (accessed on 20 April 2021).
81. Brazilian Red Cross Workers Disinfect Streets Amid Growing Coronavirus Cases. *The Herald Sun* 2020 Available online: www.heraldsun.com.au/news/national/brazilian-red-cross-workers-disinfect-streets-amid-growing-coronavirus-cases/video/2a2d4224f6a81c5cd56e952b13892711 (accessed on 20 April 2021).
82. Jackson, T. Nigerian startup Beat Drone spraying disinfectant to fight COVID-19. *Disrupt Africa* 2020 Available online: https://disrupt-africa.com/2020/04/20/nigerian-startup-beat-drone-spraying-disinfectant-to-fight-covid-19/ (accessed on 19 March 2021).
83. Morocco launches fleet of drones to tackle virus from the sky. *France 24* 2020 Available online: www.france24.com/en/20200506-morocco-launches-fleet-of-drones-to-tackle-virus-from-the-sky (accessed on 26 March 2021).
84. Drones disinfect Indian coronavirus hotspot city after clashes. *SBS News* 2020 Available online: www.sbs.com.au/news/drones-disinfect-indian-coronavirus-hotspot-city-after-clashes (accessed on 30 March 2021).
85. Kleinman, Z. Coronavirus: Should the UK use drones to disinfect public spaces? *British Broadcasting Corporation (BBC)* 2020 Available online: www.bbc.co.uk/news/health-52109824 (accessed on 15 March 2021).
86. Kassam, A. Spanish official apologises for spraying beach with bleach. *The Guardian* 2020 Available online: www.theguardian.com/world/2020/apr/28/spanish-official-apologises-for-spraying-beach-with-bleach-coronavirus (accessed on 22 March 2020).
87. Migrants in India sprayed with disinfectant to fight coronavirus. *Al Jazeera* 2020 Available online: www.aljazeera.com/economy/2020/3/30/migrants-in-india-sprayed-with-disinfectant-to-fight-coronavirus (accessed on 20 April 2021).
88. Bergen, R. Babies developed blisters, swelling after touching disinfected shopping carts, Manitoba moms say. *Canadian Broadcasting Corporation (CBC)* 2020 Available online: www.cbc.ca/news/canada/manitoba/rash-shopping-cart-dauphin-manitoba-1.5585417 (accessed on 5 April 2021).
89. PNP investigating death of police doctor exposed to disinfectant. *CNN Philippines* 2020 Available online: https://cnnphilippines.com/news/2020/6/7/PNP-probe-police-doctor-death-disinfectant.html (accessed on 7 April 2021).
90. To fight coronavirus, disinfectant tunnel in China sprays industrial workers. *Reuters* 2020 Available online: www.reuters.com/article/us-china-health-disinfectant/to-fight-coronavirus-disinfectant-tunnel-in-china-sprays-industrial-workers-idUSKBN2061UU (accessed on 14 March 2020).
91. Disinfection Tunnel installed at Entrance of Sarajevo Centre Municipality. *Sarajevo Times* 2020 Available online: www.sarajevotimes.com/disinfection-tunnel-installed-at-entrance-of-sarajevo-centre-municipality/ (accessed on 20 April 2021).
92. Farzan, Z. SLAF designed "Passenger Disinfection Chamber" commissioned at BIA. *News First* 2020 Available online: www.newsfirst.lk/2020/04/28/slaf-designed-passenger-disinfection-chamber-commissioned-at-bia/ (accessed on 20 April 2021).
93. Khan, Y.H. …at the end of the tunnels. *The News on Sunday* 2020 Available online: www.thenews.com.pk/tns/detail/649238-at-the-end-of-the-tunnels (accessed on 20 April 2021).
94. Chile market gets 'Tunnel of Life' to battle virus. *SBS News* 2020 Available online: www.sbs.com.au/news/chile-market-gets-tunnel-of-life-to-battle-virus (accessed on 20 April 2021).
95. Hernandez, D. Mexican border town uses 'sanitizing tunnels' to disinfect US visitors from Covid-19. *The Guardian* 2020 Available online: www.theguardian.com/world/2020/may/11/mexico-us-sanitizing-tunnels-coronavirus-covid19 (accessed on 20 April 2021).
96. Mahmud, A.H. Disinfectant tunnel at Singapore Expo care facility on trial for safety, effectiveness against COVID-19. *Channel News Asia* 2020 Available online: www.channelnewsasia.com/news/singapore/covid-19-disinfectant-tunnel-singapore-expo-trial-who-12759900 (accessed on 20 April 2021).

97. Vietnam is ready for school reopening with disinfectant tunnels built by students to combat COVID-19. *Princess Maha Chakri Award* 2020 Available online: www.pmca.or.th/eng/?p=2156 (accessed on 20 April 2021).
98. Stieb, M. QAnon Influencers Are Encouraging Their Followers to Drink Bleach to Stave Off Coronavirus. *New York Magazine* 2020 Available online: https://nymag.com/intelligencer/2020/01/qanon-supporters-are-drinking-bleach-to-fend-off-coronavirus.html (accessed on 14 March 2021).
99. Evans, R. The International Church of Drink Bleach (Parts One and Two). *Behind the Bastards* **2016**.
100. 'Miracle' Treatment Turns into Potent Bleach. *US Food & Drug Administration* 2010 Available online: https://wayback.archive-it.org/7993/20170111070843/http:/www.fda.gov/ForConsumers/ConsumerUpdates/ucm228052.htm (accessed on 20 April 2021).
101. Speare-Cole, R. France forced to tell citizens that cocaine will not cure coronavirus amid social media rumours. *Evening Standard* 2020 Available online: www.standard.co.uk/news/world/france-coronavirus-cocaine-social-media-disinformation-a4383181.html (accessed on 14 March 2021).
102. Coronavirus (COVID-19) Update: FDA Warns Seller Marketing Dangerous Chlorine Dioxide Products that Claim to Treat or Prevent COVID-19. **2020**.
103. Burke, K. Coronavirus ‘bleach’ cure banned in the US, still selling in Australia. *7 News* 2020 Available online: https://7news.com.au/lifestyle/health-wellbeing/coronavirus-bleach-cure-banned-in-the-us-still-selling-in-australia--c-1023299 (accessed on 30 March 2021).
104. Tobin, G. Church in Australia selling bleach as a coronavirus treatment fined $151,200. *Australian Broadcasting Corporation (ABC)* 2020 Available online: www.abc.net.au/news/2020-05-13/church-in-australia-selling-bleach-coronavirus-treatment-fined/12242150 (accessed on 5 April 2021).
105. Strongman, S. Covid-19: NZ man selling bleach 'cure' slammed by scientists. *Radio New Zealand* 2020 Available online: www.rnz.co.nz/news/in-depth/417762/covid-19-nz-man-selling-bleach-cure-slammed-by-scientists (accessed on 6 April 2021).
106. Strongman, S. Covid-19 bleach 'cure' sold by New Zealand man rusts steel. *Radio New Zealand* 2020 Available online: www.rnz.co.nz/news/in-depth/419150/covid-19-bleach-cure-sold-by-new-zealand-man-rusts-steel (accessed on 7 April 2021).
107. Ballogg, R. Feds say Bradenton family got rich selling bleach as cure for COVID-19, other illnesses. *The Bradenton Herald* 2020 Available online: www.bradenton.com/article244122777.html (accessed on 8 April 2021).
108. Mielhan, P.; Asmelash, L. Florida man and his sons charged with selling toxic chemical as a coronavirus cure to thousands. *Cable News Network (CNN)* 2020 Available online: https://edition.cnn.com/2020/07/11/us/florida-men-coronavirus-cure-trnd/index.html (accessed on 8 April 2021).
109. Chlorine dioxide, the dangerous chemical that is touted as a “cure” for covid-19. *The Yucatan Times* 2020 Available online: www.theyucatantimes.com/2020/07/chlorine-dioxide-the-dangerous-chemical-that-is-touted-as-a-cure-for-covid-19/ (accessed on 8 April 2021).
110. Zúñiga, A. Please don’t take sodium chlorite or chlorine dioxide to treat COVID-19, Health Ministry says. *The Tico Times* 2020 Available online: https://ticotimes.net/2020/07/30/please-dont-take-sodium-chlorite-or-chlorine-dioxide-to-treat-covid-19-health-ministry-says (accessed on 9 April 2021).
111. Porter, T. Bolivians are drinking a toxic bleach, wrongly believing it will ward off COVID-19. Many end up in the hospital. *Business Insider* 2020 Available online: www.businessinsider.com/bolivians-drink-toxic-bleach-mms-believing-combat-coronavirus-2020-9?r=US&IR=T (accessed on 12 April 2021).
112. Desperate Bolivians seek out toxic bleach falsely touted as Covid-19 cure. *The Guardian* 2020 Available online: www.theguardian.com/world/2020/jul/17/bolivia-coronavirus-toxic-bleach-chlorine-dioxide (accessed on 8 April 2021).
113. Gigova, R. Lawmakers push toxic disinfectant as Covid-19 treatment in Bolivia, against Health Ministry's warnings. *CNN* 2020 Available online: https://edition.cnn.com/2020/07/29/americas/bolivia-disinfectant-covid-19-intl/index.html (accessed on 9 April 2020).
114. Porter, T. Bolivia's lawmakers have approved 'Miracle Mineral Solution' — a toxic bleach — as a treatment for COVID-19. Only the president can stop it becoming law. *Business Insider* 2020 Available online:

www.businessinsider.com/bolivian-lower-house-approves-toxic-bleach-as-covid-19-treatment-2020-8?r=US&IR=T (accessed on 11 April 2021).
115. García, B. COVID: Pastors of 'Church of the Bleach' arrested in Colombia. *Al Dia* 2020 Available online: https://aldianews.com/articles/culture/health/covid-pastors-church-bleach-arrested-colombia/60079 (accessed on 11 April 2021).
116. Braga, M. 'You have no authority over us': Church leader, sons jailed for insisting chemical's power to heal trumps laws of the state. *USA Today* 2020 Available online: https://eu.usatoday.com/story/news/investigations/2020/09/04/church-leader-sons-jailed-defying-fda-touting-coronavirus-cure/5660825002/ (accessed on 20 April 2021).
117. PAHO warns against use of chlorine products as treatments for COVID-19. *Pan American Health Organization* 2020 Available online: www.paho.org/en/news/5-8-2020-paho-warns-against-use-chlorine-products-treatments-covid-19 (accessed on 11 April 2021).
118. Alert: Chlorine Dioxide Ingestion. *The Georgia Department of Public Health* 2020 Available online: https://dph.georgia.gov/alert-chlorine-dioxide-ingestion (accessed on 12 April 2021).
119. Pilkington, E. Bleach touted as 'miracle cure' for Covid being sold on Amazon. *The Guardian* 2020 Available online: www.theguardian.com/world/2020/sep/19/bleach-miracle-cure-amazon-covid (accessed on 12 April 2021).
120. Covid patient who took chlorine dioxide dies in hospital. *Buenos Aires Times* 2021 Available online: www.batimes.com.ar/news/argentina/covid-19-patient-dies-after-judge-permits-private-clinic-to-administer-chlorine-dioxide.phtml (accessed on 22 April 2021).
121. Porter, T.; Moynihan, Q. Officials in Argentina raided labs producing toxic bleach as a COVID-19 treatment after a misinformation campaign fueled its popularity. *Business Insider* 2021 Available online: www.businessinsider.com/argentina-authorities-raid-labs-toxic-bleach-coronavirus-treatment-2021-3?r=US&IR=T (accessed on 22 April 2021).
122. The civil guard arrests a woman in Piedratajada for selling a "drink", without sanitary control, which is attributed benefits for the cure of COVID-19 and other pathologies. *The Spanish Journal* 2020 Available online: (accessed on 8 April 2021).
123. Lynn, G.; Carpenter, K. Coronavirus: Vendors exposed selling bleach as remedy. *British Broadcasting Corporation (BBC)* 2020 Available online: www.bbc.co.uk/news/uk-england-london-54162273 (accessed on 12 April 2020 2021).
124. Wallen, J. India gas leak kills at least 13 as 1,000 left in hospital. *The Telegraph* 2020 Available online: www.telegraph.co.uk/news/2020/05/07/chemical-gas-leak-india-kills-8-makes-1000-sick/ (accessed on 23 April 2021).
125. Boyle, L. Immigrants who speak out against being doused in toxic disinfectant for Covid at Trump-funded detention centre face retaliation, activists says. *The Independent UK* 2020 Available online: www.independent.co.uk/climate-change/news/immigrants-ice-detention-trump-border-wall-covid-chemicals-a9670771.html (accessed on 12 April 2021).
126. Chennai-Based Ayurvedic Pharmacist Dies After Drinking Formula He Prepared to 'Cure' Coronavirus. *News 18 India* 2020 Available online: www.news18.com/news/india/chennai-based-ayurvedic-pharmacist-dies-after-drinking-chemical-he-prepared-to-cure-coronavirus-2612689.html (accessed on 30 March 2021).
127. Philippines' Duterte recommends petrol to clean face masks, says 'not joking'. *Reuters* 2020 Available online: www.reuters.com/article/us-health-coronavirus-philippines-dutert/philippines-duterte-recommends-petrol-to-clean-face-masks-says-not-joking-idUSKCN24W1W6 (accessed on 9 April 2021).
128. Cosier, S. Activists shut down these cancer-causing plants. COVID is bringing them back. *Grist* 2020 Available online: https://grist.org/justice/ethylene-oxide-sterigenics-medline-covid/ (accessed on 8 April 2021).
129. Potentially toxic masks distributed in schools and daycares in Quebec. *Canadian Broadcasting Corporation (CBC)* 2021 Available online: www.cbc.ca/news/canada/montreal/masks-early-pulmonary-toxicity-quebec-schools-daycares-1.5966387 (accessed on 22 April 2021).
130. Bouzi, A. Face masks given out by Belgian government may contain toxic particles. *Politico* 2021 Available online: www.politico.eu/article/free-masks-distributed-by-belgian-government-contain-toxic-particles/ (accessed on 26 April 2021).

131. Seven paper mill workers fall ill in Chhattisgarh after inhaling toxic gas. *The Hindu* 2020 Available online: www.thehindu.com/news/national/seven-paper-mill-workers-fall-ill-in-chhattisgarh-after-inhaling-toxic-gas/article31528570.ece (accessed on 30 March 2021).
132. Jeffords, S. 'Two crises': Ontario's opioid problem worsens during COVID-19 as services for drug users scale back. *Canadian Broadcasting Corporation (CBC)* 2020 Available online: www.cbc.ca/news/canada/toronto/ontario-opioid-covid19-1.5551368 (accessed on 23 March 2021).
133. Gibson, V. 'They have big challenges there': Ontario's top medical officer draws contrast to overdose crisis in B.C. *iPolitics* 2020 Available online: https://ipolitics.ca/2020/05/07/they-have-big-challenges-there-ontarios-top-medical-officer-draws-contrast-to-overdose-crisis-in-b-c/ (accessed on 26 March 2021).
134. Smith, A. Illicit drug supply potentially more dangerous amid COVID-19 pandemic. *Calgary Herald* 2020 Available online: https://calgaryherald.com/news/illicit-drug-supply-potentially-more-dangerous-amid-covid-19-pandemic/ (accessed on 5 April 2021).
135. Kovach, J. Rash of overdose deaths in Peterborough this week. *The Peterborough Examiner* 2020 Available online: www.thepeterboroughexaminer.com/news/peterborough-region/2020/05/27/rash-of-overdose-deaths-in-peterborough-this-week.html (accessed on 5 April 2021).
136. Rellinger, P. Rash of overdose deaths eclipse COVID-19 recovery as Peterborough medical officer of health's major worry. *Kawartha Now* 2020 Available online: https://kawarthanow.com/2020/06/24/rash-of-overdose-deaths-eclipse-covid-19-recovery-as-peterborough-medical-officer-of-healths-major-worry/ (accessed on 7 April 2021).
137. Gallardo, M. Chicago area opioid overdose deaths spike amid COVID-19 pandemic. *7 Eyewitness News ABC* 2020 Available online: https://abc7chicago.com/coronavirus-chicago-opioid-death-overdose-opiod/6316666/ (accessed on 8 April 2021).
138. Community mourns after 14-year-old dies of overdose on Vancouver Island. *CTV News* 2020 Available online: https://vancouverisland.ctvnews.ca/community-mourns-after-14-year-old-dies-of-overdose-on-vancouver-island-1.5027225 (accessed on 8 April 2021).
139. Preliminary patterns in circumstances surrounding opioid-related deaths in Ontario during the COVID-19 pandemic. *Ontario Drug Policy Research Network* 2020 Available online: (accessed on 21 April 2021).
140. Alberta substance use surveillance system. **2020**.
141. Toronto Public Health reports record number of overdose deaths. *City of Toronto* 2021 Available online: www.toronto.ca/news/toronto-public-health-reports-record-number-of-overdose-deaths/ (accessed on 22 April 2021).
142. Jonas, S. Paramedics attend a record 40 suspected opioid overdose calls, 3 deaths in 24 hours. *Canadian Broadcasting Corporation (CBC)* 2021 Available online: www.cbc.ca/news/canada/toronto/toronto-record-number-opioid-deaths-december-1.5894780 (accessed on 22 April 2021).
143. Pikulicka-Wilczewska, A. COVID-19 Adds to Addiction Woes in Afghanistan. *VOA News* 2020 Available online: www.voanews.com/south-central-asia/covid-19-adds-addiction-woes-afghanistan (accessed on 8 April 2020).
144. Rosenfield, D. SickKids sees rise in unintentional poisonings in children during COVID-19 pandemic. *Toronto Star* 2021 Available online: www.thestar.com/opinion/contributors/2021/03/22/sickkids-sees-rise-in-unintentional-poisonings-in-children-during-covid-19-pandemic.html (accessed on 26 April 2021).
145. Jones, S. As Coronavirus Panic Heats Up, So Do Sales of Snake Oil. *New York Magazine* 2020 Available online: https://nymag.com/intelligencer/2020/03/essential-oils-and-other-coronavirus-scam-and-hoax-cures.html (accessed on 14 March 2021).
146. Fortin, J. That 'Miracle Cure' You Saw on Facebook? It Won't Stop the Coronavirus. *The New York Times* 2020 Available online: www.nytimes.com/2020/03/18/health/coronavirus-cure-gargle-water.html (accessed on 15 March 2021).
147. O'Connor, A. Supplements for Coronavirus Probably Won't Help, and May Harm. *The New York Times* 2020 Available online: www.nytimes.com/2020/03/23/well/live/coronavirus-supplements-herbs-vitamins-colds-flu.html (accessed on 15 March 2021).
148. Office of Public Affairs. Court Orders Halt to Sale of Silver Product Fraudulently Touted as COVID-19 Cure. **2020**.

149. Stockler, A. Federal Judge Orders Oklahoma Company to Stop Selling Colloidal Silver as a Coronavirus Treatment. *Newsweek* 2020 Available online: www.newsweek.com/colloidal-silver-coronavirus-food-drug-administration-1504460 (accessed on 24 March 2021).
150. Chandna, H. Too much turmeric, methi, vitamin D — Doctors fight new emergencies driven by Covid fear. *The Print* 2020 Available online: https://theprint.in/health/too-much-turmeric-methi-vitamin-d-doctors-fight-new-emergencies-driven-by-covid-fear/495557/ (accessed on 12 April 2021).
151. Pari, U. Medicine or candy? A deadly mistake in the age of coronavirus. *KSAT* 2020 Available online: www.ksat.com/news/local/2020/07/23/medicine-or-candy-a-deadly-mistake-in-the-age-of-coronavirus/ (accessed on 8 April 2021).
152. Wee, S.-L. In Coronavirus, China Weighs Benefits of Buffalo Horn and Other Remedies. *The New York Times* 2020 Available online: www.nytimes.com/2020/02/05/world/asia/coronavirus-traditional-chinese-medicine.html (accessed on 15 March 2021).
153. Cyranoski, D. China is promoting coronavirus treatments based on unproven traditional medicines. *Nature* 2020 Available online: www.nature.com/articles/d41586-020-01284-x (accessed on 26 March 2021).
154. Cachia, A. Madagascar vows to 'change the history of the entire world' as it carries out tests on mystery plant it claims can cure coronavirus. *The Daily Mail* 2020 Available online: www.dailymail.co.uk/news/article-8204131/Plant-remedy-virus-tested-says-Madagascar-leader.html (accessed on 19 March 2021).
155. 2020. Coronavirus: What is Madagascar's 'herbal remedy' Covid-Organics? *Al Jazeera* Available online: www.aljazeera.com/news/2020/5/5/coronavirus-what-is-madagascars-herbal-remedy-covid-organics (accessed on 24 March 2021).
156. Madagascar ships anti-virus potion to Guinea-Bissau. *Yahoo News* 2020 Available online: https://news.yahoo.com/madagascar-ships-anti-virus-potion-guinea-bissau-185848499.html (accessed on 23 March 2021).
157. Rabary, L. Madagascar coronavirus herbal mix draws demand from across Africa despite WHO misgivings. *Reuters* 2020 Available online: www.reuters.com/article/uk-health-coronavirus-madagascar-idUKKBN22K1IK (accessed on 30 March 2021).
158. May 29: Retreat on COVID-Organics injectables. *Africa News* 2020 Available online: www.africanews.com/2020/07/29/madagascar-prez-dismisses-all-critics-would-they-doubt-european-cure// (accessed on 6 April 2021).
159. Medsafe blocks sales of Covid-19 'treatment' Artemisia annua. *Radio New Zealand* 2020 Available online: www.rnz.co.nz/news/national/416971/medsafe-blocks-sales-of-covid-19-treatment-artemisia-annua (accessed on 5 April 2021).
160. Madagascar leader backs herbal 'cure' over Covid vaccines. *Radio Television Luxembourg* 2021 Available online: https://today.rtl.lu/news/world/a/1692377.html (accessed on 21 March 2021).
161. Health experts refute claim that ancient medicinal herbs are an effective coronavirus remedy. *AFP Factcheck* 2020 Available online: https://factcheck.afp.com/health-experts-refute-claim-ancient-medicinal-herbs-are-effective-coronavirus-remedy (accessed on 14 March 2021).
162. Chandna, H. Modi govt wants states to start producing herbal remedy for Covid-19 immunity, sends recipe. *The Print* 2020 Available online: https://theprint.in/india/modi-govt-wants-states-to-start-producing-herbal-remedy-for-covid-19-immunity-sends-recipe/408931/ (accessed on 22 March 2021).
163. Cameroon: Mgr Samuel Kleda affirms Coronavirus herbal remedy is effective. *Journal du Cameroun* 2020 Available online: www.journalducameroun.com/en/cameroon-mgr-samuel-kleda-affirms-coronavirus-herbal-remedy-is-effective/ (accessed on 23 March 2021).
164. Nneka. Nigerian Catholic priest develops drug for Covid-19 cure. *The Daily Times* 2020 Available online: https://dailytimes.ng/just-in-nigerian-catholic-priest-fr-anslem-develops-drug-for-covid-19-cure/ (accessed on 23 March 2021).
165. Kindzeka, M.E. Hundreds rush for Archbishop's herbal COVID 'cure' in Cameroon. *VOA News* 2020 Available online: www.voanews.com/covid-19-pandemic/hundreds-rush-popular-clerics-herbal-covid-cure-cameroon (accessed on 23 March 2021).

166. Cameroon archbishop says treating COVID-19 with plant-based remedy. *Reuters* 2020 Available online: www.reuters.com/article/us-health-coronavirus-cameroon-treatment/cameroon-archbishop-says-treating-covid-19-with-plant-based-remedy-idUKKBN23N28K (accessed on 7 April 2021).
167. Siddique, A.; Sarwar, S.; Parsa, N. Afghan Gov't Warns Against Popular But Unproven Coronavirus Treatment. *Gandhara* 2020 Available online: https://gandhara.rferl.org/a/afghan-gov-t-warns-against-popular-but-unproven-coronavirus-treatment/30646578.html (accessed on 6 April 2021).
168. Kumar, R. In Afghanistan, a Drug Sparks Hope – and Charges of Quackery. *The Wire* 2020 Available online: https://science.thewire.in/health/in-afghanistan-a-drug-sparks-hope-and-charges-of-quackery/ (accessed on 11 April 2021).
169. Jayachandran, N. After Kerala man gets liver disease, docs warn against bogus COVID-19 herbal drinks. *The News Minute* 2020 Available online: www.thenewsminute.com/article/after-kerala-man-gets-liver-disease-docs-warn-against-bogus-covid-19-herbal-drinks-127030 (accessed on 7 April 2021).
170. Zimbabweans seek traditional medicine amid pandemic. *The Independent* 2020 Available online: www.independent.co.ug/zimbabweans-seek-traditional-medicine-amid-pandemic/ (accessed on 9 April 2021).
171. Ettang, I. Nigerian Biotechnologist Touts Potent Herbal COVID-19 Treatment. *VOA News* 2020 Available online: www.voanews.com/covid-19-pandemic/nigerian-biotechnologist-touts-potent-herbal-covid-19-treatment (accessed on 12 April 2021).
172. Maduro draws flak for promoting 'miracle' COVID remedy. *Deutsche Welle* 2021 Available online: www.dw.com/en/maduro-draws-flak-for-promoting-miracle-covid-remedy/a-56356325 (accessed on 22 April 2021).
173. Thousands in Sri Lanka drink 'miracle' COVID potion, minister ill. *Al Jazeera* 2021 Available online: www.aljazeera.com/news/2021/1/20/thousands-in-sri-lanka-drink-miracle-covid-potion-minister-ill (accessed on 22 April 2021).
174. Leighfield, J. Thousands queue in Sri Lanka to get Covid-19 'miracle cure'. *EuroWeekly* 2020 Available online: www.euroweeklynews.com/2020/12/09/thousands-queue-in-sri-lanka-to-get-covid-19-miracle-cure/ (accessed on 21 April 2021).
175. Matreu, B. Tanzanians urged to use traditional medicine for Covid-19. *The East African* 2021 Available online: www.theeastafrican.co.ke/tea/news/east-africa/tanzania-traditional-medicine-covid-19-3276402 (accessed on 22 April 2021).
176. Graziosi, G. Trump allies push for FDA to approve another untested Covid treatment - extract from toxic oleander plant. *The Independent UK* 2020 Available online: www.independent.co.uk/news/world/americas/us-politics/covid-treatment-oleander-plant-donald-trump-fda-hydroxychloroquine-coronavirus-a9675006.html (accessed on 12 April 2021).
177. American College of Medical Toxicology; American Academy of Clinical Toxicology; American Association of Poison Control Centers. Leading Clinical Toxicology Organizations Issue Joint Statement on Dangers of Oleandrin Toxicity. *CISION* 2020 Available online: www.prweb.com/releases/leading_clinical_toxicology_organizations_issue_joint_statement_on_dangers_of_oleandrin_toxicity/prweb17342824.htm (accessed on 12 April 2021).
178. Seddiq, O. Ben Carson says he took an unproven coronavirus treatment touted by MyPillow's CEO after testing positive for the disease. *Business Insider* 2020 Available online: www.businessinsider.com/ben-carson-unproven-covid-19-treatment-mypillow-mike-lindell-oleander-2020-11?r=US&IR=T (accessed on 21 April 2021).
179. Rhodes, W. Oleander extract sales rocket after Ben Carson touts it for COVID. But is it safe? *Palm Beach Post* 2020 Available online: https://eu.palmbeachpost.com/story/news/politics/state/2020/11/23/recovered-covid-19-ben-carson-touts-benefits-oleander-extract/6389614002/ (accessed on 23 April 2021).
180. White, N.; Wilkie, K. Inside Pete Evans' new social media empire: How the celebrity chef turned wellness guru is making a FORTUNE by sharing dangerous conspiracy theories along with his recipes. *The Daily Mail* 2020 Available online: www.dailymail.co.uk/news/article-8680171/How-Pete-Evans-make-fortune-posting-coronavirus-conspiracy-theories-recipes.html (accessed on 12 April 2021).

181. Chung, L. 'Hope in treatments': Spike in imports of unproven COVID-19 drugs. *Sydney Morning Herald* 2020 Available online: www.smh.com.au/national/nsw/hope-in-treatments-spike-in-imports-of-drugs-believed-to-treat-covid-19-20200729-p55gly.html (accessed on 11 April 2021).
182. Perchick, M. More people getting snake bites in central North Carolina as they spend time outdoors during COVID-19 pandemic. *11 Eyewitness News* 2020 Available online: https://abc11.com/snake-bite-common-snakes-in-nc-venomous-eastern-diamondback-rattlesnake/6280484/ (accessed on 8 April 2021).
183. California Poison Control System. California Poison Control offers rattlesnake bite prevention tips. *Orange County Breeze* 2020 Available online: www.oc-breeze.com/2020/07/29/185354_california-poison-control-offers-rattlesnake-bite-prevention-tips/ (accessed on 9 April 2021).
184. Shapiro, E. Snake bites on the rise: How to stay safe. *American Broadcasting Company (ABC)* 2020 Available online: https://abcnews.go.com/US/snake-bites-rise-stay-safe/story?id=71424874 (accessed on 8 April 2021).
185. Broom, B. Just how likely are you to be bitten or die from a venomous snake encounter in MS? *Mississippi Clarion Ledger* 2020 Available online: https://eu.clarionledger.com/story/life/2020/08/02/snakes-mississippi-odds-on-being-bitten-dying-from-snakebite/5525702002/ (accessed on 10 April 2021).
186. McNaughton, J. Wild mushroom foraging booms during coronavirus pandemic, as poison hotline calls spike. *Australian Broadcasting Corporation (ABC)* 2020 Available online: www.abc.net.au/news/rural/2020-05-12/mushroom-forage-coronavirus-death-cap-poison-hotline/12232412 (accessed on 5 April 2021).
187. Murray, S. Is this mushroom safe? Expert fields more concerned calls during pandemic. *Tallahassee Democrat* 2020 Available online: https://eu.tallahassee.com/story/life/home-garden/2020/05/21/mushroom-safe-expert-fields-concerned-calls/5230131002/ (accessed on 5 April 2021).
188. Turner, L. Mushroom foraging on the rise in northwestern Ontario as COVID-19 boosts interest in the activity. *Canadian Broadcasting Corporation (CBC)* 2020 Available online: www.cbc.ca/news/canada/thunder-bay/mushroom-foraging-tbay-2020-1.5717972 (accessed on 12 April 2021).
189. Beware of poisoning when foraging for wild mushrooms! *ANSES* 2020 Available online: www.anses.fr/en/content/beware-poisoning-when-foraging-wild-mushrooms (accessed on 21 April 2021).
190. Boseley, M. Poisonous mushrooms in full bloom across Victoria after heavy rains spur growth. *The Guardian* 2021 Available online: www.theguardian.com/australia-news/2021/mar/31/poisonous-mushrooms-in-full-bloom-across-victoria-after-heavy-rains-spur-growth (accessed on 22 April 2021 2021).
191. Allison, D. 'Nasty' Giant Hogweed thrives as lockdown cuts treatment. *British Broadcasting Corporation (BBC)* 2020 Available online: www.bbc.co.uk/news/uk-scotland-52726060 (accessed on 5 April 2021).
192. Fitzpatrick, T. Toxic plant that can cause blindness and third degree burns thriving in Scotland. *The Daily Record* 2020 Available online: www.dailyrecord.co.uk/news/scottish-news/toxic-plant-can-cause-blindness-22207105 (accessed on 7 April 2021).
193. Armstrong, G. Toxic plant warning for Glasgow parents and dog owners as giant hogweed lies untreated. *Glasgow Live* 2020 Available online: www.glasgowlive.co.uk/news/glasgow-news/toxic-plant-warning-glasgow-parents-18711697 (accessed on 11 April 2021).
194. Vaidyanathan, V. Several People Hospitalized After Trying TikTok 'Home Remedies' To Keep Coronavirus Away. *International Business Times* 2020 Available online: www.ibtimes.com/several-people-hospitalized-after-trying-tiktok-home-remedies-keep-coronavirus-away-2955663 (accessed on 15 March 2021).
195. Langlois, J. 'Hydroxychloroquine tea' is being peddled as a coronavirus cure in Brazil. It's fake. *National Geographic* 2020 Available online: www.nationalgeographic.co.uk/science-and-technology/2020/06/hydroxychloroquine-tea-is-being-peddled-as-a-coronavirus-cure-in (accessed on 6 April 2021).
196. Boal, J. Utahns Warned To Watch For Toxic Algal Blooms. *KSL TV* 2020 Available online: https://ksltv.com/440685/utahns-warned-to-watch-for-toxic-algal-blooms/ (accessed on 8 April 2021).
197. Williams, A.B. Blue-green algae task force members tackle plenty in first meeting in almost 10 months. *Fort Myers News-Press* 2020 Available online: https://eu.news-press.com/story/tech/science/environment/2020/07/29/blue-green-algae-task-force-members-tackle-algae-warnings-first-meeting-since-october/5511427002/ (accessed on 9 April 2021).

198. Turkmenistan president claims licorice can cure coronavirus. *Daily Sabah* 2020 Available online: www.dailysabah.com/world/asia-pacific/turkmenistan-president-claims-licorice-can-cure-coronavirus (accessed on 23 April 2021).
199. O'Leary, M.E. New Haven official: Identifying lead poisoned children was delayed by coronavirus shutdown. *New Haven Register* 2020 Available online: www.ctinsider.com/news/nhregister/article/New-Haven-official-Identifying-lead-poisoned-15401496.php (accessed on 8 April 2021).
200. Busari, S.; Adebayo, B. Nigeria records chloroquine poisoning after Trump endorses it for coronavirus treatment. *Cable News Network (CNN)* 2020 Available online: https://edition.cnn.com/2020/03/23/africa/chloroquine-trump-nigeria-intl/index.html (accessed on 14 March 2021).
201. Coronavirus: World leaders' posts deleted over fake news. *British Broadcasting Corporation (BBC)* 2020 Available online: www.bbc.co.uk/news/technology-52106321 (accessed on 14 March 2021).
202. Scott, S.; Timms, P. Malaria and arthritis drugs touted as potential coronavirus 'cure', triggering pharmacy rush. *Australian Broadcasting Corporation (ABC)* 2020 Available online: www.abc.net.au/news/2020-03-23/malaria-drugs-labelled-early-coronavirus-covid19-cures-treatment/12081306?nw=0 (accessed on 14 March 2021).
203. Parashar, U. Assam doctor who took anti-malaria drug amid Covid-19 outbreak dies. *Hindustan Times* 2020 Available online: www.msn.com/en-in/news/newsindia/assam-doctor-who-took-anti-malaria-drug-amid-covid-19-outbreak-dies/ar-BB11Tywb (accessed on 15 March 2021).
204. Piranty, S. Coronavirus fuels a surge in fake medicines. *British Broadcasting Corporation (BBC)* 2020 Available online: www.bbc.co.uk/news/health-52201077 (accessed on 15 March 2021).
205. Owens, B. Excitement around hydroxychloroquine for treating COVID-19 causes challenges for rheumatology. *The Lancet Rheumatology* **2020**, *2*, e247, doi:10.1016/S2665-9913(20)30089-8.
206. Hydroxychloroquine: The unproven 'corona drug' Trump is threatening India for. *British Broadcasting Corporation (BBC)* 2020 Available online: www.bbc.co.uk/news/world-asia-india-52180660 (accessed on 15 March 2021).
207. Side effects seen with use of chloroquine against COVID-19. *Medical Xpress* 2020 Available online: https://medicalxpress.com/news/2020-04-side-effects-chloroquine-covid-.html (accessed on 15 March 2021).
208. Trials halted for drug stockpiled in Israel as potential COVID-19 treatment. *Times of Israel* 2020 Available online: www.timesofisrael.com/trials-halted-for-drug-stockpiled-in-israel-as-potential-covid-19-treatment/ (accessed on 19 March 2021).
209. Osborne, H. French Hospital Stops Hydroxychloroquine Treatment for COVID-19 Patient Over Major Cardiac Risk. *Newsweek* 2020 Available online: www.newsweek.com/hydroxychloroquine-coronavirus-france-heart-cardiac-1496810 (accessed on 15 March 2021).
210. Borba, M.G.S.; Val, F.F.A.; Sampaio, V.S.; Alexandre, M.A.A.; Melo, G.C.; Brito, M.; Mourão, M.P.G.; Brito-Sousa, J.D.; Baía-da-Silva, D.; Guerra, M.V.F.; et al. Effect of High vs Low Doses of Chloroquine Diphosphate as Adjunctive Therapy for Patients Hospitalized With Severe Acute Respiratory Syndrome Coronavirus 2 (SARS-CoV-2) Infection: A Randomized Clinical Trial. *JAMA Network Open* **2020**, *3*, e208857-e208857, doi:10.1001/jamanetworkopen.2020.8857 %J JAMA Network Open.
211. Glenza, J. Brazilian chloroquine study halted after high dose proved lethal for some patients. *The Guardian* 2020 Available online: www.theguardian.com/world/2020/apr/24/chloroquine-study-coronavirus-brazil (accessed on 22 March 2021).
212. Sandler, R. Hydroxychloroquine Abuse Up Since Trump First Mentioned Drug, U.S. Poison Center Data Says. *Forbes* 2020 Available online: www.forbes.com/sites/rachelsandler/2020/04/09/hydroxychloroquine-abuse-up-since-trump-first-mentioned-drug-us-poison-centers-say/ (accessed on 19 March 2021).
213. Przybyla, H. Family of New York woman blames hydroxychloroquine combo for fatal heart attack. *NBC News* 2020 Available online: www.nbcnews.com/health/health-news/family-new-york-woman-blames-hydroxychloroquine-combo-fatal-heart-attack-n1185451 (accessed on 19 March 2021).
214. Sheth, S. DOJ began investigating a doctor promoting unproven COVID-19 treatments after Roger Stone's former associate accidentally emailed a federal prosecutor instead of the doctor. *Business Insider* 2020

Available online: www.businessinsider.com/doj-investigates-doctor-pushing-questionable-coronavirus-treatments-after-accidental-email-2020-5?r=US&IR=T (accessed on 23 March 2021).
215. Hydroxychloroquine seized at the border. **2020**.
216. Vella, V.; Roebuck, J.; Bender, W. Pennsylvania nursing homes are treating coronavirus patients with the unproven malaria drug Trump touted — sometimes without consent. *The Philadelphia Inquirer* 2020 Available online: www.inquirer.com/news/coronavirus-nursing-homes-pennsylvania-hydroxychloroquine-pennsylvania-broomall-southeastern-veterans-center-20200511.html (accessed on 5 April 2021).
217. Yen, H. VA says it won't stop use of unproven drug on vets for now. *American Broadcasting Company (ABC)* 2020 Available online: https://abcnews.go.com/Health/wireStory/va-wont-stop-unproven-drug-vets-now-70711408 (accessed on 5 April 2021).
218. Parker, M. Trump Finishes Controversial Malaria Drug Course to Combat Virus. *Bloomberg* 2020 Available online: www.bloomberg.com/news/articles/2020-05-24/trump-finishes-controversial-malaria-drug-course-to-combat-virus (accessed on 5 April 2021).
219. Roberts, M. Coronavirus: Trump says he is taking unproven drug hydroxychloroquine. *British Broadcasting Corporation (BBC)* 2020 Available online: www.bbc.co.uk/news/world-us-canada-52717161 (accessed on 5 April 2021).
220. Bolsonaro says Brazil to issue new chloroquine protocol on Wednesday. *Reuters* 2020 Available online: www.reuters.com/article/us-health-coronavirus-brazil-bolsonaro-idUSKBN22V39L (accessed on 5 April 2021).
221. France revokes decree authorising use of hydroxychloroquine to treat Covid-19. *France 24* 2020 Available online: www.france24.com/en/20200527-france-revokes-decree-authorising-use-of-hydroxychloroquine-to-treat-covid-19 (accessed on 5 April 2021).
222. Indonesia to keep prescribing two malaria drugs for COVID-19 despite bans in Europe. *Reuters* 2020 Available online: www.reuters.com/article/us-health-coronavirus-indonesia-drugs-idUSKBN2341XG (accessed on 6 April 2021).
223. Yen, H. VA says it'll stop almost all use of unproven drug on vets. *American Broadcasting Company (ABC)* 2020 Available online: https://abcnews.go.com/Health/wireStory/va-stop-unproven-drug-vets-70935087 (accessed on 6 April 2021).
224. Davey, M. WHO to resume hydroxychloroquine trial after earlier halt over safety concerns. *The Guardian* 2020 Available online: www.theguardian.com/world/2020/jun/04/who-to-resume-hydroxychloroquine-trial-after-earlier-halt-over-safety-concerns (accessed on 6 April 2021).
225. "Solidarity" clinical trial for COVID-19 treatments. *World Health Organization* 2020 Available online: www.who.int/emergencies/diseases/novel-coronavirus-2019/global-research-on-novel-coronavirus-2019-ncov/solidarity-clinical-trial-for-covid-19-treatments (accessed on 20 May 2021).
226. Hydroxychloroquine: US revokes emergency approval of malaria drug for Covid-19. *The Guardian* 2020 Available online: www.theguardian.com/world/2020/jun/15/hydroxychloroquine-coronavirus-fda-emergency-authorization (accessed on 7 April 2021).
227. Pedroso, R.; Arias, T.; Picheta, R. Brazil swipes at FDA, pushes hydroxychloroquine for pregnant women and children. *Cable News Network (CNN)* 2020 Available online: https://edition.cnn.com/2020/06/16/americas/brazil-hydroxychloroquine-recommendations-fda-intl/index.html (accessed on 7 April 2021).
228. For Brazil's Bolsonaro: A Week of Isolation, Hydroxychloroquine. *VOA News* 2020 Available online: www.voanews.com/covid-19-pandemic/brazils-bolsonaro-week-isolation-hydroxychloroquine (accessed on 8 April 2021).
229. Griffith, J. Florida teen attended large church gathering, was given hydroxychloroquine by parents before dying from COVID-19, report says. *NBC News* 2020 Available online: www.nbcnews.com/news/us-news/florida-teen-attended-large-church-gathering-was-given-hydroxychloroquine-parents-n1233091 (accessed on 8 April 2021).
230. Hodge, A. Red flags as Indonesian university claims it has COVID-19 cure. *The Australian* 2020 Available online: www.theaustralian.com.au/world/red-flags-as-indonesian-university-claims-it-has-covid19-cure/news-story/5a41873e178bb9389ff633e798952a7f (accessed on 12 April 2021).

231. Karp, P. Labor condemns Craig Kelly's suggestion Daniel Andrews could 'face 25 years in jail' for hydroxychloroquine ban. *The Guardian* 2020 Available online: www.theguardian.com/australia-news/2020/aug/03/labor-condemns-craig-kellys-suggestion-daniel-andrews-could-face-25-years-in-jail-for-hydroxychloroquine-ban (accessed on 10 April 2021).
232. Murphy, K. 'Outlandish': Labor urges Facebook to act against Craig Kelly's 'harmful' content. *The Guardian* 2021 Available online: www.theguardian.com/australia-news/2021/feb/03/craig-kellys-covid-claims-labor-urges-facebook-to-take-action-against (accessed on 22 April 2021).
233. Reimann, N. Doctor Charged In Fake Covid 'Miracle Cure' Scheme Selling $3,995 Kits With Hydroxychloroquine Pills He Got For $1. *Forbes* 2020 Available online: www.forbes.com/sites/nicholasreimann/2020/12/03/doctor-charged-in-fake-covid-miracle-cure-scheme-selling-3995-kits-with-hydroxychloroquine-pills-he-got-for-1/ (accessed on 23 April 2021).
234. McCoy, T. My wife and I got covid-19. Our doctor prescribed a medication used to treat parasites in livestock. *The Washington Post* 2021 Available online: www.washingtonpost.com/world/the_americas/coronavirus-brazil-covid-kit/2021/02/12/8328f56a-6632-11eb-8468-21bc48f07fe5_story.html (accessed on 23 April 2021).
235. Sommerland, J. Brazil's Bolsonaro branded 'psychopathic leader' as three die from taking 'Covid kit' he promoted. *The Independent UK* 2021 Available online: www.independent.co.uk/news/world/americas/jair-bolsonaro-coronavirus-kit-deaths-brazil-b1821238.html (accessed on 23 April 2021).
236. Warning issued as researchers reveal another potential treatment for coronavirus. *Australian Broadcasting Corporation (ABC)* 2020 Available online: www.abc.net.au/news/2020-04-04/monash-researchers-hopes-for-coronavirus-drug-treatment-warning/12121578 (accessed on 15 March 2021).
237. Caution about Laboratory COVID-19 Report. **2020**.
238. Boyd, A. Lice drug a head-scratching Covid-19 home remedy. *Asia Times* 2020 Available online: https://asiatimes.com/2020/04/lice-drug-a-head-scratching-covid-19-home-remedy/ (accessed on 15 March 2021).
239. Unproven Covid 'cure' gets big dose of coverage. *Radio New Zealand* 2020 Available online: www.rnz.co.nz/national/programmes/mediawatch/audio/2018762446/unproven-covid-cure-gets-big-dose-of-coverage (accessed on 12 April 2021).
240. Offord, C. Surgisphere Sows Confusion About Another Unproven COVID-19 Drug. *The Scientist* 2020 Available online: www.the-scientist.com/news-opinion/surgisphere-sows-confusion-about-another-unproven-covid19-drug-67635 (accessed on 7 April 2021).
241. Mega, E.R. Latin America's embrace of an unproven COVID treatment is hindering drug trials. *Nature* 2020 Available online: www.nature.com/articles/d41586-020-02958-2 (accessed on 21 April 2021).
242. 'Miracle' drug ivermectin unproven against Covid, scientists warn. *France 24* 2020 Available online: www.france24.com/en/live-news/20210115-miracle-drug-ivermectin-unproven-against-covid-scientists-warn (accessed on 22 April 2021).
243. Farber, T. Social media hype over unproven Ivermectin is irresponsible — experts. *Sunday Times* 2021 Available online: www.timeslive.co.za/news/south-africa/2021-01-20-social-media-hype-over-unproven-ivermectin-is-irresponsible-experts/ (accessed on 22 April 2021).
244. R6 million worth of tablets seized at ORTIA. *South African Police Service* 2021 Available online: www.saps.gov.za/newsroom/msspeechdetail.php?nid=30683 (accessed on 22 April 2021).
245. Widmer, S. Do-it-yourself COVID treatments an evolving threat. *American Broadcasting Company (ABC)* 2021 Available online: https://abcnews.go.com/Health/covid-treatments-evolving-threat/story?id=75946569 (accessed on 22 April 2021).
246. FactCheck: Is the medication ivermectin an alternative to Covid-19 vaccines? *The Journal* 2021 Available online: www.thejournal.ie/factcheck-ivermectin-covid-19-5385782-Mar2021/ (accessed on 23 April 2021).
247. Ivermectin is not a cure for COVID-19, says the European Medicines Agency. *Euro News* 2021 Available online: www.euronews.com/2021/03/25/ivermectin-is-not-a-cure-for-covid-19-says-the-european-medicines-agency (accessed on 23 April 2021).
248. Ivermectin: South African medics using unproven worm drug to treat Covid-19. *British Broadcasting Corporation* 2021 Available online: www.bbc.co.uk/news/world-africa-56526632 (accessed on 26 April 2021).

249. Washington Poison Center. Washington Poison Center reports increased opioid exposures during COVID-19. *CISION* 2021 Available online: www.prnewswire.com/news-releases/washington-poison-center-reports-increased-opioid-exposures-during-covid-19-301251178.html (accessed on 26 April 2021).
250. Dooley, B. This Drug May Cause Birth Defects. Japan's Pushing It for Coronavirus. *The New York Times* 2020 Available online: www.nytimes.com/2020/05/05/business/japan-avigan-coronavirus.html (accessed on 24 March 2021).
251. Weise, E. Could heartburn drug famotidine be used to treat coronavirus? The theory is trending online, but there's no data to support it. *USA Today* 2020 Available online: https://eu.usatoday.com/story/news/health/2020/04/28/pepcid-treat-covid-19-theory-trending-online-coronavirus/3043325001/ (accessed on 23 March 2021).
252. Tanno, S.; Simpson, L. Amazon, Walgreens and CVS run short of heartburn medicine Pepcid as people stock up after it emerged it was being tested as a treatment for Covid-19. *The Daily Mail* 2020 Available online: www.dailymail.co.uk/news/article-8264905/People-buying-heartburn-medicine-thats-tested-coronavirus-treatment.html (accessed on 23 March 2021).
253. FDA Letter to Stakeholders: Do Not Use Ivermectin Intended for Animals as Treatment for COVID-19 in Humans. **2020**.
254. FAQ: COVID-19 and Ivermectin Intended for Animals. *US Food & Drug Administration* 2021 Available online: www.fda.gov/animal-veterinary/product-safety-information/faq-covid-19-and-ivermectin-intended-animals (accessed on 22 April 2021).
255. Phillips, L. COVID-19 fears cause rush on ivermectin. *Farmers Weekly* 2021 Available online: www.farmersweekly.co.za/agri-news/south-africa/covid-19-fears-cause-rush-on-ivermectin/ (accessed on 22 April 2021).
256. Qukula, Q. Black market Ivermectin remains a concern despite compassionate-use approval. *Cape Talk* 2020 Available online: www.capetalk.co.za/articles/407576/black-market-ivermectin-remains-a-concern-despite-compassionate-use-approval (accessed on 22 April 2021).
257. Whyte, S. Leyonhjelm rapped by medicines regulator for horse medicine tweets. *Port Macquarie News* 2021 Available online: www.portnews.com.au/story/7120101/leyonhjelm-rapped-by-medicines-regulator-for-horse-medicine-tweets/?cs=17267 (accessed on 22 April 2021).
258. Why You Should Not Use Ivermectin to Treat or Prevent COVID-19. *US Food & Drug Administration* 2021 Available online: www.fda.gov/consumers/consumer-updates/why-you-should-not-use-ivermectin-treat-or-prevent-covid-19 (accessed on 22 April 2021).
259. Mkhize, N. Estcourt family in critical condition after ingesting poison to cure COVID-19. *East Coast Radio KZN* 2020 Available online: www.ecr.co.za/news/news/estcourt-family-critical-condition-after-ingesting-poison-cure-covid-19/ (accessed on 7 April 2021).
260. Friedman, L. E.P.A. Threatens Legal Action Against Sellers of Fake Coronavirus Cleaners. *The New York Times* 2020 Available online: www.nytimes.com/2020/04/03/climate/epa-fake-coronavirus-cleaners.html (accessed on 6 April 2021).
261. Kovalev, A.; Rothrock, K. The Kremlin's virus blocker *Meduza* 2020 Available online: https://meduza.io/en/feature/2020/04/10/the-kremlin-s-virus-blocker (accessed on 19 March 2021).
262. Mwai, P. Coronavirus: The misinformation circulating in Africa about Covid-19. *British Broadcasting Corporation (BBC)* 2020 Available online: www.bbc.co.uk/news/world-africa-52819674 (accessed on 7 April 2021).
263. Cockerell, I. Bolivia's president wore a "virus blocker." Then, she got Covid-19. *Coda* 2020 Available online: www.codastory.com/waronscience/bolivia-virus-blocker-badge/ (accessed on 8 April 2021).
264. Dlouhy, J.A. EPA Tells Amazon, EBay to Stop Shipping Unproven Covid Goods. *Bloomberg* 2020 Available online: www.bloomberg.com/news/articles/2020-06-11/epa-orders-amazon-ebay-to-stop-shipping-unproven-covid-products (accessed on 7 April 2021).
265. Livingstone, T. Preschoolers caught with potentially harmful 'COVID-19 protector'. *9 News* 2020 Available online: https://www.9news.com.au/national/coronavirus-school-students-bringing-potentially-dangerous-virus-shut-out-device-to-school/116ccc3e-df9c-4ada-ad0a-1bcc1588c6c0 (accessed on 7 April 2021).

266. Soeriaatmadja, W. Eucalyptus necklace and other unproven Covid-19 cures raise alarm in Indonesia. *The Straits Times* 2020 Available online: www.straitstimes.com/asia/se-asia/eucalyptus-necklace-and-other-unproven-covid-19-cures-raise-alarm-in-indonesia (accessed on 26 April 2021).
267. Paddock, R.C. In Indonesia, False Virus Cures Pushed by Those Who Should Know Better. *The New York Times* 2020 Available online: www.nytimes.com/2020/07/31/world/asia/indonesia-coronavirus.html (accessed on 26 April 2021).
268. Kitching, C. Brit 'sold fake coronavirus cure with "harmful chemicals" to Americans for £40'. *The Mirror* 2020 Available online: www.mirror.co.uk/news/us-news/brit-sold-fake-coronavirus-cure-21799165 (accessed on 15 March 2021).
269. UK medicines and medical devices regulator investigating 14 cases of fake or unlicensed COVID-19 medical products. **2020**.
270. ICE HSI Baltimore seizes over 14,000 unapproved COVID-19 treatment capsules, several unapproved test kits. **2020**.
271. Europol predictions correct for fake COVID-19 vaccines. *Europol* 2021 Available online: www.europol.europa.eu/newsroom/news/europol-predictions-correct-for-fake-covid-19-vaccines (accessed on 26 April 2021).
272. Loon, A. Myanmar to crack down on unregistered COVID-19 cures and remedies. *Myanmar Times* 2021 Available online: www.mmtimes.com/news/myanmar-crack-down-unregistered-covid-19-cures-and-remedies.html (accessed on 22 April 2021).
273. Police search for man who injected 92-year-old with fake Covid vaccine. *The Guardian* 2020 Available online: www.theguardian.com/uk-news/2021/jan/08/police-search-for-man-who-injected-92-year-old-with-fake-covid-vaccine (accessed on 22 April 2021).
274. Purported biotech executive charged with introducing misbranded drug into interstate commerce for distribution of "COVID-19 vaccine". *US Department of Justice* 2021 Available online: www.justice.gov/usao-wdwa/pr/purported-biotech-executive-charged-introducing-misbranded-drug-interstate-commerce (accessed on 22 April 2021).
275. Operation Stolen Promise One-Year Anniversary. *U.S. Immigration and Customs Enforcement* 2021 Available online: www.ice.gov/topics/operation-stolen-promise (accessed on 1 February 2021).
276. Family in S. Korea admitted to hospital after using methanol to disinfect home. *Inquirer.net* 2020 Available online: https://newsinfo.inquirer.net/1246561/family-in-s-korea-admitted-to-hospital-after-using-methanol-to-disinfect-home (accessed on 14 March 2021).
277. Joo, J.T. Sources: Almost 200 soldiers have died from COVID-19. *Daily NK* 2020 Available online: www.dailynk.com/english/sources-almost-200-soldiers-have-died-covid-19/ (accessed on 30 April 2021).
278. Erburu-Iriarte, M.; Rodrigo-Armenteros, P.; Oyarzun-Irazu, I.; Aranzabal-Alustiza, I.; Silvarrey-Rodriguez, S.; Antón-Méndez, L.; García-Moncó, J.C. Chronic severe methanol intoxication after repeated mask cleansing due to fear of COVID-19: A new risk of coronaphobia. *European journal of neurology* **2021**, doi:10.1111/ene.14779.
279. Ilyushina, M.; Hodge, N. Post-Soviet strongmen prescribe vodka, hockey and folk medicine against coronavirus. *Cable News Network (CNN)* 2020 Available online: https://edition.cnn.com/2020/03/30/europe/soviet-strongmen-coronavirus-intl/index.html (accessed on 15 March 2021).
280. Tidman, Z. Coronavirus: Kenya governor under fire after putting cognac in care packages. *The Independent* 2020 Available online: www.independent.co.uk/news/world/africa/coronavirus-kenya-hennessy-care-packages-nairobi-mike-sonko-a9472076.html (accessed on 19 March 2021).
281. DiMeglio, S. John Daly on vodka/smokes COVID-19 cure: 'I was just doing it for fun'. *Golf Week USA Today* 2020 Available online: https://golfweek.usatoday.com/2020/04/28/john-daly-on-vodka-smokes-covid-19-cure-i-was-just-doing-it-for-fun/ (accessed on 23 March 2021).
282. Woods, M. No, alcohol doesn't kill the coronavirus. *CTV* 2020 Available online: (accessed on 5 April 2021).
283. Local gin not cure for Covid-19 – NCCE cautions public. *Atlantic Federation of African Press Agencies (FAAPA)* 2020 Available online: www.faapa.info/en/2020/06/01/local-gin-not-cure-for-covid-19-ncce-cautions-public/ (accessed on 6 April 2021).

284. Mangaluru councillor in trouble for recommending Rum & Eggs as COVID-19 home remedy. *Mangalore Today* 2020 Available online: www.mangaloretoday.com/main/Mangaluru-councillor-in-trouble-for-recommending-Rum-Eggs-as-COVID-19-home-remedy.html (accessed on 8 April 2021).
285. Alcohol and cannabis use during the pandemic: Canadian Perspectives Survey Series 6. *Statistics Canada* 2021 Available online: www150.statcan.gc.ca/n1/daily-quotidien/210304/dq210304a-eng.htm (accessed on 26 April 2021).
286. Vanderbruggen, N.; Matthys, F.; Van Laere, S.; Zeeuws, D.; Santermans, L.; Van den Ameele, S.; Crunelle, C.L. Self-Reported Alcohol, Tobacco, and Cannabis Use during COVID-19 Lockdown Measures: Results from a Web-Based Survey. *Eur Addict Res* **2020**, *26*, 309-315, doi:10.1159/000510822.
287. Arunogiri, S.; Gurvich, C.; Kulkarni, J. Women are drinking more during the pandemic, and it's probably got a lot to do with their mental health. *The Conversation* 2020 Available online: https://theconversation.com/women-are-drinking-more-during-the-pandemic-and-its-probably-got-a-lot-to-do-with-their-mental-health-139295 (accessed on 6 April 2021).
288. Clent, D. Coronavirus: One in seven Kiwis drinking more than nomal post-lockdown. *Stuff Limited* 2020 Available online: www.stuff.co.nz/national/health/122258468/coronavirus-one-in-seven-kiwis-drinking-more-than-nomal-postlockdown (accessed on 9 April 2021).
289. Grierson, J. Half of British drinkers starting earlier in the day during Covid-19 crisis. *The Guardian* 2020 Available online: www.theguardian.com/society/2020/jun/02/half-of-british-drinkers-starting-earlier-in-the-day-during-covid-19-crisis (accessed on 6 April 2021).
290. Niedzwiedz, C.L.; Green, M.J.; Benzeval, M.; Campbell, D.; Craig, P.; Demou, E.; Leyland, A.; Pearce, A.; Thomson, R.; Whitley, E.; et al. Mental health and health behaviours before and during the initial phase of the COVID-19 lockdown: longitudinal analyses of the UK Household Longitudinal Study. *Journal of Epidemiology and Community Health* **2021**, *75*, 224, doi:10.1136/jech-2020-215060.
291. Alcohol deaths hit record high during Covid pandemic. *British Broadcasting Corporation (BBC)* 2021 Available online: www.bbc.co.uk/news/health-55900624 (accessed on 22 April 2021).
292. Wider impacts of COVID-19 on health monitoring tool. *Public Health England* 2021 Available online: www.gov.uk/government/statistics/wider-impacts-of-covid-19-on-health-monitoring-tool#alcohol-smoking-gambling-and-physical-activity (accessed on 23 April 2021).
293. Stevely, A.; Sasso, A.; Alava, M.H.; Holmes, J. Changes in alcohol consumption in Scotland during the early stages of the COVID-19 pandemic: Descriptive analysis of repeat cross-sectional survey data. *Public Health Scotland* 2021 Available online: www.publichealthscotland.scot/media/2983/changes-in-alcohol-consumption-in-scotland-during-the-early-stages-of-the-covid-19-pandemic.pdf (accessed on 26 April 2021).
294. Rodriguez, L.M.; Litt, D.M.; Stewart, S.H. Drinking to cope with the pandemic: The unique associations of COVID-19-related perceived threat and psychological distress to drinking behaviors in American men and women. *Addict Behav* **2020**, *110*, 106532-106532, doi:10.1016/j.addbeh.2020.106532.
295. Chan, E. As alcohol abuse rises amid pandemic, hospitals see a wave of deadly liver disease. *Los Angeles Times* 2021 Available online: www.latimes.com/california/story/2021-02-08/alcohol-abuse-pandemic-hospitals-liver-disease (accessed on 22 April 2021).
296. Weerakoon, S.M.; Jetelina, K.K.; Knell, G. Longer time spent at home during COVID-19 pandemic is associated with binge drinking among US adults. *The American Journal of Drug and Alcohol Abuse* **2021**, *47*, 98-106, doi:10.1080/00952990.2020.1832508.
297. Barbosa, C.; Cowell, A.J.; Dowd, W.N. Alcohol Consumption in Response to the COVID-19 Pandemic in the United States. *Journal of addiction medicine* **2020**, doi:10.1097/adm.0000000000000767.
298. Wilson, C. *The Herals Scotland* 2020 Available online: www.heraldscotland.com/news/18531372.concern-lockdown-drinking-doctors-report-spike-liver-damage-cases/ (accessed on 7 April 2021).
299. 'Soaring alcohol misuse' could overwhelm service. *British Broacasting Corporation (BBC)* 2020 Available online: www.bbc.co.uk/news/health-54151481 (accessed on 12 April 2021).
300. Cahan, E. Pandemic-fueled alcohol abuse creates wave of hospitalizations for liver disease. *The Philadelphia Inquirer* 2021 Available online: www.inquirer.com/health/coronavirus/liver-disease-increase-pandemic-alcohol-20210212.html (accessed on 26 April 2021).

301. Torr, G.; Prest, V. Huge spike in demand for alcohol treatment 'due to pandemic'. *Yorkshire Live* 2021 Available online: www.examinerlive.co.uk/news/local-news/huge-spike-demand-alcohol-treatment-19830543 (accessed on 26 April 2021).
302. 30 die after drinking pure alcohol to ward off coronavirus in Istanbul, 20 more hospitalized. *Daily Sabah* 2020 Available online: www.dailysabah.com/turkey/30-die-after-drinking-pure-alcohol-to-ward-off-coronavirus-in-istanbul-20-more-hospitalized/news (accessed on 14 March 2021).
303. Karimi, N.; Gambrell, J. Hundreds die in Iran believing methanol drinking can fight the coronavirus. *The Globe and Mail* 2020 Available online: www.theglobeandmail.com/world/article-hundreds-killed-in-iran-over-false-belief-that-drinking-methanol-can/ (accessed on 21 April 2021).
304. Jones, M. Tragic link between North Wales teacher's coronavirus tonic and hundreds of deaths in Iran. *The Daily Post* 2020 Available online: www.dailypost.co.uk/news/north-wales-news/tragic-link-between-north-wales-18019185 (accessed on 15 March 2021).
305. Brown, L. UK teacher claims he beat coronavirus with 'hot whisky and honey'. *The New York Post* 2020 Available online: https://nypost.com/2020/02/03/uk-teacher-claims-he-beat-coronavirus-with-hot-whisky-and-honey/ (accessed on 21 April 2021).
306. Trew, B. Coronavirus: Hundreds dead in Iran from drinking methanol amid fake reports it cures disease. *Indepentent* 2020 Available online: www.independent.co.uk/news/world/middle-east/iran-coronavirus-methanol-drink-cure-deaths-fake-a9429956.html (accessed on 15 March 2021).
307. Mehrpour, O.; Sadeghi, M. Toll of acute methanol poisoning for preventing COVID-19. *Archives of Toxicology* **2020**, *94*, 2259-2260, doi:10.1007/s00204-020-02795-2.
308. Death toll in latest Mexico alcohol poisoning rises to 18. *American Broadcasting Company (ABC)* 2020 Available online: https://abcnews.go.com/International/wireStory/death-toll-latest-mexico-alcohol-poisoning-rises-18-71143487 (accessed on 7 April 2021).
309. Mexico: at least 35 people die in mass bad-alcohol poisoning. *The Guardian* 2020 Available online: www.theguardian.com/world/2020/may/13/mexico-mass-alcohol-poisoning-deaths-methanol (accessed on 5 April 2021).
310. Agren, D. Mexico: adulterated alcohol deaths rise to over 100 amid ban on official sales. *The Guardian* 2020 Available online: www.theguardian.com/world/2020/may/15/mexico-adulterated-alcohol-deaths-over-100-coronavirus (accessed on 5 April 2021).
311. Chothia, A. Northern Cape couple allegedly die after drinking homemade beer. *The South African* 2020 Available online: www.thesouthafrican.com/news/northern-cape-couple-allegedly-die-homemade-beer/ (accessed on 24 March 2021).
312. Meyer, D. Second couple believed to have been killed by homemade beer. *The South African* 2020 Available online: www.thesouthafrican.com/news/couple-killed-homemade-beer-is-it-safe-to-drink-lockdown-2020/ (accessed on 5 April 2021).
313. Isaacs, L.; Siyo, A. Seven die from home-made alcohol in Masiphumelele. *Independent Online (IOL)* 2020 Available online: www.iol.co.za/capetimes/news/seven-die-from-home-made-alcohol-in-masiphumelele-48953921 (accessed on 6 April 2021).
314. Young, N. South Africa's long-standing alcohol abuse problem reemerged a day after lifting a lockdown ban. *Quartz Africa* 2020 Available online: https://qz.com/africa/1863922/south-africa-alcohol-abuse-up-after-lockdown-sales-ban/ (accessed on 6 April 2021).
315. Two young women die from homemade alcohol in the Free State. *South African Broadcasting Corporation (SABC)* 2021 Available online: (accessed on 21 April 2021).
316. Bouscaren, D. Pandemic's deadly side effect: Global rise in bootleg alcohol poisonings. *The World* 2020 Available online: www.pri.org/stories/2020-10-20/pandemic-s-deadly-side-effect-global-rise-bootleg-alcohol-poisonings (accessed on 23 April 2021).
317. Shawon, A.A. The root of Bangladesh's alcohol poisoning outbreak. *Dhaka Tribune* 2021 Available online: www.dhakatribune.com/bangladesh/crisis/2021/02/06/the-root-of-bangladesh-s-alcohol-poisoning-outbreak (accessed on 22 April 2021).

318. Kindzeka, M.E. Cameroon Says Deaths from Consuming Fake Alcohol Spike During Pandemic. *VOA News* 2021 Available online: www.voanews.com/africa/cameroon-says-deaths-consuming-fake-alcohol-spike-during-pandemic (accessed on 26 April 2021).
319. Kumar, V. TV Actors Commit Suicide in Chennai, Decomposed Bodies Found After Neighbours Complained of Foul Smell. *India.com* 2020 Available online: www.india.com/entertainment/tamil-news-tv-actors-commit-suicide-in-chennai-decomposed-bodies-found-after-neighbours-complaint-of-foul-smell-4050645/ (accessed on 7 April 2021).
320. Kakvi, K. In MP's Khandwa, 27 people committed suicide in one month due to exams stress, financial crisis and job loss. *National Herald India* 2020 Available online: www.nationalheraldindia.com/india/in-mps-khandwa-27-people-committed-suicide-in-one-month-due-to-exams-stress-financial-crisis-and-job-loss (accessed on 7 April 2021).
321. Elderly woman, two sons 'attempt suicide' in Kolkata flat. *The Tribune India* 2020 Available online: www.tribuneindia.com/news/nation/elderly-woman-two-sons-attempt-suicide-in-kolkata-flat-104853 (accessed on 8 April 2021).
322. Cousins commit suicide. *The Hindu* 2020 Available online: www.thehindu.com/news/cities/Madurai/cousins-commit-suicide-thoothukudi/article31724091.ece (accessed on 6 April 2021).
323. Wagle, M. Nearly 40 suicide deaths reported amid COVID-19 lockdown in Tanahun. *The Himalayan Times* 2020 Available online: https://thehimalayantimes.com/nepal/nearly-40-suicide-deaths-reported-amid-covid-19-lockdown-in-tanahun (accessed on 8 April 2021).
324. Suicides on the rise as young Iraqis pushed to despair. *The Arab Weekly* 2020 Available online: https://thearabweekly.com/suicides-rise-young-iraqis-pushed-despair (accessed on 8 April 2021).
325. Baba, A. Non-COVID patients continue to suffer in north Kashmir. *Greater Kashmir* 2020 Available online: www.greaterkashmir.com/news/kashmir/non-covid-patients-continue-to-suffer-in-north-kashmir/ (accessed on 8 April 2021).
326. Bengaluru: Businessman found dead, police suspect Covid fear the trigger. *Times of India* 2020 Available online: https://timesofindia.indiatimes.com/city/bengaluru/bengaluru-businessman-found-dead-police-suspect-covid-fear-the-trigger/articleshow/76905154.cms (accessed on 8 April 2021).
327. Hyderabad couple with fever ends life over coronavirus fear. *The Indian Express* 2020 Available online: www.newindianexpress.com/cities/hyderabad/2020/aug/02/hyderabad-couple-with-fever-ends-life-over-coronavirus-fear-2177929.html (accessed on 10 April 2021).
328. Taunted by villagers, 65-year-old Covid patient ends life in Karnataka. *Times of India* 2020 Available online: https://timesofindia.indiatimes.com/city/bengaluru/taunted-by-villagers-65-year-old-covid-patient-ends-life-in-karnataka/articleshow/77324562.cms (accessed on 11 April 2021).
329. Man uses poison, packed as corona medicine, in bid to get rival murdered. *Hindustan Times* 2020 Available online: www.hindustantimes.com/delhi-news/man-uses-poison-packed-as-corona-medicine-in-bid-to-get-rival-murdered/story-2WFMo0Zr3MbTNYiOBUyuFM.html (accessed on 5 April 2021).
330. Njunge, G.; Kabunyi, F. Mystery of toxic food aid given in 'Ruto bags'. *The Standard* 2020 Available online: www.standardmedia.co.ke/central/article/2001372736/mystery-of-toxic-food-aid-given-in-ruto-bags (accessed on 5 April 2021).
331. Pinto, N. Man poisons family, self in Karnataka's Dharwad district over fear of losing job. *India Today* 2020 Available online: www.indiatoday.in/india/story/man-poisons-family-self-in-karnataka-s-dharwad-district-over-fear-of-losing-job-1704530-2020-07-26 (accessed on 9 April 2021).
